# Supplementary material for: Prescribing quality in secondary care patients with different stages of chronic kidney disease: a retrospective study in the Netherlands
Source: BMJ Open. 2019 Jul 19;9(7):e025784. doi: 10.1136/bmjopen-2018-025784 (PMC6661701; doi:10.1136/bmjopen-2018-025784)
Supplement: Supplementary Table 1 [file bmjopen-2018-025784supp001.pdf]

S1 Table. Baseline characteristics per nephrology outpatient clinic.

	Clinic A (n=569)		Clinic B (n=845)		Clinic C (n=1,718)	
	**N (%)**	**Mean (±SD)**	**N (%)**	**Mean (±SD)**	**N (%)**	**Mean (±SD)**
Age (years)	569 (100)	63.4 (±14.6)	845 (100)	65.0 (±15.3)	1,718 (100)	70.5 (±12.6)
< 50 years	98 (17.2)		134 (15.9)		131 (7.6)	
50-80 years	397 (69.8)		581 (68.8)		1,171 (68.2)	
>= 80 years	74 (13.0)		130 (15.4)		416 (24.2)	
Gender (males)	323 (56.8)		446 (52.8)		969 (56.4)	
Diabetes (yes)	145 (25.5)		164 (19.4)		176 (10.2)	
eGFR (MDRD) ml/min	569 (100)	28.4 (±14.2)	845 (100)	35.9 (±14.1)	1,718 (100)	36.5 (±13.1)
Stage 3a	92 (16.2)		255 (30.2)		496 (28.9)	
Stage 3b	166 (29.2)		295 (34.9)		664 (38.7)	
Stage 4	183 (32.2)		219 (25.9)		460 (26.8)	
Stage 5	128 (22.5)		76 (9.0)		98 (5.7)	
SBP (mmHg)	552 (97.0)	131.7 (±18.6)	470 (55.6)	133.4 (±20.7)	1,489 (86.7)	131.7 (±18.2)
Elevated SBP (>140 mmHg)	137 (24.1)	156.2 (±12.7)	143 (16.9)	158.0 (±13.3)	420 (24.4)	154.0 (±11.8)
DBP (mmHg)	552 (97.0)	72.0 (±12.4)	470 (55.6)	76.0 (±10.7)	1,489 (86.7)	76.0 (±10.7)
Low DBP (<70 mmHg)	202 (35.5)	59.4 (±6.9)	100 (11.8)	61.7 (±4.4)	402 (23.4)	62.6 (±5.2)
Total protein (g/24h urine)	513 (90.2)	0.4 [0.1-1.2][a]	244 (28.9)	0.9 [0.3-2.1][a]	557 (32.4)	0.3 [0.1-0.9][a]
Total protein (g/l urine)	515 (90.5)	0.2 [0.1-0.7][a]	574 (67.9)	0.3 [0.1-0.7][a]	1,239 (72.1)	0.2 [0.1-0.4][a]
Proteinuria (>0.5 g/24h or L urine)	215 (37.8)		238 (28.2)		357 (20.8)	
Phosphate (mmol/l)	544 (95.6)	1.20 (±0.33)	623 (73.7)	1.10 (±0.32)	1,439 (83.8)	1.02 (±0.24)
Elevated phosphate (>1.49 mmol/l)	78 (13.7)	1.79 (±0.33)	49 (5.8)	1.87 (±0.40)	45 (2.6)	1.73 (±0.29)
Calcium (mmol/l)	546 (96.0)	2.35 (±0.17)	663 (78.5)	2.34 (±0.14)	1,525 (88.8)	2.37 (±0.12)
Elevated Calcium (>2.54 mmol/l)	35 (6.2)	2.62 (±0.06)	28 (3.3)	2.62 (±0.06)	100 (5.8)	2.62 (±0.09)
Haemoglobin level (mmol/l)	565 (99.3)	7.75 (±1.09)	808 (95.6)	8.0 (±1.2)	1,651 (96.1)	8.1 (±1.1)
Low haemoglobin level (<7.5 mmol/l)	222 (39.0)	6.7 (±0.6)	253 (29.9)	6.7 (±0.6)	458 (26.7)	6.8 (±0.6)

eGFR: estimated glomerular filtration rate; MDRD: Modification of Diet in Renal Disease; SBP: systolic blood pressure; DBP: diastolic blood pressure.

Clinic A and B: university nephrology outpatient clinics; clinic C: non-university nephrology outpatient clinic.

[a] Median with interquartile range.
