# Supplementary material for: Prescribing quality in secondary care patients with different stages of chronic kidney disease: a retrospective study in the Netherlands
Source: BMJ Open. 2019 Jul 19;9(7):e025784. doi: 10.1136/bmjopen-2018-025784 (PMC6661701; doi:10.1136/bmjopen-2018-025784)
Supplement: Supplementary Table 2 [file bmjopen-2018-025784supp002.pdf]

S2 Table. Baseline table per chronic kidney disease stage per location.

	Clinic A (N=92)		Clinic B (N=255)		Clinic C (N=496)	
**CKD stage 3a**	**N (%)**	**Mean (±SD)**	**N (%)**	**Mean (±SD)**	**N (%)**	**Mean (±SD)**
Age (years)	92 (100)	56.8 (±13.5)	255 (100)	60.5 (±14.9)	496 (100)	65.6 (±13.4)
< 50 years	26 (28.3)		56 (22.0)		69 (13.9)	
50-80 years	62 (67.4)		182 (71.4)		360 (72.6)	
>= 80 years	4 (4.4)		17 (6.7)		67 (13.5)	
Gender (males)	46 (50.0)		135 (52.9)		275 (55.4)	
Diabetes (yes)	14 (15.2)		39 (15.3)		43 (8.7)	
eGFR (MDRD) ml/min	92 (100)	51.1 (±4.2)	255 (100)	52.4 (±4.2)	496 (100)	52.3 (±4.4)
SBP (mmHg)	91 (98.9)	126.3 (±17.4)	122 (47.8)	128.5 (±18.8)	388 (78.2)	130.4 (±16.7)
Elevated SBP (>140 mmHg)	12 (13.0)	157.1 (±15.7)	23 (9.0)	157.4 (±12.9)	84 (16.9)	154.5 (±11.7)
DBP (mmHg)	91 (98.9)	73.4 (±12.5)	122 (47.8)	76.0 (±10.5)	388 (78.2)	77.6 (±10.0)
Low DBP (<70 mmHg)	31 (33.7)	59.8 (±6.1)	22 (8.6)	61.3 (±4.4)	77 (15.5)	63.2 (±5.2)
Total protein (g/24h urine)	84 (91.3)	0.2 [0.1-1.0][a]	62 (24.3)	0.5 [0.3-1.3][a]	132 (26.6)	0.2 [0.1-0.5][a]
Total protein (g/l urine)	84 (91.3)	0.1 [0.1-0.4][a]	165 (65.7)	0.1 [0.0-0.4][a]	303 (61.1)	0.1 [0.1-0.3][a]
Proteinuria (>0.5 g/24h or L urine)	29 (31.5)		52 (20.4)		59 (11.9)	
Phosphate (mmol/l)	83 (90.2)	1.02 (±0.20)	139 (54.5)	0.98 (±0.22)	317 (63.9)	0.94 (±0.19)
Elevated phosphate (>1.49 mmol/l)	0 (0.0)	-	2 (0.8)	1.72 (±0.30)	1 (0.2)	1.63 (-)
Calcium (mmol/l)	84 (91.3)	2.38 (±0.14)	159 (62.4)	2.37 (±0.11)	373 (75.2)	2.38 (±0.11)
Elevated Calcium (>2.54 mmol/l)	6 (6.5)	2.60 (±0.05)	7 (2.7)	2.62 (±0.06)	27 (5.4)	2.62 (±0.08)
Haemoglobin level (mmol/l)	90 (97.8)	8.4 (±1.2)	231 (90.6)	8.6 (±1.0)	450 (90.7)	8.4 (±1.0)
Low haemoglobin level (<7.5 mmol/l)	15 (16.3)	6.6 (±0.5)	29 (11.4)	6.8 (±0.6)	69 (13.9)	6.8 (±0.6)
**CKD stage 3b**	**Clinic A (N=166)**		**Clinic B (N=295)**		**Clinic C (N=664)**	
	**N (%)**	**Mean (±SD)**	**N (%)**	**Mean (±SD)**	**N (%)**	**Mean (±SD)**
Age	166 (100)	63.4 (±14.3)	295 (100)	66.9 (±14.8)	664 (100)	71.2 (±11.5)
< 50 years	33 (19.9)		40 (13.6)		40 (6.0)	
50-80 years	115 (69.3)		200 (67.8)		478 (72.0)	
>= 80 years	18 (10.8)		55 (18.6)		146 (22.0)	
Gender (males)	98 (59.0)		147 (49.8)		369 (55.6)	
Diabetes (yes)	44 (26.5)		54 (18.3)		68 (10.2)	
eGFR (MDRD) ml/min	166 (100)	36.4 (±4.4)	295 (100)	37.6 (±4.1)	664 (100)	37.4 (±4.2)

SBP (mmHg)	163 (98.2)	129.6 (±17.0)	160 (54.2)	131.6 (±21.0)	587 (88.4)	130.7 (±18.3)
Elevated SBP (>140 mmHg)	33 (19.9)	153.8 (±14.2)	49 (16.6)	156.6 (±12.9)	157 (23.4)	153.7 (±11.9)
DBP (mmHg)	163 (98.2)	72.4 (±12.4)	160 (54.2)	75.5 (±10.3)	587 (88.4)	75.4 (±11.1)
Low DBP (<70 mmHg)	61 (36.7)	60.2 (±6.6)	35 (11.9)	61.8 (±4.2)	186 (28.0)	62.8 (±5.0)
Total protein (g/24h urine)	148 (89.2)	0.2 [0.1-0.6][a]	67 (22.7)	0.7 [0.2-2.0][a]	211 (31.8)	0.2 [0.1-0.7][a]
Total protein (g/l urine)	150 (90.4)	0.2 [0.1-0.4][a]	190 (64.4)	0.2 [0.1-0.5][a]	501 (75.5)	0.2 [0.1-0.4][a]
Proteinuria (>0.5 g/24h or L urine)	39 (23.5)		63 (21.4)		122 (18.4)	
Phosphate (mmol/l)	160 (96.4)	1.04 (±0.21)	213 (72.2)	1.02 (±0.22)	586 (88.3)	0.98 (±0.19)
Elevated phosphate (>1.49 mmol/l)	5 (3.0)	1.58 (±0.04)	6 (2.0)	1.80 (±0.28)	6 (0.9)	1.57 (±0.08)
Calcium (mmol/l)	160 (96.4)	2.37 (±0.15)	231 (78.3)	2.34 (±0.12)	607 (91.4)	2.37 (±0.13)
Elevated Calcium (>2.54 mmol/l)	10 (6.0)	2.62 (±0.06)	7 (2.4)	2.61 (±0.06)	44 (6.6)	2.63 (±0.10)
Haemoglobin level (mmol/l)	166 (100)	8.1 (±1.0)	284 (96.3)	8.1 (±1.1)	645 (97.1)	8.2 (±1.0)
Low haemoglobin level (<7.5 mmol/l)	37 (22.2)	6.9 (±0.5)	81 (27.5)	6.8 (±0.5)	146 (22.0)	6.8 (±0.5)
**CKD stage 4**	**Clinic A (N=183)**		**Clinic B (N=219)**		**Clinic C (N=460)**	
	**N (%)**	**Mean (±SD)**	**N (%)**	**Mean (±SD)**	**N (%)**	**Mean (±SD)**
Age	183 (100)	65.3 (±14.6)	219 (100)	67.4 (±15.2)	460 (100)	73.4 (±11.8)
< 50 years	24 (13.1)		26 (11.9)		20 (4.4)	
50-80 years	126 (68.9)		149 (68.0)		281 (61.1)	
>= 80 years	33 (18.0)		44 (20.1)		159 (34.6)	
Gender (males)	98 (53.6)		119 (54.3)		271 (58.9)	
Diabetes (yes)	55 (30.1)		58 (26.5)		52 (11.3)	
eGFR (MDRD) ml/min	183 (100)	22.3 (±4.2)	219 (100)	23.0 (±4.3)	460 (100)	23.4 (±4.2)
SBP (mmHg)	176 (96.2)	132.0 (±19.4)	140 (63.9)	136.8 (±21.0)	419 (91.1)	132.6 (±18.7)
Elevated SBP (>140 mmHg)	48 (26.2)	156.8 (±11.4)	50 (22.8)	159.1 (±13.8)	135 (29.3)	153.5 (±11.5)
DBP (mmHg)	176 (96.2)	71.6 (±12.8)	140 (63.9)	75.1 (±10.9)	419 (91.1)	75.7 (±10.3)
Low DBP (<70 mmHg)	66 (36.1)	59.0 (±7.6)	38 (17.4)	61.7 (±4.7)	110 (23.9)	62.6 (±5.1)
Total protein (g/24h urine)	163 (89.1)	0.4 [0.1-1.0][a]	75 (34.2)	0.9 [0.3-2.3][a]	165 (35.9)	0.4 [0.2-1.3][a]
Total protein (g/l urine)	163 (89.1)	0.2 [0.1-0.6][a]	162 (74.0)	0.3 [0.2-0.8][a]	362 (78.7)	0.2 [0.1-0.6][a]
Proteinuria (>0.5 g/24h or L urine)	63 (34.4)		77 (35.2)		128 (27.8)	
Phosphate (mmol/l)	176 (96.2)	1.18 (±0.23)	200 (91.3)	1.14 (±0.32)	439 (95.4)	1.05 (±0.2)
Elevated phosphate (>1.49 mmol/l)	16 (8.7)	1.62 (±0.11)	17 (7.8)	1.84 (±0.47)	8 (1.7)	1.66 (±0.11)
Calcium (mmol/l)	176 (96.2)	2.35 (±0.20)	199 (90.9)	2.34 (±0.16)	447 (97.2)	2.36 (±0.12)
Elevated Calcium (>2.54 mmol/l)	13 (7.1)	2.61 (±0.07)	9 (4.1)	2.65 (±0.07)	24 (5.2)	2.62 (±0.06)

Haemoglobin level (mmol/l)	182 (99.5)	7.6 (±1.0)	217 (99.1)	7.7 (±1.2)	458 (99.6)	7.8 (±1.0)
Low haemoglobin level (<7.5 mmol/l)	82 (44.8)	6.7 (±0.7)	89 (40.6)	6.6 (±0.6)	175 (38.0)	6.7 (±0.6)
**CKD stage 5**	**Clinic A (N=128)**		**Clinic B (N=76)**		**Clinic (N=98)**	
	**N (%)**	**Mean (±SD)**	**N (%)**	**Mean (±SD)**	**N (%)**	**Mean (±SD)**
Age	128 (100)	65.5 (±14.7)	100 (76)	65.5 (±15.9)	100 (98)	76.9 (±10.6)
< 50 years	15 (11.7)		12 (15.8)		2.0 (2.0)	
50-80 years	94 (73.4)		40 (65.8)		52 (53.1)	
>= 80 years	19 (14.8)		14 (18.4)		44 (44.9)	
Gender (males)	81 (63.3)		45 (59.2)		54 (55.1)	
Diabetes (yes)	32 (25.0)		13 (17.1)		13 (13.3)	
eGFR (MDRD) ml/min	128 (100)	10.5 (±2.6)	76 (100)	11.2 (±2.4)	98 (100)	11.9 (±2.6)
SBP (mmHg)	122 (95.3)	137.9 (±18.6)	48 (63.2)	142.1 (±19.8)	95 (96.9)	139.2 (±19.7)
Elevated SBP (>140 mmHg)	44 (34.4)	157.1 (±12.2)	21 (27.6)	159.6 (±14.2)	44 (44.9)	155.9 (±12.2)
DBP (mmHg)	122 (95.3)	71.1 (±11.6)	48 (63.2)	80.6 (±11.4)	95 (96.9)	74.4 (±11.9)
Low DBP (<70 mmHg)	44 (34.4)	58.5 (±6.7)	5 (6.6)	62.2 (±4.1)	29 (29.6)	60.2 (±6.4)
Total protein (g/24h urine)	118 (92.2)	1.3 [0.5-2.7][a]	40 (52.6)	1.9 [0.9-4.1][a]	49 (50.0)	1.0 [0.4-2.6][a]
Total protein (g/l urine)	118 (92.2)	0.7 [0.3-1.4][a]	57 (75.0)	1.4 [0.5-2.4][a]	73 (74.5)	0.6 [0.3-1.1][a]
Proteinuria (>0.5 g/24h or L urine)	84 (65.6)		57 (60.5)		48 (49.0)	
Phosphate (mmol/l)	125 (97.7)	1.55 (±0.39)	71 (93.4)	1.47 (±0.42)	97 (99.0)	1.39 (±0.36)
Elevated phosphate (>1.49 mmol/l)	57 (44.5)	1.86 (±0.36)	24 (31.6)	1.92 (±0.39)	30 (30.6)	1.79 (±0.34)
Calcium (mmol/l)	126 (98.4)	2.31 (±0.16)	74 (97.4)	2.27 (±0.19)	98 (100)	2.32 (±0.14)
Elevated Calcium (>2.54 mmol/l)	13 (10.2)	2.65 (±0.07)	5 (6.6)	2.63 (±0.08)	5 (5.1)	2.66 (±0.11)
Haemoglobin level (mmol/l)	127 (99.2)	7.0 (±0.8)	76 (100)	7.0 (±1.2)	98 (100)	7.0 (±0.9)
Low haemoglobin level (<7.5 mmol/l)	88 (68.8)	6.6 (±0.5)	54 (71.1)	6.4 (±0.8)	68 (69.4)	6.6 (±0.7)

CKD: chronic kidney disease; SD: standard deviation; eGFR: estimated glomerular filtration rate; MDRD: Modification of Diet in Renal Disease; SBP: systolic blood pressure; DBP: diastolic blood pressure.

Clinic A and B: university nephrology outpatient clinics; clinic C: non-university nephrology outpatient clinic.

[a] Median with interquartile range.
