# Supplementary material for: Prescribing quality in secondary care patients with different stages of chronic kidney disease: a retrospective study in the Netherlands
Source: BMJ Open. 2019 Jul 19;9(7):e025784. doi: 10.1136/bmjopen-2018-025784 (PMC6661701; doi:10.1136/bmjopen-2018-025784)
Supplement: Supplementary Table 3 [file bmjopen-2018-025784supp003.pdf]

S3 Table. Differences between nephrology outpatient clinics per indicator stratified for chronic kidney disease stage.

Indicator 1 - Antihypertensive use

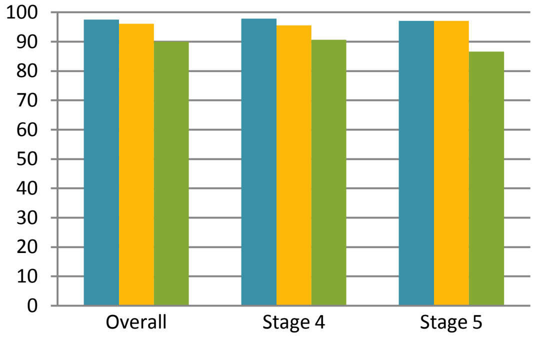

	Overall	Stage 4	Stage 5
Clinic A	97.6	97.9	97.1
Clinic B	96.1	95.6	97.1
Clinic C	90.0	90.6	86.7

Overall	*p-value χ^2*
Clinic A vs. Clinic B	0.316
Clinic A vs. Clinic C	**0.003**
Clinic B vs. Clinic C	0.095
CKD stage 4	
Clinic A vs. Clinic B	0.406†
Clinic A vs. Clinic C	**0.011**
Clinic B vs. Clinic C	0.196
CKD stage 5	
Clinic A vs. Clinic B	1.000†
Clinic A vs. Clinic C	0.103†
Clinic B vs. Clinic C	0.306†

Indicator 2 - RAASi with proteinuria

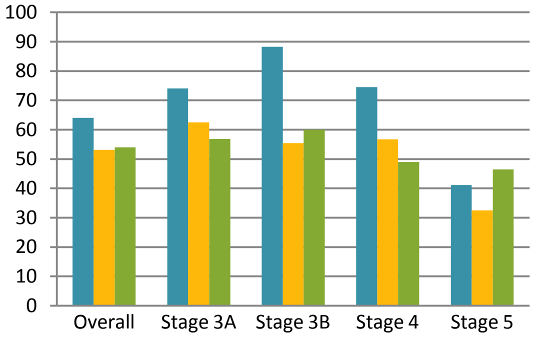

	Overall	Stage 3A	Stage 3B	Stage 4	Stage 5
Clinic A	64.0	74.1	88.2	74.5	41.1
Clinic B	53.1	62.5	55.4	56.7	32.5
Clinic C	54.0	56.9	60.0	49.0	46.4

Overall	*p-value χ^2*
Clinic A vs. Clinic B	0.030
Clinic A vs. Clinic C	**0.003**
Clinic B vs. Clinic C	0.503
CKD stage 3a	
Clinic A vs. Clinic B	0.190
Clinic A vs. Clinic C	0.095
Clinic B vs. Clinic C	0.675
CKD stage 3b	
Clinic A vs. Clinic B	**0.002**
Clinic A vs. Clinic C	**0.001**
Clinic B vs. Clinic C	0.819
CKD stage 4	
Clinic A vs. Clinic B	0.089
Clinic A vs. Clinic C	**0.001**
Clinic B vs. Clinic C	0.117
CKD stage 5	
Clinic A vs. Clinic B	0.209
Clinic A vs. Clinic C	0.243
Clinic B vs. Clinic C	0.923

Indicator 4 - RAASi and diuretics

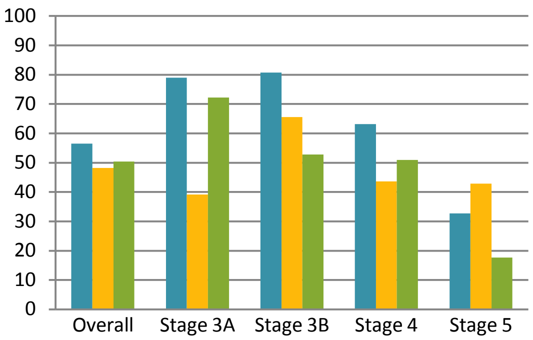

	Overall	Stage 3A	Stage 3B	Stage 4	Stage 5
Clinic A	56.5	78.9	80.8	63.2	32.7
Clinic B	48.2	39.1	65.5	43.6	42.9
Clinic C	50.4	72.2	52.8	50.9	17.6

Overall	*p-value* χ^2
Clinic A vs. Clinic B	0.184
Clinic A vs. Clinic C	0.081
Clinic B vs. Clinic C	0.786
CKD stage 3a	
Clinic A vs. Clinic B	**0.014**
Clinic A vs. Clinic C	1.000
Clinic B vs. Clinic C	**0.014**
CKD stage 3b	
Clinic A vs. Clinic B	0.068
Clinic A vs. Clinic C	**0.002**
Clinic B vs. Clinic C	0.235
CKD stage 4	
Clinic A vs. Clinic B	0.245
Clinic A vs. Clinic C	0.337
Clinic B vs. Clinic C	0.766
CKD stage 5	
Clinic A vs. Clinic B	0.586
Clinic A vs. Clinic C	0.033
Clinic B vs. Clinic C	0.021

Indicator 6 - Statin use

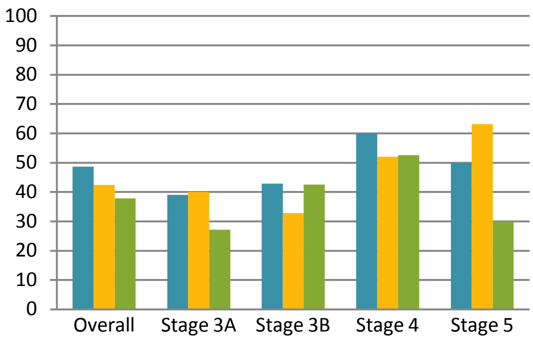

	Overall	Stage 3A	Stage 3B	Stage 4	Stage 5
Clinic A	48.7	39.0	42.9	60.0	50.0
Clinic B	42.4	40.0	32.9	52.0	63.2
Clinic C	37.9	27.2	42.5	52.6	30.0

Overall	*p-value* χ^2
Clinic A vs. Clinic B	0.205
Clinic A vs. Clinic C	**0.015**
Clinic B vs. Clinic C	0.277
CKD stage 3a	
Clinic A vs. Clinic B	0.916
Clinic A vs. Clinic C	0.140
Clinic B vs. Clinic C	0.042
CKD stage 3b	
Clinic A vs. Clinic B	0.266
Clinic A vs. Clinic C	0.966
Clinic B vs. Clinic C	0.189
CKD stage 4	
Clinic A vs. Clinic B	0.409
Clinic A vs. Clinic C	0.395
Clinic B vs. Clinic C	0.950
CKD stage 5	
Clinic A vs. Clinic B	0.340
Clinic A vs. Clinic C	0.309[†]
Clinic B vs. Clinic C	0.128[†]

Indicator 7 - Phosphate binder use

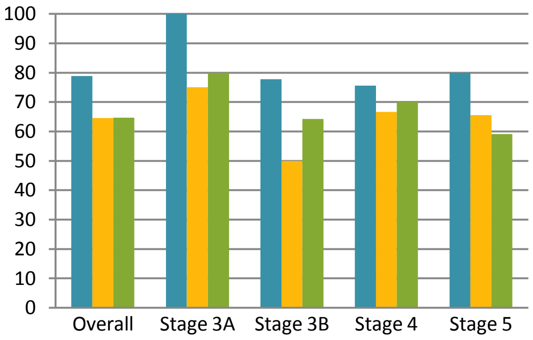

	Overall	Stage 3A	Stage 3B	Stage 4	Stage 5
Clinic A	78.9	100.0	77.8	75.6	80.0
Clinic B	64.6	75.0	50.0	66.7	65.5
Clinic C	64.7	80.0	64.3	70.0	59.1

Overall	*p-value* χ^2
Clinic A vs. Clinic B	**0.016**

Indicator 10 - Dual RAAS blockade

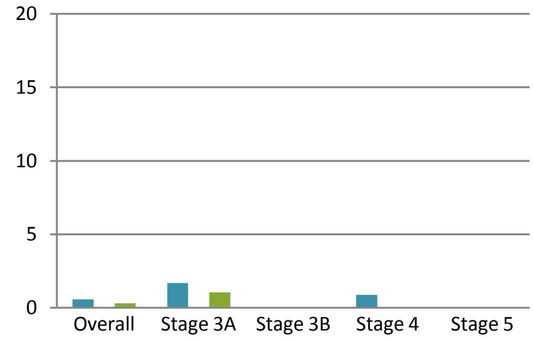

	Overall	Stage 3A	Stage 3B	Stage 4	Stage 5
Clinic A	0.6	1.7	0.0	0.9	0.0
Clinic B	0.0	0.0	0.0	0.0	0.0
Clinic C	0.3	1.0	0.0	0.0	0.0

Overall	*p-value* χ^2
Clinic A vs. Clinic B	0.218[†]

Clinic A vs. Clinic C	0.030
Clinic B vs. Clinic C	0.849
CKD stage 3a	
Clinic A vs. Clinic B	1.000†
Clinic A vs. Clinic C	1.000†
Clinic B vs. Clinic C	1.000†
CKD stage 3b	
Clinic A vs. Clinic B	0.650†
Clinic A vs. Clinic C	1.000†
Clinic B vs. Clinic C	0.692†
CKD stage 4	
Clinic A vs. Clinic B	0.191
Clinic A vs. Clinic C	0.548
Clinic B vs. Clinic C	0.620
CKD stage 5	
Clinic A vs. Clinic B	0.067
Clinic A vs. Clinic C	**0.013**
Clinic B vs. Clinic C	0.575

Clinic A vs. Clinic C	0.612†
Clinic B vs. Clinic C	0.530†
CKD stage 3a	
Clinic A vs. Clinic B	0.323†
Clinic A vs. Clinic C	0.558†
Clinic B vs. Clinic C	0.521†
CKD stage 3b	
Clinic A vs. Clinic B	No prescriptions
Clinic A vs. Clinic C	No prescriptions
Clinic B vs. Clinic C	No prescriptions
CKD stage 4	
Clinic A vs. Clinic B	1.000†
Clinic A vs. Clinic C	0.391†
Clinic B vs. Clinic C	No prescriptions
CKD stage 5	
Clinic A vs. Clinic B	No prescriptions
Clinic A vs. Clinic C	No prescriptions
Clinic B vs. Clinic C	No prescriptions

Indicator 11 - Vitamin D use

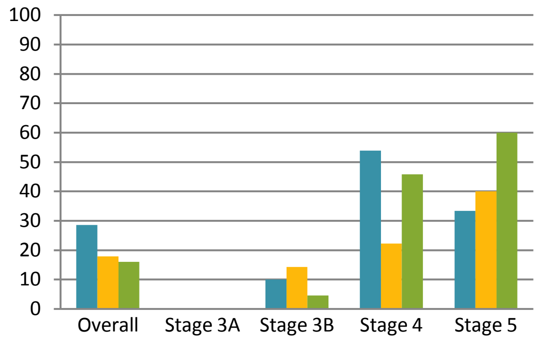

	Overall	Stage 3A	Stage 3B	Stage 4	Stage 5
Clinic A	28.6	0.0	10.0	53.8	33.3
Clinic B	17.9	0.0	14.3	22.2	40.0
Clinic C	16.0	0.0	4.5	45.8	60.0

Overall	*p-value* χ^2
Clinic A vs. Clinic B	0.321
Clinic A vs. Clinic C	0.105
Clinic B vs. Clinic C	0.779†
CKD stage 3a	
Clinic A vs. Clinic B	No prescriptions
Clinic A vs. Clinic C	No prescriptions
Clinic B vs. Clinic C	No prescriptions
CKD stage 3b	
Clinic A vs. Clinic B	1.000†
Clinic A vs. Clinic C	0.466†
Clinic B vs. Clinic C	0.364†
CKD stage 4	
Clinic A vs. Clinic B	0.203†
Clinic A vs. Clinic C	0.642
Clinic B vs. Clinic C	0.263†
CKD stage 5	
Clinic A vs. Clinic B	1.000†
Clinic A vs. Clinic C	0.567†

Indicator 12 - ESA use

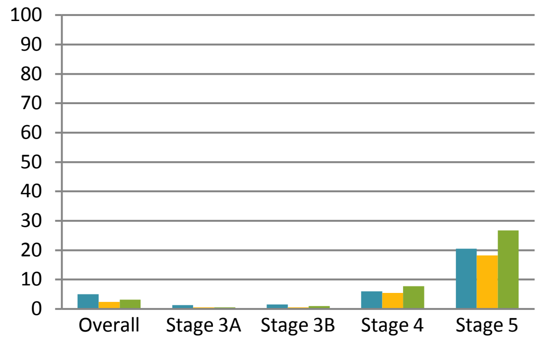

	Overall	Stage 3A	Stage 3B	Stage 4	Stage 5
Clinic A	5.0	1.3	1.6	6.0	20.5
Clinic B	2.3	0.5	0.5	5.5	18.2
Clinic C	3.1	0.5	1.0	7.8	26.7

Overall	*p-value* χ^2
Clinic A vs. Clinic B	0.034
Clinic A vs. Clinic C	0.100
Clinic B vs. Clinic C	0.375
CKD stage 3a	
Clinic A vs. Clinic B	0.469†
Clinic A vs. Clinic C	0.417†
Clinic B vs. Clinic C	1.000†
CKD stage 3b	
Clinic A vs. Clinic B	0.562†
Clinic A vs. Clinic C	0.637†
Clinic B vs. Clinic C	0.678†
CKD stage 4	
Clinic A vs. Clinic B	0.864
Clinic A vs. Clinic C	0.558
Clinic B vs. Clinic C	0.398
CKD stage 5	
Clinic A vs. Clinic B	1.000†
Clinic A vs. Clinic C	0.548

Clinic B vs. Clinic C	1.000†

Clinic B vs. Clinic C	0.473

Indicator 13 - NSAIDs use

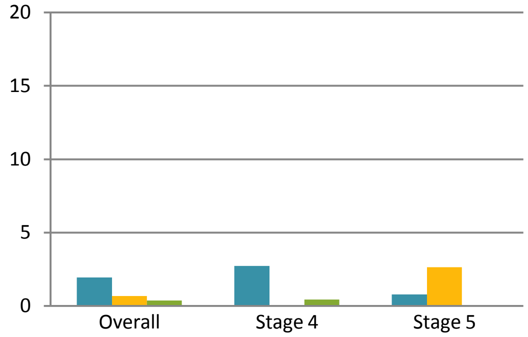

	Overall	Stage 4	Stage 5
Clinic A	1.9	2.7	0.8
Clinic B	0.7	0.0	2.6
Clinic C	0.4	0.4	0.0

Overall	*p-value χ²*
Clinic A vs. Clinic B	0.287†
Clinic A vs. Clinic C	0.028†
Clinic B vs. Clinic C	0.612†
CKD stage 4	
Clinic A vs. Clinic B	0.019†
Clinic A vs. Clinic C	0.022†
Clinic B vs. Clinic C	1.000†
CKD stage 5	
Clinic A vs. Clinic B	0.557†
Clinic A vs. Clinic C	1.000†
Clinic B vs. Clinic C	0.189†

Indicator 14 - Metformin use

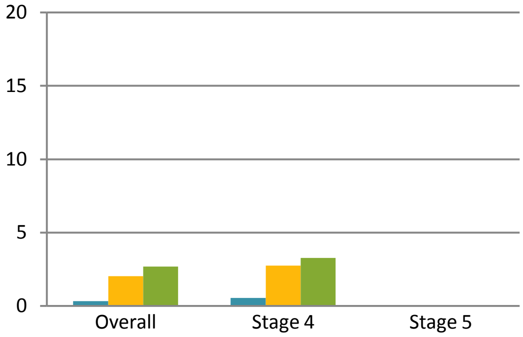

	Overall	Stage 4	Stage 5
Clinic A	0.3	0.5	0.0
Clinic B	2.0	2.7	0.0
Clinic C	2.7	3.3	0.0

Overall	*p-value χ²*
Clinic A vs. Clinic B	0.062
Clinic A vs. Clinic C	**0.013**
Clinic B vs. Clinic C	0.558
CKD stage 4	
Clinic A vs. Clinic B	0.132†
Clinic A vs. Clinic C	0.050†
Clinic B vs. Clinic C	0.714
CKD stage 5	
Clinic A vs. Clinic B	No prescriptions
Clinic A vs. Clinic C	No prescriptions
Clinic B vs. Clinic C	No prescriptions

Indicator 15 - HD digoxin use

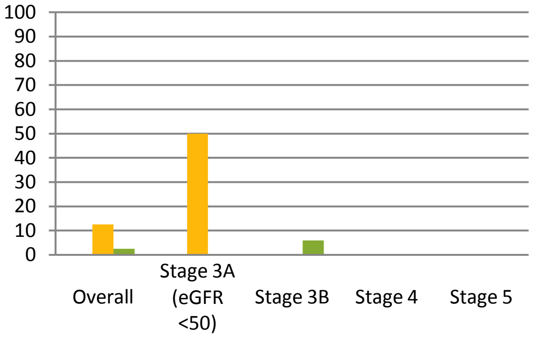

	Overall	Stage 3A (eGFR <50)	Stage 3B	Stage 4	Stage 5
Clinic A	0.0	n.a.	0.0	0.0	0.0
Clinic B	12.5	50.0	0.0	0.0	n.a.
Clinic C	2.4	0.0	5.9	0.0	0.0

Overall	*p-value χ²*
Clinic A vs. Clinic B	1.000†
Clinic A vs. Clinic C	1.000†
Clinic B vs. Clinic C	0.202†
CKD stage 3a	
Clinic A vs. Clinic B	No prescriptions
Clinic A vs. Clinic C	No prescriptions
Clinic B vs. Clinic C	0.333†
CKD stage 3b	

Indicator 16 - NSAIDs, RAAS and diuretics

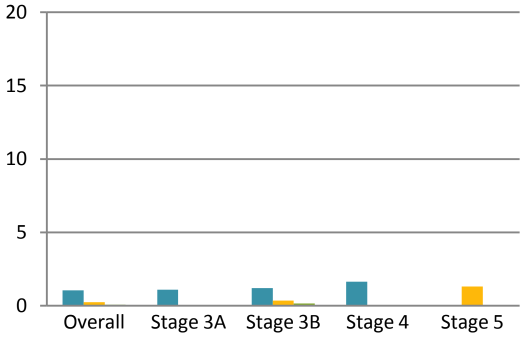

	Overall	Stage 3A	Stage 3B	Stage 4	Stage 5
Clinic A	1.1	1.1	1.2	1.6	0.0
Clinic B	0.2	0.0	0.3	0.0	1.3
Clinic C	0.1	0.0	0.2	0.0	0.0

Overall	*p-value χ²*
Clinic A vs. Clinic B	0.067†
Clinic A vs. Clinic C	**0.001†**
Clinic B vs. Clinic C	0.254†
CKD stage 3a	
Clinic A vs. Clinic B	0.265†
Clinic A vs. Clinic C	0.156†
Clinic B vs. Clinic C	No prescriptions
CKD stage 3b	

Clinic A vs. Clinic B	No prescriptions	Clinic A vs. Clinic B	0.295†
Clinic A vs. Clinic C	1.000†	Clinic A vs. Clinic C	0.104†
Clinic B vs. Clinic C	1.000†	Clinic B vs. Clinic C	0.521†
CKD stage 4		*CKD stage 4*	
Clinic A vs. Clinic B	No prescriptions	Clinic A vs. Clinic B	0.093†
Clinic A vs. Clinic C	No prescriptions	Clinic A vs. Clinic C	0.023†
Clinic B vs. Clinic C	No prescriptions	Clinic B vs. Clinic C	1.000†
CKD stage 5		*CKD stage 5*	
Clinic A vs. Clinic B	No prescriptions	Clinic A vs. Clinic B	0.373†
Clinic A vs. Clinic C	No prescriptions	Clinic A vs. Clinic C	No prescriptions
Clinic B vs. Clinic C	No prescriptions	Clinic B vs. Clinic C	0.437†

CKD: chronic kidney disease; RAAS: renin-angiotensin-aldosterone system; ESA: erythropoiesis-stimulating agent; NSAID: non-steroidal anti-inflammatory drug.

Clinic A and B: university nephrology outpatient clinics; clinic C: non-university nephrology outpatient clinic.

Indicators 3, 5, 8 and 9 were excluded because of insufficient validity.

† Fisher's exact because of <5 expected number of patients in one or more cells.
