# Supplementary material for: Prescribing quality in secondary care patients with different stages of chronic kidney disease: a retrospective study in the Netherlands
Source: BMJ Open. 2019 Jul 19;9(7):e025784. doi: 10.1136/bmjopen-2018-025784 (PMC6661701; doi:10.1136/bmjopen-2018-025784)
Supplement: Author's manuscript [file bmjopen-2018-025784.draft_revisions.pdf]

BMJ Open is committed to open peer review. As part of this commitment we make the peer review history of every article we publish publicly available.

When an article is published we post the peer reviewers' comments and the authors' responses online. We also post the versions of the paper that were used during peer review. These are the versions that the peer review comments apply to.

The versions of the paper that follow are the versions that were submitted during the peer review process. They are not the versions of record or the final published versions. They should not be cited or distributed as the published version of this manuscript.

BMJ Open is an open access journal and the full, final, typeset and author-corrected version of record of the manuscript is available on our site with no access controls, subscription charges or pay-per-view fees (http://bmjopen.bmj.com).

If you have any questions on BMJ Open's open peer review process please email info.bmjopen@bmj.com

BMJ Open

Prescribing quality in secondary care patients with different stages of chronic kidney disease: a retrospective study in the Netherlands

Journal:	*BMJ Open*
Manuscript ID	bmjopen-2018-025784
Article Type:	Research
Date Submitted by the Author:	07-Aug-2018
Complete List of Authors:	Smits, Kirsten; University Medical Center Groningen, Department of Clinical Pharmacy and Pharmacology<br>Sidorenkov, Grigory; University Medical Center Groningen, Department of Clinical Pharmacy and Pharmacology<br>van Ittersum, Frans; VU medisch centrum School of Medical Sciences, Department of Nephrology<br>Waanders, Femke; Isala, Department of Nephrology<br>Bilo, Henk; Isala Clinics, Diabetes Centre<br>Navis, GJ; University Medical Center Groningen, Department of Nephrology<br>Denig, Petra; University Medical Center Groningen, Department of Clinical Pharmacy and Pharmacology
Keywords:	secondary care, chronic kidney disease, prescribing quality, quality indicator, quality assessment

SCHOLARONE™
Manuscripts

PRESCRIBING QUALITY IN SECONDARY CARE PATIENTS WITH DIFFERENT STAGES OF CHRONIC KIDNEY DISEASE: A RETROSPECTIVE STUDY IN THE NETHERLANDS

Kirsten P.J. Smits[1]*, Grigory Sidorenkov[1], Frans J. van Ittersum[2], Femke Waanders[3], Henk J.G. Bilo[4], Gerjan J. Navis[5], Petra Denig[1]

[1] Department of Clinical Pharmacy and Pharmacology, University of Groningen, University Medical Center Groningen, the Netherlands
[2] Department of Nephrology, VU University Medical Center Amsterdam, Amsterdam, the Netherlands
[3] Department of Nephrology, Isala Clinics, Zwolle, the Netherlands
[4] Diabetes Centre, Isala Clinics, Zwolle, the Netherlands
[5] Department of Nephrology, University of Groningen, University Medical Center Groningen, Groningen, the Netherlands

* Corresponding author:
Dr. Kirsten P.J. Smits
Department of Quality and Safety of Oral Healthcare
Radboud university medical center
Philips van Leydenlaan 25, 6525 EX Nijmegen
Email: kirsten.smits@radboudumc.nl

Word count: 2881

For peer review only

ABSTRACT

Objectives: Insight in the prescribing quality for patients with chronic kidney disease (CKD) in secondary care is limited. The aim of this study is to assess the prescribing quality in secondary care patients with CKD stages 3-5, and assess possible differences in quality between CKD stages.

Design: This was a retrospective cohort study.

Setting: Data were collected at two university (n=569 and n=845) and one non-university nephrology outpatient clinic (n=1,718) in the Netherlands.

Participants: Between March 2015 and August 2016, data were collected from patients with stage 3a-5 CKD seen at the clinics. Blood pressure measurements, laboratory measurements and prescription data were extracted from medical records. For each prescribing quality indicator, patients with incomplete data required for calculation were excluded.

Outcome measures: Potentially appropriate prescribing of antihypertensives, renin-angiotensin-aldosterone-system (RAAS) inhibitors, statins, phosphate binders, and potentially inappropriate prescribing according to prevailing guidelines was assessed using prescribing quality indicators. Chi-square or Fisher's Exact tests were used to test for differences in prescribing quality.

Results: RAAS inhibitors alone or in combination with diuretics (57% respectively 52%) and statins (42%) were prescribed less often than phosphate binders (72%) or antihypertensives (94%) when indicated. Active vitamin D was relatively often prescribed when potentially not indicated (19%). Patients with high CKD stages were less likely to receive RAAS inhibitors but more likely to receive statins when indicated than stage 3 CKD patients. They also received more active vitamin D and erythropoietin stimulating agents when potentially not indicated.

For peer review only - http://bmjopen.bmj.com/site/about/guidelines.xhtml

Conclusions: Priority areas for improvement of prescribing in CKD outpatients include potential underprescribing of RAAS inhibitors and statins, and overprescribing of active vitamin D. CKD stage should be taken into account when assessing prescribing quality.

STRENGTHS AND LIMITATIONS OF THIS STUDY

- Quality of prescribing was assessed with validated quality indicators measuring both appropriate and inappropriate prescribing, which take individual patient characteristics into account
- Quality of prescribing was compared between patients with different CKD stages and from different outpatient clinics
- Quality of prescribing was assessed in a cross-sectional manner, which disregards longitudinal aspects of care
- There were differences in patient population and data collection between the three outpatient clinics

Keywords: chronic kidney disease, secondary care, prescribing quality, quality indicator, quality assessment

INTRODUCTION

Assessing quality of care in chronic kidney disease patients (CKD) is important for identifying areas for improvement. Several recent studies have shown that detection of CKD, monitoring of disease progression and metabolic parameters, and achievement of risk factor target levels are suboptimal in CKD care.[1-4] Three of these studies showed that prescribing of selected medication treatment may also be suboptimal, for example, showing potential underprescribing of renin-angiotensin-aldosterone system (RAAS) inhibitors and statins, and overprescribing of non-steroidal anti-inflammatory drugs (NSAIDs) in primary care patients with CKD. In addition, one study showed an

For peer review only - http://bmjopen.bmj.com/site/about/guidelines.xhtml

For peer review only

increasing trend in prescribing of RAAS inhibitors and statins in secondary CKD patients.[5] Not much is known about differences in prescribing quality in CKD patients between healthcare organizations.

Recently, our research group has developed a set of prescribing quality indicators (PQIs) for assessing the prescribing quality in patients with CKD according to clinical guideline recommendations, which has been validated in a primary care population.[6] The set is intended also for secondary care, and includes several indicators which are specifically relevant for patients with higher CKD stages. The PQIs give new insights in quality of prescribing in CKD patients, since they assess a broad range of prescribing issues at patient level and incorporate patient characteristics that are relevant for treatment decisions. Previously it was found that with increasing CKD stages prescribing of RAAS inhibitors and NSAIDs decreased, while prescribing of phosphate binders, vitamin D and erythropoietin-stimulating-agents (ESA) increased.[1] However, this was based on the number of prescriptions regardless of whether the medication was indicated for the included patients. The aim of this study is to assess prescribing quality in secondary care patients with CKD stage 3a-5 using PQIs that take relevant patient characteristics into account. Differences in prescribing quality between patients with different CKD stages were evaluated. In a secondary analysis, also differences between different outpatient clinics in the Netherlands were explored.

METHODS

This was a retrospective cross-sectional study assessing the prescribing quality between March 2015 and August 2016 in the Netherlands in two university nephrology outpatient clinics A and B, and one non-university nephrology outpatient clinic C. Included were patients with CKD stage 3a-5 based on estimated glomerular filtration rate (eGFR), i.e. stage 3a was defined as an eGFR ≥45 and <60 ml/min/1.73m2, stage 3b as an eGFR ≥30 and <45 ml/min/1.73m2, stage 4 as an eGFR ≥15 and <30 ml/min/1.73m2, and stage 5 as an eGFR <15 ml/min/1.73m2.[7] Patients who received dialysis or renal transplantation were excluded from the study.

For peer review only - http://bmjopen.bmj.com/site/about/guidelines.xhtml

The medical ethical committee of the University Medical Center Groningen ascertained that this study using anonymized medical record data does not fall under the Medical Research Involving Human Subjects Act.

Clinics

Clinic A and clinic B are academic hospitals, which provided data from their general nephrology outpatient clinic and their predialysis outpatient clinic. Clinic C is a nonacademic, public hospital, which provided data from the general nephrology outpatient clinic. Twelve months of consecutive data were collected from June 1st 2015 to May 31st 2016 for clinic A, from March 1st 2015 to February 29th 2016 for clinic B and September 1st 2015 to August 31st 2016 for clinic C. In all three clinics, the included CKD patients commonly visit the outpatient clinics 2-4 times per year depending on the progression of their disease. At these visits, all medication can be reviewed and changed based on a medication list which is available for the nephrologist. In all clinics, the medication included in this study is usually prescribed by the nephrologist or nephrologist in training, although other specialists or the general practitioner may also prescribe medication during the year.

Prescribing quality

A previously developed set of patient-oriented PQIs was used for the assessment of prescribing quality.[6] This set was based on clinical guideline recommendations. The PQIs intend to provide insight in prescribing of antihypertensives, RAAS inhibitors, statins and phosphate binders when recommended (potentially appropriate prescribing) as well as prescribing of dual RAAS blockade, active vitamin D, ESA, NSAIDs, metformin, and digoxin when considered not needed or unsafe (potentially inappropriate prescribing) (Table 1). To specify the indicators to specific needs of patients, most indicators focus on a subgroup of the population selected based on kidney function, risk factor levels and/or age. Since there will always be individual cases for whom this is not the case, we speak of ‘potentially’ appropriate (or inappropriate) prescribing. Antihypertensives include diuretics, beta blocking agents, calcium channel blockers, agents acting on the RAAS system and

For peer review only - http://bmjopen.bmj.com/site/about/guidelines.xhtml

other antihypertensives such as centrally acting agents. Diuretics include all types of diuretics, e.g. low-ceiling and high-ceiling diuretics and potassium-sparing agents. RAAS inhibitors include angiotensin-converting-enzyme inhibitors and angiotensin-receptor-blockers. In the Netherlands, this medication is reimbursed by the health insurance after the patient's annual deductible excess has been exceeded. This is the same for all three clinics.

Table 1. Indicator definitions for the PQIs

Nr.	Indicator definition
Appropriate prescribing	
**1**	The percentage of patients between 18 and 80 years with CKD stages 4-5 and hypertension[†] that is prescribed antihypertensives unless undesirable because of low diastolic blood pressure (<70 mmHg)
**2**	The percentage of patients between 18 and 80 years with CKD stages 3-5 and proteinuria[‡] that is prescribed an ACE-i or ARB
**3**	The percentage of patients between 18 and 80 years with CKD stages 3-5, microalbuminuria[$] and diabetes[§] that is prescribed an ACE-I or ARB
**4**	The percentage of patients between 18 and 80 years with CKD stages 3-5 and proteinuria[‡] treated with multiple antihypertensives that is prescribed a combination of an ACE-i or ARB and a diuretic
**5**	The percentage of patients between 18 and 80 years with CKD stages 3-5, microalbuminuria[$] and diabetes[§] treated with multiple antihypertensives that is prescribed a combination of an ACE-I or ARB and a diuretic.
**6**	The percentage of patients between 50 and 65 years with CKD stages 3-5 that is prescribed a statin
**7**	The percentage of patients between 18 and 80 years with CKD stages 3-5 and an elevated phosphate level (>1.49 mmol/l) that is prescribed a phosphate binder
**8**	The percentage of patients between 18 and 80 years with CKD stages 3-5 treated with phosphate binders and with an elevated calcium level (>2.54 mmol/l) that is prescribed a non-calcium-containing phosphate binder
**9**	The percentage of patients between 18 and 80 years with CKD stages 3-5 treated with phosphate binders and with a low calcium level (<2.10 mmol/l) that is prescribed a calcium-containing phosphate binder
Inappropriate prescribing	
**10**	The percentage of patients 18 years or older with CKD stages 3-5 treated with RAAS inhibitors that is prescribed at least two RAAS inhibitors simultaneously (dual RAAS blockade)
**11**	The percentage of patients 18 years or older with CKD stages 3-5 and an elevated calcium level (>2.54 mmol/l) that is prescribed active vitamin D
**12**	The percentage of patients 18 years or older with CKD stages 3-5 and a normal haemoglobin level (≥7.5 mmol/l) that is prescribed an ESA
**13**	The percentage of patients 18 years or older with eGFR < 30 ml/min/1.73 m^2 that is prescribed an NSAID
**14**	The percentage of patients 18 years or older with eGFR < 30 ml/min/1.73 m^2 and diabetes[§] that is prescribed metformin
**15**	The percentage of patients 18 years or older with eGFR < 50 ml/min/1.73 m^2 treated with digoxin that is prescribed high dose digoxin (>0.125 mg/day)
**16**	The percentage of patients 18 years or older with CKD stages 3-5 that is prescribed a

For peer review only - http://bmjopen.bmj.com/site/about/guidelines.xhtml

combination of NSAIDs, RAAS inhibitors and diuretics

CKD: chronic kidney disease; ACE-i: angiotensin-converting-enzyme inhibitor; ARB: angiotensin-receptor-blocker; RAAS: renin-angiotensin-aldosterone-system; ESA: erythropoietin-stimulating agent; eGFR: estimated glomerular filtration rate; NSAIDs: non-steroidal anti-inflammatory drugs.

† Hypertension is defined as having a systolic blood pressure > 140 mmHg or being prescribed antihypertensives. $ Micro-albuminuria is defined as albumin/creatinine ratio ≥3.0 mg/mmol and <30 mg/mmol. ‡ Proteinuria is defined as > 0.5 g protein per 24h or l urine or albumin/creatinine ratio ≥30 mg/mmol. § diabetes is defined as either the diagnosis for diabetes or being prescribed with glucose lowering drugs.

Data collection

For each patient with at least one visit to a nephrologist within the study period, blood pressure measurement, laboratory measurements and prescription data of the most recent visit were extracted from the medical records, either by manual (clinic A) or computerized (clinic B and C) extraction routines. Age was determined on the visit day. For some patients, the visit date was unknown, in which case the most recent date of the eGFR assessment was used as a proxy for the visit date. The eGFR was calculated from serum creatinine using the Modification of Diet in Renal Disease (MDRD) formula.[8] If serum creatinine was not available, the reported eGFR calculated with the MDRD formula was used. Proteinuria was defined as more than 0.5 g of protein in 24 hour or per liter urine, depending on availability.

Patient and Public involvement

Patients or public were not directly involved in this study. Patient representatives were, however, involved in the development phase of the prescribing quality indicators applied in this study.

Statistical analysis

Means with standard deviations (SD) are reported for normally distributed continuous variables, medians with interquartile ranges for non-normally distributed variables, and percentages for categorical variables. The PQI scores are presented as percentages with 95% confidence intervals (CI). Chi-square tests or Fisher's Exact tests, in case of cell frequencies below 5, were used to test for

For peer review only - http://bmjopen.bmj.com/site/about/guidelines.xhtml

differences in prescribing quality across different CKD stages and different clinics. P-values <0.05 were considered statistically significant. When comparing individual PQIs between CKD stages or clinics, Bonferroni correction for multiple testing was applied. Analyses were conducted using Stata version 14.2 Special Edition (Stata Corp., College Station, TX).

RESULTS

In total, 3,132 patients with CKD stage 3a-5 were included in this study. Included patients were on average 68 years (SD: 14) old, 56% were males, the median eGFR was 35 ml/min/1.73m2 (interquartile range: 24-46) and 16% had diabetes. Patients with higher CKD stages more often had blood pressure and laboratory measurements (Table 2).

For peer review only

Table 2. Patient characteristics for the whole population and separate per CKD stage

	Overall (n=3,132)		CKD 3a (n=843)		CKD 3b (n=1,125)		CKD 4 (n=862)		CKD 5 (n=302)		Chi^2
	**N (%)**	**Mean (±SD)**	**N (%)**	**Mean (±SD)**	**N (%)**	**Mean (±SD)**	**N (%)**	**Mean (±SD)**	**N (%)**	**Mean (±SD)**	**p-value**
Age (years)		67.7 (±14.1)		63.1 (±14.2)		68.9 (±13.2)		70.2 (±13.8)		69.2 (±14.8)	
< 50 years	363 (11.6)		151 (17.9)		113 (10.0)		70 (8.1)		29 (9.6)		
50-80 years	2,149 (68.6)		604 (71.7)		793 (70.5)		556 (64.5)		196 (64.9)		
>= 80 years	620 (19.8)		88 (10.4)		219 (19.5)		236 (27.4)		77 (25.5)		
Gender (males)	1,738 (55.5)		456 (54.1)		614 (54.6)		488 (56.6)		180 (59.6)		0.314
Diabetes (yes)	485 (15.5)		96 (11.4)		166 (14.8)		165 (19.1)		58 (19.2)		<0.001
eGFR (MDRD) (ml/min/1.73m^2	3,132 (100)	35 [24-46][a]	843 (100)	52.2 (±4.3)	1,125 (100)	37.3 (±4.2)	862 (100)	23.1 (±4.3)	302 (100)	11.1 (±2.6)	
SBP (mmHg)	2,511 (80.2)	132.0 (±18.8)	601 (71.3)	129.4 (±17.3)	910 (80.9)	130.7 (±18.6)	735 (85.3)	133.3 (±19.4)	265 (87.7)	139.1 (±19.2)	<0.001
Elevated SBP (>140 mmHg)	700 (22.3)	155.3 (±12.4)	119 (14.1)	155.3 (±12.3)	239 (21.2)	154.3 (±12.5)	233 (27.0)	155.4 (±12.2)	109 (36.1)	157.1 (±12.6)	
DBP (mmHg)	2,511 (80.2)	75.1 (±11.2)	601 (71.3)	76.7 (±10.6)	910 (80.9)	74.9 (±11.3)	735 (85.3)	74.6 (±11.2)	265 (87.7)	74.0 (±12.1)	<0.001
Low DBP (<70 mmHg)	704 (22.5)	61.6 (±5.8)	130 (15.4)	62.1 (±5.5)	282 (25.1)	62.1 (±5.4)	214 (24.8)	61.3 (±6.1)	78 (25.8)	59.4 (±6.5)	
Total protein (g/24h urine)	1,314 (42.0)	0.4 [0.1-1.3][a]	278 (33.0)	0.3 [0.1-0.8][a]	426 (37.9)	0.2 [0.1-0.8][a]	403 (46.8)	0.4 [0.2-1.3][a]	207 (68.5)	1.3 [0.5-2.8][a]	<0.001
Total protein (g/L urine)	2,328 (74.3)	0.2 [0.1-0.6][a]	552 (65.5)	0.1 [0.1-0.3][a]	841 (74.8)	0.2 [0.1-0.4][a]	687 (79.7)	0.3 [0.1-0.6][a]	248 (82.1)	0.8 [0.3-1.7][a]	<0.001
Proteinuria (>0.5 g/24h or L urine)	810 (25.9)		140 (16.6)		224 (19.9)		268 (31.1)		178 (58.9)		
Phosphate (mmol/l)	2,606 (83.2)	1.08 (±0.29)	539 (63.9)	0.96 (±0.20)	959 (85.2)	1.00 (±0.20)	815 (94.5)	1.10 (±0.25)	293 (97.0)	1.48 (±0.39)	<0.001
Elevated phosphate (>1.49 mmol/l)	172 (5.5)	1.80 (±0.34)	3 (0.4)	1.69 (±0.22)	17 (1.5)	1.65 (±0.20)	41 (4.8)	1.72 (±0.32)	111 (36.8)	1.85 (±0.36)	
Calcium (mmol/l)	2,734 (87.3)	2.36 (±0.14)	616 (73.1)	2.38 (±0.11)	998 (88.7)	2.37 (±0.13)	822 (95.4)	2.35 (±0.15)	298 (98.7)	2.30 (±0.16)	<0.001
Elevated Calcium (>2.54 mmol/l)	163 (5.2)	2.62 (±0.08)	40 (4.7)	2.61 (±0.08)	61 (5.4)	2.62 (±0.09)	46 (5.3)	2.62 (±0.06)	16 (5.3)	2.64 (±0.08)	
Haemoglobin (mmol/l)	3,024 (96.6)	8.0 (±1.1)	771 (91.5)	8.5 (±1.1)	1,095 (97.3)	8.2 (±1.0)	857 (99.4)	7.7 (±1.1)	301 (99.7)	7.0 (±0.9)	<0.001
Low haemoglobin level (<7.5 mmol/l)	933 (29.8)	6.7 (±0.6)	113 (13.4)	6.8 (±0.6)	264 (23.5)	6.8 (±0.5)	346 (40.1)	6.7 (±0.6)	210 (69.5)	6.6 (±0.7)	
Clinic											<0.001
A	569 (18.2)		92 (10.9)		166 (14.8)		183 (21.2)		128 (42.4)		
B	845 (27.0)		255 (30.3)		295 (26.2)		219 (25.4)		76 (25.2)		
C	1,718 (54.9)		496 (58.8)		664 (59.0)		460 (53.4)		98 (32.5)		

CKD: chronic kidney disease; eGFR: estimated glomerular filtration rate; MDRD: Modification of Diet in Renal Disease; SBP: systolic blood pressure; DBP: diastolic blood pressure. Clinic A and B: university nephrology outpatient clinics; clinic C: non-university nephrology outpatient clinic.

[a] Median with interquartile range.

For peer review only

Overall prescribing quality

Two PQIs focusing on prescribing of RAAS inhibitors in patients with micro-albuminuria and diabetes were not operational valid in this population because the limited availability of albumin/creatinin ratios (indicators 3 and 5). Furthermore, two PQIs focusing on prescribing of non-calcium-containing and calcium-containing phosphate binders in patients with respectively high and low calcium levels were not operational valid because of the low inclusion of eligible patients (indicators 8 and 9). These four PQIs were excluded from further analysis.

Potentially appropriate prescribing rates varied from 94% of patients receiving antihypertensives, 57% and 52% receiving RAAS inhibitors alone or in combination with a diuretic, 42% receiving statins, and 72% receiving phosphate binders when indicated according to the guideline (Fig 1). Potentially inappropriate prescribing rates varied from 19% of patients receiving active vitamin D, 3% receiving ESA, 1% receiving NSAIDs, 3% receiving metformin and 4% receiving high-dose digoxin when this was possibly not needed or unsafe.

Prescribing quality across chronic kidney disease stages

Potential appropriate prescribing of RAAS inhibitors alone occurred significantly less in patients with CKD stage 5 compared to all other stages, which was also true for the combination of RAAS inhibitors and diuretics (Fig 2). Patients with stage 3a were less likely to receive recommended treatment with statins than patients with stage 4 or 5. Similarly, patients with stage 3b were less likely to receive statins compared to patients with stage 4. Potential inappropriate prescribing of active vitamin D in patients with elevated calcium occurred significantly less in patients with stages 3a and 3b compared to patients with stages 4 and 5. This was also the case for potentially inappropriate prescribing of ESA. Finally, potentially inappropriate prescribing of metformin in patients with an eGFR < 30 ml/min/1.73 m2 was significantly lower for stage 5 as compared to stage 4.

Prescribing quality across nephrology outpatient clinics

For peer review only - http://bmjopen.bmj.com/site/about/guidelines.xhtml

Patients visiting the university outpatient clinics A and B were on average younger (63 years (SD: 15) and 65 years (SD: 15)) compared to those visiting the non-university outpatient clinic C (71 years (SD: 13)). Furthermore, patients visiting clinic A more often had CKD stage 4 or 5 compared to patients from clinics B and C (Table 2). The diabetes prevalence was higher at clinic A (26%) compared to clinic B (19%) and clinic C (10%) (Supplementary table 1).

Significant differences were seen between clinic A and clinic C in potentially appropriate prescribing of antihypertensives, RAAS inhibitors alone, statins, and in potentially inappropriate prescribing of metformin as well as the combination of NSAIDs, RAAS inhibitors and diuretics (Fig 3). Furthermore, significantly more potentially appropriate prescribing of phosphate binders was seen in clinic A as compared to clinic B.

In the analyses per CKD stage (Supplementary tables 2 and 3), similar differences were found between the clinics. In patients with stage 3a CKD, potentially appropriate prescribing of RAAS inhibitors combined with diuretics occurred the least in clinic B. In patients with stage 3b CKD, potential appropriate prescribing of RAAS inhibitors alone or combined with diuretics occurred the most in clinic A. Patients with stage 4 CKD were significantly more likely in clinic A as compared to clinic C to receive antihypertensives and RAAS inhibitors alone. Also, patients with CKD stage 5 were more likely in clinic A as compared to clinic C to receive phosphate binders when indicated.

DISCUSSION

This is a first study to assess the prescribing quality in secondary care CKD patients using a broad set of patient-oriented PQIs and comparing patients with different CKD stages and from different outpatient clinics. The results show that the prescribing quality varied between therapeutic areas. RAAS inhibitors and statins were prescribed in less than 60% of the patients for whom this is potentially indicated, whereas potentially appropriate prescribing rates for antihypertensives and phosphate binders were much higher. Potentially inappropriate prescribing occurred most regarding active vitamin D. The prescribing quality also varied across different CKD stages, with decreasing

For peer review only - http://bmjopen.bmj.com/site/about/guidelines.xhtml

potentially appropriate prescribing of RAAS inhibitors, increasing potentially appropriate prescribing of statins, and increasing potential inappropriate prescribing of active vitamin D and ESA with higher CKD stages. Finally, significant differences were observed in prescribing between the different outpatient clinics, also after stratification for CKD stage.

Previous studies looking at the overall volume of prescribing suggested that there was underprescribing of RAAS inhibitors and statins[1, 5] and overprescribing of NSAIDs[1] in patients with CKD. In addition, it was shown that patients with higher CKD stages receive more treatment with antihypertensives, phosphate binders, vitamin D and ESA than patients with lower CKD stages.[1, 9] On the other hand, RAAS inhibitors and NSAIDs were less prescribed with increasing CKD stages.[1] These studies, however, did not take the specific indications for treatment into account. Our study using validated PQIs, which assess prescribing in patients for whom this is indicated, confirmed that underprescribing of RAAS inhibitors and statins are areas for possible improvement in CKD care. This was also found in a recent study among patients with stage 3-4 CKD in Canada.[3] In some patients with CKD stage 5 who are in preparation of dialysis or transplantation, RAAS inhibitors may be deliberately stopped to retain the residual kidney function.[10] Lower statin prescribing rates in patients with lower CKD stages suggests that prescribers may be less aware or convinced of the need to prescribe statins in these patients. A similar pattern of less statin prescribing in patients with a higher eGFR compared to lower eGFR was observed for the elderly primary care patients in Canada.[11] Regarding potentially inappropriate prescribing, the Canadian primary care study observed a relatively high prescribing rate of NSAIDs (16%) and low prescribing rate of dual RAAS blockade (3%). Our study showed that potentially unsafe prescribing of both NSAIDs and dual RAAS blockade was uncommon in secondary care patients managed in The Netherlands.

Although the applied PQIs are patient-oriented, taking relevant patient characteristics into account, they reflect general guideline recommendations, and therefore a perfect score is never pursued. There can be valid reasons to refrain from prescribing according to guideline recommendations in

For peer review only - http://bmjopen.bmj.com/site/about/guidelines.xhtml

certain patients. Valid reasons include lack of response to certain drugs, drug intolerances or patient preferences for or against certain treatment. However, guideline recommendations should guide clinical practice and are therefore useful to provide insight and monitor the quality of care. It has been argued that patient case-mix including difference in aspects, such as age or comorbidities, may explain differences in quality scores.[12] However, these may not necessarily be valid reasons for not complying with guideline recommendations. When developing the PQIs, such differences are to some extent included in the indicator definitions (e.g. age limits), thereby ensuring that the treatments are in general either recommended or inappropriate in the patients included in the indicator. Furthermore, a recent review showed that unjustified case-mix corrections can mask actual differences in quality of care.[13] Therefore, no case-mix adjustment was made when applying the PQIs.

This study assessed the prescribing quality in a cross-sectional manner, since the PQIs were defined as cross-sectional measures. This may lead to including patients who reached abnormal risk factor levels for the first time. In some cases, the healthcare provider may decide to postpone the start of treatment until a next measurement to make sure that the abnormal risk factor level persists. This also holds for discontinuation of active vitamin D in patients with elevated calcium levels and ESA in patients with normal haemoglobin levels. Furthermore, it is possible that the laboratory results became available after the visit. Therefore, the healthcare provider may not have been aware at the time of the visit that they should start or discontinue medication. In the diabetes field, it has been proposed that indicators using multiple time points may give a more accurate assessment of the prescribing quality.[14, 15] Such indicators assess whether the healthcare providers start or intensify treatment when patients do not return to normal risk factor levels.

Differences between clinics may in part be due to differences in the underlying patient population, such as the age and comorbidity. All PQIs focusing on recommended treatment with antihypertensives, RAAS inhibitors, statins and phosphate binders, however, have an age limit which

For peer review only - http://bmjopen.bmj.com/site/about/guidelines.xhtml

excludes the older, more frail patients. Diabetes prevalence was higher in clinic A, which may have affected prescribing. Other studies indicate that CKD patients with diabetes receive better quality of care in general,[16] and have higher prescription rates of RAAS inhibitors and statins.[3] There were also differences in data collection methods between the clinics. Although we were able to extract and combine the data in order to make comparisons possible, there were some differences in availability of measurement values. Furthermore, data from the blood pressure and laboratory measurements were sometimes missing (table 2), with the highest rate of missingness for clinic B (Supplementary table 2). This could have influenced the outcome of the PQIs, since patients with unknown values were not included in the PQIs. We can only speculate why these values were missing, and how this may have influenced the assessments. It could be that the blood pressure and laboratory measurements were not performed, not recorded or lost during data extraction.

In conclusion, using a novel set of PQIs assessing prescribing quality at patient level we successfully identified several areas for improvement. This included potential underprescribing of RAAS inhibitors and statins and overprescribing of active vitamin D in secondary care patients with CKD stages 3-5. We observed differences in prescribing quality between the CKD stages and between the outpatient clinics. We conclude that monitoring of the prescribing quality with PQIs is needed in secondary care to identify priority areas for improvement, and that stratification on CKD stage can be useful in quality improvement initiatives.

ACKNOWLEDGEMENTS

We would like to thank the following people for the data collection: Judith Bos (Clinic A), Thijme Last (Clinic B) and Bert Dikkeschei and Erwin Booij (Clinic C).

COMPETING INTERESTS

None declared.

AUTHOR CONTRIBUTIONS

For peer review only - http://bmjopen.bmj.com/site/about/guidelines.xhtml

Research idea and study design: KPJ, GS, HJG, GJN, PD; data acquisition: KPJ, FvI, HJG, PD; data analysis and interpretation: KPJ GS, HJG, GJN, PD; supervision or mentorship: GS, HJG, GJN, PD. Each author contributed important intellectual content during manuscript drafting or revisions. All authors approved the manuscript and this submission.

FUNDING

This work was supported by ZonMW, the Netherlands Organisation for Health Research and Development, grant number 836021013. The funder had no role in study design, data collection and analysis, decision to publish, or preparation of the manuscript.

DATA SHARING STATEMENT

Clinical data as collected from the outpatient clinics cannot be made publicly available according to Dutch privacy regulations. Datasets are only available for data integrity auditing procedures.

REFERENCES

1. Arora P, Elkin PL, Eberle J, Bono JJ, Argauer L, Murray BM, et al. An observational study of the quality of care for chronic kidney disease: a Buffalo and Albany, New York metropolitan area study. BMC Nephrol. 2015;16:199.
2. Jolly SE, Navaneethan SD, Schold JD, Arrigain S, Sharp JW, Jain AK, et al. Chronic kidney disease in an electronic health record problem list: quality of care, ESRD, and mortality. Am J Nephrol. 2014;39(4):288-96.
3. Manns L, Scott-Douglas N, Tonelli M, Weaver R, Tam-Tham H, Chong C, et al. A Population-Based Analysis of Quality Indicators in CKD. Clin J Am Soc Nephrol. 2017;12(5):727-33.
4. Van Gelder VA, Scherpbier-De Haan ND, De Grauw WJ, Vervoort GM, Van Weel C, Biermans MC, et al. Quality of chronic kidney disease management in primary care: a retrospective study. Scand J Prim Health Care. 2016;34(1):73-80.
5. Ang GY, Heng BH, Liew AS, Chong PN. Quality of care of patients with chronic kidney disease in national healthcare group polyclinics from 2007 to 2011. Ann Acad Med Singapore. 2013;42(12):632-9.
6. Smits KP, Sidorenkov G, Bilo HJ, Bouma M, van Ittersum FJ, Voorham J, et al. Development and initial validation of prescribing quality indicators for patients with chronic kidney disease. Nephrol Dial Transplant. 2016;31(11):1876-86.
7. Kidney Disease: Improving Global Outcomes (KDIGO) CKD Work Group. KDIGO 2012 Clinical Practice Guideline for the Evaluation and Management of Chronic Kidney Disease. Kidney Inter, Suppl. 2013;3(1):S1-150.
8. Levey AS, Bosch JP, Lewis JB, Greene T, Rogers N, Roth D. A more accurate method to estimate glomerular filtration rate from serum creatinine: a new prediction equation. Modification of Diet in Renal Disease Study Group. Ann Intern Med. 1999;130(6):461-70.

For peer review only - http://bmjopen.bmj.com/site/about/guidelines.xhtml

9. Jameson K, Jick S, Hagberg KW, Ambegaonkar B, Giles A, O'Donoghue D. Prevalence and management of chronic kidney disease in primary care patients in the UK. Int J Clin Pract. 2014;68(9):1110-21.
10. Ahmed AK, Kamath NS, El Kossi M, El Nahas AM. The impact of stopping inhibitors of the renin-angiotensin system in patients with advanced chronic kidney disease. Nephrol Dial Transplant. 2010;25(12):3977-82.
11. Nash DM, Brimble S, Markle-Reid M, McArthur E, Tu K, Nesrallah GE, et al. Quality of Care for Patients With Chronic Kidney Disease in the Primary Care Setting: A Retrospective Cohort Study From Ontario, Canada. Can J Kidney Health Dis. 2017;4:1-14.
12. Lilford R, Mohammed MA, Spiegelhalter D, Thomson R. Use and misuse of process and outcome data in managing performance of acute medical care: avoiding institutional stigma. Lancet. 2004;363(9415):1147-54.
13. Calsbeek H, Markhorst JG, Voerman GE, Braspenning JC. Case-mix adjustment for diabetes indicators: a systematic review. Am J Manag Care. 2016;22(2):e45-52.
14. Voorham J, Denig P, Wolffenbuttel BH, Haaijer-Ruskamp FM. Cross-sectional versus sequential quality indicators of risk factor management in patients with type 2 diabetes. Med Care. 2008;46(2):133-41.
15. Aron DC. Quality indicators and performance measures in diabetes care. Curr Diab Rep. 2014;14(3):472.
16. van Dipten C, van Berkel S, van Gelder VA, Wetzels JFM, Akkermans RP, de Grauw WJC, et al. Adherence to chronic kidney disease guidelines in primary care patients is associated with comorbidity. Fam Pract. 2017;34(4):459-66.

For peer review only

For peer review only - http://bmjopen.bmj.com/site/about/guidelines.xhtml

FIGURE LEGENDS

Fig 1. Overall prescribing quality assessed with 5 PQIs for appropriate prescribing (Ind 1-7) and 7 PQIs for potential inappropriate prescribing (Ind 10-16). Ind 1: Patients with hypertension prescribed antihypertensives; Ind 2: Patients with albuminuria prescribed renin-angiotensin-aldosterone system (RAAS) inhibitors; Ind 4: Patients on multiple antihypertensives prescribed a combination of RAAS inhibitors and diuretics; Ind 6: Patients aged 50 to 65 years prescribed statins; Ind 7: Patients with high phosphate levels prescribed phosphate binders; Ind 10: Patients prescribed dual RAAS blockade; Ind 11: Patients with high calcium levels prescribed active vitamin D; Ind 12: Patients with normal haemoglobin levels prescribed erythropoietin stimulating agents; Ind 13: Patients with an estimated glomerular filtration rate (eGFR) lower than 30 ml/min/1.73m^2 prescribed high dose non-steroidal anti-inflammatory drugs (NSAIDs); Ind 14: Patients with diabetes and an eGFR lower than 30 ml/min/1.73m^2 prescribed metformin; Ind 15: Patients with an eGFR lower than 50 ml/min/1.73m^2 prescribed high dose digoxin; Ind 16: Patients prescribed a combination of NSAIDs, RAAS inhibitors and diuretics. 95% CIs were calculated based on included number of patients in the denominator of each indicator.

Fig 2. Prescribing quality across different CKD stages (3A-5) assessed with 5 PQIs for appropriate prescribing (Ind 1-7) and 7 PQIs for potential inappropriate prescribing (Ind 10-16). Ind 1: Patients with hypertension prescribed antihypertensives; Ind 2: Patients with albuminuria prescribed renin-angiotensin-aldosterone system (RAAS) inhibitors; Ind 4: Patients on multiple antihypertensives prescribed a combination of RAAS inhibitors and diuretics; Ind 6: Patients aged 50 to 65 years prescribed statins; Ind 7: Patients with high phosphate levels prescribed phosphate binders; Ind 10: Patients prescribed dual RAAS blockade; Ind 11: Patients with high calcium levels prescribed active vitamin D; Ind 12: Patients with normal haemoglobin levels prescribed erythropoietin stimulating agents; Ind 13: Patients with an estimated glomerular filtration rate (eGFR) lower than 30 ml/min/1.73m^2 prescribed high dose non-steroidal anti-inflammatory drugs (NSAIDs); Ind 14: Patients with diabetes and an eGFR lower than 30 ml/min/1.73m^2 prescribed metformin; Ind 15: Patients with an eGFR lower than 50 ml/min/1.73m^2 prescribed high dose digoxin; Ind 16: Patients prescribed a combination of NSAIDs, RAAS inhibitors and diuretics. 95% CIs were calculated based on included number of patients in the denominator of each indicator. * Significant difference between 2 or more CKD stages using Chi-square or Fisher's exact test with Bonferroni-correction for multiple testing.

Fig 3: Prescribing quality across different outpatient clinics. Clinic A and B: university nephrology outpatient clinics; clinic C: non-university nephrology outpatient clinic. Ind 1: Patients with hypertension prescribed antihypertensives; Ind 2: Patients with albuminuria prescribed renin-angiotensin-aldosterone system (RAAS) inhibitors; Ind 4: Patients on multiple antihypertensives prescribed a combination of RAAS inhibitors and diuretics; Ind 6: Patients aged 50 to 65 years prescribed statins; Ind 7: Patients with high phosphate levels prescribed phosphate binders; Ind 10: Patients prescribed dual RAAS blockade; Ind 11: Patients with high calcium levels prescribed active vitamin D; Ind 12: Patients with normal haemoglobin levels prescribed erythropoietin stimulating agents; Ind 13: Patients with an estimated glomerular filtration rate (eGFR) lower than 30

For peer review only - http://bmjopen.bmj.com/site/about/guidelines.xhtml

ml/min/1.73m^2 prescribed high dose non-steroidal anti-inflammatory drugs (NSAIDs); Ind 14: Patients with diabetes and an eGFR lower than 30 ml/min/1.73m^2 prescribed metformin; Ind 15: Patients with an eGFR lower than 50 ml/min/1.73m^2 prescribed high dose digoxin; Ind 16: Patients prescribed a combination of NSAIDs, RAAS inhibitors and diuretics. 95% CIs were calculated based on included number of patients in the denominator of each indicator. * Significant difference between 2 or all outpatient clinics using Chi-square or Fisher's exact test with Bonferroni-correction for multiple testing.

SUPPORTING INFORMATION

Supplementary table 1. Baseline characteristics per nephrology outpatient clinic. eGFR: estimated glomerular filtration rate; MDRD: Modification of Diet in Renal Disease; SBP: systolic blood pressure; DBP: diastolic blood pressure. Clinic A and B: university nephrology outpatient clinics; clinic C: non-university nephrology outpatient clinic. [a] Median with interquartile range.

Supplementary table 2. Baseline table per chronic kidney disease stage per location. CKD: chronic kidney disease; SD: standard deviation; eGFR: estimated glomerular filtration rate; MDRD: Modification of Diet in Renal Disease; SBP: systolic blood pressure; DBP: diastolic blood pressure. Clinic A and B: university nephrology outpatient clinics; clinic C: non-university nephrology outpatient clinic. [a] Median with interquartile range.

Supplementary table 3. Differences between nephrology outpatient clinics per indicator stratified for chronic kidney disease stage. CKD: chronic kidney disease; RAAS: renin-angiotensin-aldosterone system; ESA: erythropoiesis-stimulating agent; NSAID: non-steroidal anti-inflammatory drug. Clinic A and B: university nephrology outpatient clinics; clinic C: non-university nephrology outpatient clinic. † Fisher's exact because of <5 expected number of patients in one or more cells.

For peer review only

For peer review only - http://bmjopen.bmj.com/site/about/guidelines.xhtml

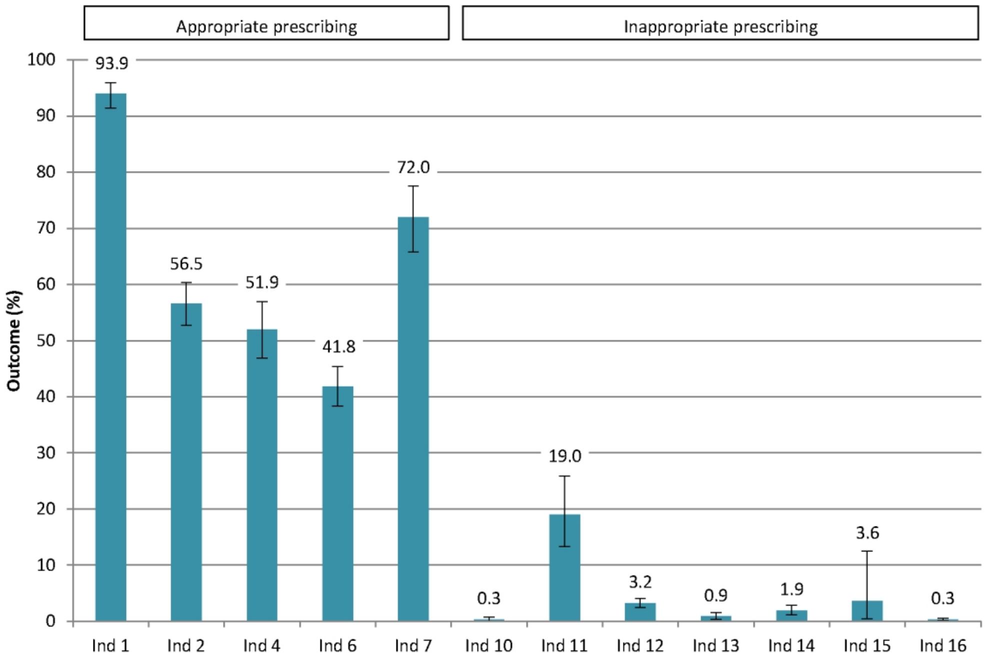

Fig 1. Overall prescribing quality assessed with 5 PQIs for appropriate prescribing (Ind 1-7) and 7 PQIs for potential inappropriate prescribing (Ind 10-16). Ind 1: Patients with hypertension prescribed antihypertensives; Ind 2: Patients with albuminuria prescribed renin-angiotensin-aldosterone system (RAAS) inhibitors; Ind 4: Patients on multiple antihypertensives prescribed a combination of RAAS inhibitors and diuretics; Ind 6: Patients aged 50 to 65 years prescribed statins; Ind 7: Patients with high phosphate levels prescribed phosphate binders; Ind 10: Patients prescribed dual RAAS blockade; Ind 11: Patients with high calcium levels prescribed active vitamin D; Ind 12: Patients with normal haemoglobin levels prescribed erythropoietin stimulating agents; Ind 13: Patients with an estimated glomerular filtration rate (eGFR) lower than 30 ml/min/1.73m2 prescribed high dose non-steroidal anti-inflammatory drugs (NSAIDs); Ind 14: Patients with diabetes and an eGFR lower than 30 ml/min/1.73m2 prescribed metformin; Ind 15: Patients with an eGFR lower than 50 ml/min/1.73m2 prescribed high dose digoxin; Ind 16: Patients prescribed a combination of NSAIDs, RAAS inhibitors and diuretics. 95% CIs were calculated based on included number of patients in the denominator of each indicator.

150x100mm (300 x 300 DPI)

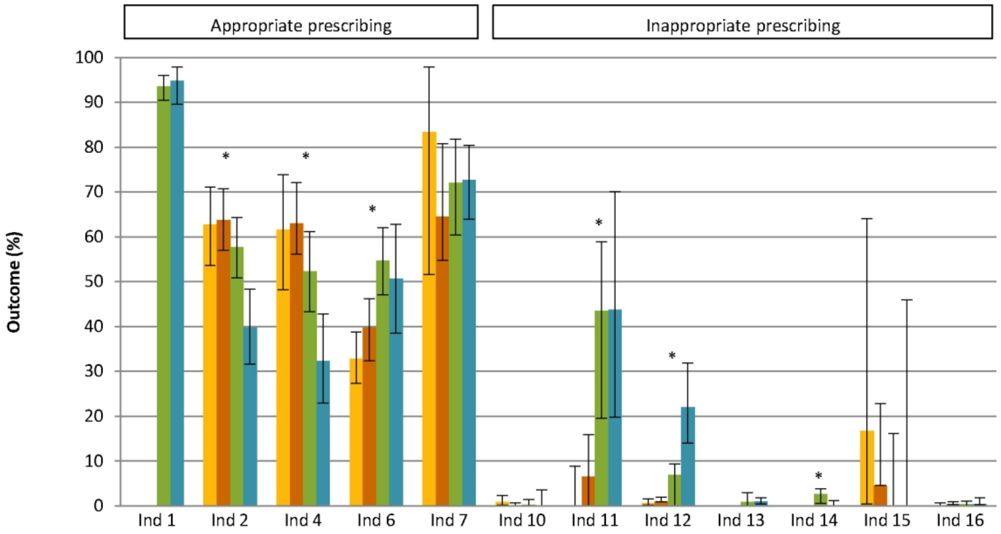

	Ind 1	Ind 2	Ind 4	Ind 6	Ind 7	Ind 10	Ind 11	Ind 12	Ind 13	Ind 14	Ind 15	Ind 16
Stage 3A		62.7	61.7	32.9	83.3	0.8	0.0	0.6			16.7	0.1
Stage 3B		63.8	63.0	39.7	64.5	0.0	6.6	1.0			4.5	0.4
Stage 4	93.6	57.7	52.3	54.6	72.0	0.2	43.5	6.8	0.8	2.6	0.0	0.3
Stage 5	94.8	39.7	32.3	50.7	72.7	0.0	43.8	22.0	1.0	0.0	0.0	0.3

Fig 2. Prescribing quality across different CKD stages (3A-5) assessed with 5 PQIs for appropriate prescribing (Ind 1-7) and 7 PQIs for potential inappropriate prescribing (Ind 10-16). Ind 1: Patients with hypertension prescribed antihypertensives; Ind 2: Patients with albuminuria prescribed renin-angiotensin-aldosterone system (RAAS) inhibitors; Ind 4: Patients on multiple antihypertensives prescribed a combination of RAAS inhibitors and diuretics; Ind 6: Patients aged 50 to 65 years prescribed statins; Ind 7: Patients with high phosphate levels prescribed phosphate binders; Ind 10: Patients prescribed dual RAAS blockade; Ind 11: Patients with high calcium levels prescribed active vitamin D; Ind 12: Patients with normal haemoglobin levels prescribed erythropoietin stimulating agents; Ind 13: Patients with an estimated glomerular filtration rate (eGFR) lower than 30 ml/min/1.73m2 prescribed high dose non-steroidal anti-inflammatory drugs (NSAIDs); Ind 14: Patients with diabetes and an eGFR lower than 30 ml/min/1.73m2 prescribed metformin; Ind 15: Patients with an eGFR lower than 50 ml/min/1.73m2 prescribed high dose digoxin; Ind 16: Patients prescribed a combination of NSAIDs, RAAS inhibitors and diuretics. 95% CIs were calculated based on included number of patients in the denominator of each indicator. * Significant difference between 2 or more CKD stages using Chi-square or Fisher's exact test with Bonferroni-correction for multiple testing.

150x100mm (300 x 300 DPI)

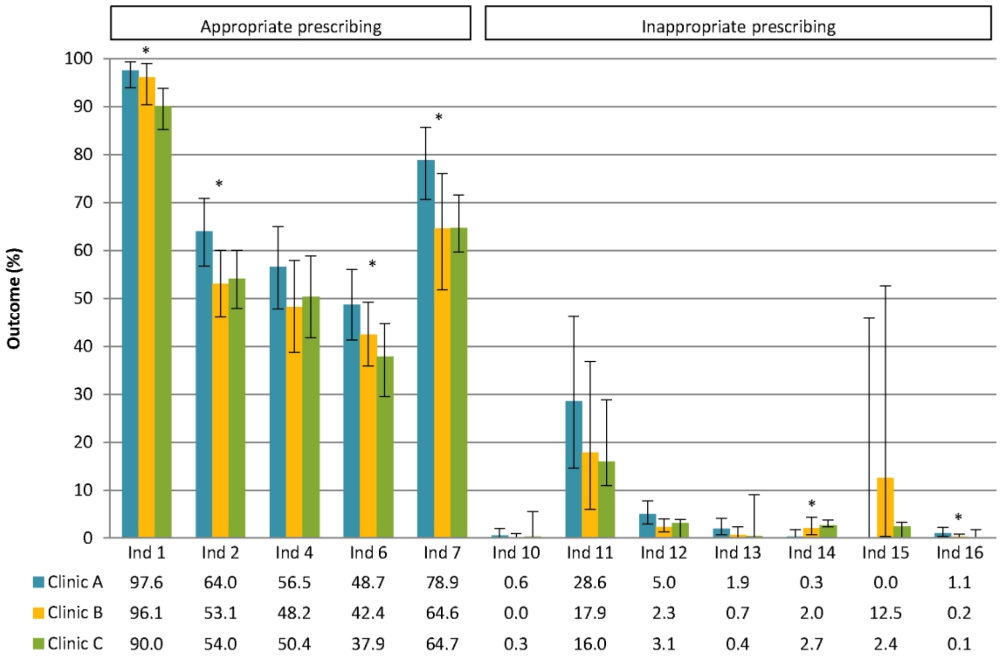

Fig 3: Prescribing quality across different outpatient clinics. Clinic A and B: university nephrology outpatient clinics; clinic C: non-university nephrology outpatient clinic. Ind 1: Patients with hypertension prescribed antihypertensives; Ind 2: Patients with albuminuria prescribed renin-angiotensin-aldosterone system (RAAS) inhibitors; Ind 4: Patients on multiple antihypertensives prescribed a combination of RAAS inhibitors and diuretics; Ind 6: Patients aged 50 to 65 years prescribed statins; Ind 7: Patients with high phosphate levels prescribed phosphate binders; Ind 10: Patients prescribed dual RAAS blockade; Ind 11: Patients with high calcium levels prescribed active vitamin D; Ind 12: Patients with normal haemoglobin levels prescribed erythropoietin stimulating agents; Ind 13: Patients with an estimated glomerular filtration rate (eGFR) lower than 30 ml/min/1.73m2 prescribed high dose non-steroidal anti-inflammatory drugs (NSAIDs); Ind 14: Patients with diabetes and an eGFR lower than 30 ml/min/1.73m2 prescribed metformin; Ind 15: Patients with an eGFR lower than 50 ml/min/1.73m2 prescribed high dose digoxin; Ind 16: Patients prescribed a combination of NSAIDs, RAAS inhibitors and diuretics. 95% CIs were calculated based on included number of patients in the denominator of each indicator. * Significant difference between 2 or all outpatient clinics using Chi-square or Fisher’s exact test with Bonferroni-correction for multiple testing.

150x100mm (300 x 300 DPI)

S1 Table. Baseline characteristics per nephrology outpatient clinic.

	Clinic A (n=569)		Clinic B (n=845)		Clinic C (n=1,718)	
	N (%)	Mean (±SD)	N (%)	Mean (±SD)	N (%)	Mean (±SD)
Age (years)	569 (100)	63.4 (±14.6)	845 (100)	65.0 (±15.3)	1,718 (100)	70.5 (±12.6)
< 50 years	98 (17.2)		134 (15.9)		131 (7.6)	
50-80 years	397 (69.8)		581 (68.8)		1,171 (68.2)	
>= 80 years	74 (13.0)		130 (15.4)		416 (24.2)	
Gender (males)	323 (56.8)		446 (52.8)		969 (56.4)	
Diabetes (yes)	145 (25.5)		164 (19.4)		176 (10.2)	
eGFR (MDRD) ml/min	569 (100)	28.4 (±14.2)	845 (100)	35.9 (±14.1)	1,718 (100)	36.5 (±13.1)
Stage 3a	92 (16.2)		255 (30.2)		496 (28.9)	
Stage 3b	166 (29.2)		295 (34.9)		664 (38.7)	
Stage 4	183 (32.2)		219 (25.9)		460 (26.8)	
Stage 5	128 (22.5)		76 (9.0)		98 (5.7)	
SBP (mmHg)	552 (97.0)	131.7 (±18.6)	470 (55.6)	133.4 (±20.7)	1,489 (86.7)	131.7 (±18.2)
Elevated SBP (>140 mmHg)	137 (24.1)	156.2 (±12.7)	143 (16.9)	158.0 (±13.3)	420 (24.4)	154.0 (±11.8)
DBP (mmHg)	552 (97.0)	72.0 (±12.4)	470 (55.6)	76.0 (±10.7)	1,489 (86.7)	76.0 (±10.7)
Low DBP (<70 mmHg)	202 (35.5)	59.4 (±6.9)	100 (11.8)	61.7 (±4.4)	402 (23.4)	62.6 (±5.2)
Total protein (g/24h urine)	513 (90.2)	0.4 [0.1-1.2][a]	244 (28.9)	0.9 [0.3-2.1][a]	557 (32.4)	0.3 [0.1-0.9][a]
Total protein (g/l urine)	515 (90.5)	0.2 [0.1-0.7][a]	574 (67.9)	0.3 [0.1-0.7][a]	1,239 (72.1)	0.2 [0.1-0.4][a]
Proteinuria (>0.5 g/24h or L urine)	215 (37.8)		238 (28.2)		357 (20.8)	
Phosphate (mmol/l)	544 (95.6)	1.20 (±0.33)	623 (73.7)	1.10 (±0.32)	1,439 (83.8)	1.02 (±0.24)
Elevated phosphate (>1.49 mmol/l)	78 (13.7)	1.79 (±0.33)	49 (5.8)	1.87 (±0.40)	45 (2.6)	1.73 (±0.29)
Calcium (mmol/l)	546 (96.0)	2.35 (±0.17)	663 (78.5)	2.34 (±0.14)	1,525 (88.8)	2.37 (±0.12)
Elevated Calcium (>2.54 mmol/l)	35 (6.2)	2.62 (±0.06)	28 (3.3)	2.62 (±0.06)	100 (5.8)	2.62 (±0.09)
Haemoglobin level (mmol/l)	565 (99.3)	7.75 (±1.09)	808 (95.6)	8.0 (±1.2)	1,651 (96.1)	8.1 (±1.1)
Low haemoglobin level (<7.5 mmol/l)	222 (39.0)	6.7 (±0.6)	253 (29.9)	6.7 (±0.6)	458 (26.7)	6.8 (±0.6)

eGFR: estimated glomerular filtration rate; MDRD: Modification of Diet in Renal Disease; SBP: systolic blood pressure; DBP: diastolic blood pressure.
Clinic A and B: university nephrology outpatient clinics; clinic C: non-university nephrology outpatient clinic.
[a] Median with interquartile range.

For peer review only

S2 Table. Baseline table per chronic kidney disease stage per location.

	Clinic A (N=92)		Clinic B (N=255)		Clinic C (N=496)	
**CKD stage 3a**	**N (%)**	**Mean (±SD)**	**N (%)**	**Mean (±SD)**	**N (%)**	**Mean (±SD)**
Age (years)	92 (100)	56.8 (±13.5)	255 (100)	60.5 (±14.9)	496 (100)	65.6 (±13.4)
< 50 years	26 (28.3)		56 (22.0)		69 (13.9)	
50-80 years	62 (67.4)		182 (71.4)		360 (72.6)	
>= 80 years	4 (4.4)		17 (6.7)		67 (13.5)	
Gender (males)	46 (50.0)		135 (52.9)		275 (55.4)	
Diabetes (yes)	14 (15.2)		39 (15.3)		43 (8.7)	
eGFR (MDRD) ml/min	92 (100)	51.1 (±4.2)	255 (100)	52.4 (±4.2)	496 (100)	52.3 (±4.4)
SBP (mmHg)	91 (98.9)	126.3 (±17.4)	122 (47.8)	128.5 (±18.8)	388 (78.2)	130.4 (±16.7)
Elevated SBP (>140 mmHg)	12 (13.0)	157.1 (±15.7)	23 (9.0)	157.4 (±12.9)	84 (16.9)	154.5 (±11.7)
DBP (mmHg)	91 (98.9)	73.4 (±12.5)	122 (47.8)	76.0 (±10.5)	388 (78.2)	77.6 (±10.0)
Low DBP (<70 mmHg)	31 (33.7)	59.8 (±6.1)	22 (8.6)	61.3 (±4.4)	77 (15.5)	63.2 (±5.2)
Total protein (g/24h urine)	84 (91.3)	0.2 [0.1-1.0][a]	62 (24.3)	0.5 [0.3-1.3][a]	132 (26.6)	0.2 [0.1-0.5][a]
Total protein (g/l urine)	84 (91.3)	0.1 [0.1-0.4][a]	165 (65.7)	0.1 [0.0-0.4][a]	303 (61.1)	0.1 [0.1-0.3][a]
Proteinuria (>0.5 g/24h or L urine)	29 (31.5)		52 (20.4)		59 (11.9)	
Phosphate (mmol/l)	83 (90.2)	1.02 (±0.20)	139 (54.5)	0.98 (±0.22)	317 (63.9)	0.94 (±0.19)
Elevated phosphate (>1.49 mmol/l)	0 (0.0)	-	2 (0.8)	1.72 (±0.30)	1 (0.2)	1.63 (-)
Calcium (mmol/l)	84 (91.3)	2.38 (±0.14)	159 (62.4)	2.37 (±0.11)	373 (75.2)	2.38 (±0.11)
Elevated Calcium (>2.54 mmol/l)	6 (6.5)	2.60 (±0.05)	7 (2.7)	2.62 (±0.06)	27 (5.4)	2.62 (±0.08)
Haemoglobin level (mmol/l)	90 (97.8)	8.4 (±1.2)	231 (90.6)	8.6 (±1.0)	450 (90.7)	8.4 (±1.0)
Low haemoglobin level (<7.5 mmol/l)	15 (16.3)	6.6 (±0.5)	29 (11.4)	6.8 (±0.6)	69 (13.9)	6.8 (±0.6)
**CKD stage 3b**	**Clinic A (N=166)**		**Clinic B (N=295)**		**Clinic C (N=664)**	
	**N (%)**	**Mean (±SD)**	**N (%)**	**Mean (±SD)**	**N (%)**	**Mean (±SD)**
Age	166 (100)	63.4 (±14.3)	295 (100)	66.9 (±14.8)	664 (100)	71.2 (±11.5)
< 50 years	33 (19.9)		40 (13.6)		40 (6.0)	
50-80 years	115 (69.3)		200 (67.8)		478 (72.0)	
>= 80 years	18 (10.8)		55 (18.6)		146 (22.0)	
Gender (males)	98 (59.0)		147 (49.8)		369 (55.6)	
Diabetes (yes)	44 (26.5)		54 (18.3)		68 (10.2)	
eGFR (MDRD) ml/min	166 (100)	36.4 (±4.4)	295 (100)	37.6 (±4.1)	664 (100)	37.4 (±4.2)

For peer review only

SBP (mmHg)	163 (98.2)	129.6 (±17.0)	160 (54.2)	131.6 (±21.0)	587 (88.4)	130.7 (±18.3)
Elevated SBP (>140 mmHg)	33 (19.9)	153.8 (±14.2)	49 (16.6)	156.6 (±12.9)	157 (23.4)	153.7 (±11.9)
DBP (mmHg)	163 (98.2)	72.4 (±12.4)	160 (54.2)	75.5 (±10.3)	587 (88.4)	75.4 (±11.1)
Low DBP (<70 mmHg)	61 (36.7)	60.2 (±6.6)	35 (11.9)	61.8 (±4.2)	186 (28.0)	62.8 (±5.0)
Total protein (g/24h urine)	148 (89.2)	0.2 [0.1-0.6][a]	67 (22.7)	0.7 [0.2-2.0][a]	211 (31.8)	0.2 [0.1-0.7][a]
Total protein (g/l urine)	150 (90.4)	0.2 [0.1-0.4][a]	190 (64.4)	0.2 [0.1-0.5][a]	501 (75.5)	0.2 [0.1-0.4][a]
Proteinuria (>0.5 g/24h or L urine)	39 (23.5)		63 (21.4)		122 (18.4)	
Phosphate (mmol/l)	160 (96.4)	1.04 (±0.21)	213 (72.2)	1.02 (±0.22)	586 (88.3)	0.98 (±0.19)
Elevated phosphate (>1.49 mmol/l)	5 (3.0)	1.58 (±0.04)	6 (2.0)	1.80 (±0.28)	6 (0.9)	1.57 (±0.08)
Calcium (mmol/l)	160 (96.4)	2.37 (±0.15)	231 (78.3)	2.34 (±0.12)	607 (91.4)	2.37 (±0.13)
Elevated Calcium (>2.54 mmol/l)	10 (6.0)	2.62 (±0.06)	7 (2.4)	2.61 (±0.06)	44 (6.6)	2.63 (±0.10)
Haemoglobin level (mmol/l)	166 (100)	8.1 (±1.0)	284 (96.3)	8.1 (±1.1)	645 (97.1)	8.2 (±1.0)
Low haemoglobin level (<7.5 mmol/l)	37 (22.2)	6.9 (±0.5)	81 (27.5)	6.8 (±0.5)	146 (22.0)	6.8 (±0.5)
**CKD stage 4**	**Clinic A (N=183)**		**Clinic B (N=219)**		**Clinic C (N=460)**	
	**N (%)**	**Mean (±SD)**	**N (%)**	**Mean (±SD)**	**N (%)**	**Mean (±SD)**
Age	183 (100)	65.3 (±14.6)	219 (100)	67.4 (±15.2)	460 (100)	73.4 (±11.8)
< 50 years	24 (13.1)		26 (11.9)		20 (4.4)	
50-80 years	126 (68.9)		149 (68.0)		281 (61.1)	
>= 80 years	33 (18.0)		44 (20.1)		159 (34.6)	
Gender (males)	98 (53.6)		119 (54.3)		271 (58.9)	
Diabetes (yes)	55 (30.1)		58 (26.5)		52 (11.3)	
eGFR (MDRD) ml/min	183 (100)	22.3 (±4.2)	219 (100)	23.0 (±4.3)	460 (100)	23.4 (±4.2)
SBP (mmHg)	176 (96.2)	132.0 (±19.4)	140 (63.9)	136.8 (±21.0)	419 (91.1)	132.6 (±18.7)
Elevated SBP (>140 mmHg)	48 (26.2)	156.8 (±11.4)	50 (22.8)	159.1 (±13.8)	135 (29.3)	153.5 (±11.5)
DBP (mmHg)	176 (96.2)	71.6 (±12.8)	140 (63.9)	75.1 (±10.9)	419 (91.1)	75.7 (±10.3)
Low DBP (<70 mmHg)	66 (36.1)	59.0 (±7.6)	38 (17.4)	61.7 (±4.7)	110 (23.9)	62.6 (±5.1)
Total protein (g/24h urine)	163 (89.1)	0.4 [0.1-1.0][a]	75 (34.2)	0.9 [0.3-2.3][a]	165 (35.9)	0.4 [0.2-1.3][a]
Total protein (g/l urine)	163 (89.1)	0.2 [0.1-0.6][a]	162 (74.0)	0.3 [0.2-0.8][a]	362 (78.7)	0.2 [0.1-0.6][a]
Proteinuria (>0.5 g/24h or L urine)	63 (34.4)		77 (35.2)		128 (27.8)	
Phosphate (mmol/l)	176 (96.2)	1.18 (±0.23)	200 (91.3)	1.14 (±0.32)	439 (95.4)	1.05 (±0.2)
Elevated phosphate (>1.49 mmol/l)	16 (8.7)	1.62 (±0.11)	17 (7.8)	1.84 (±0.47)	8 (1.7)	1.66 (±0.11)
Calcium (mmol/l)	176 (96.2)	2.35 (±0.20)	199 (90.9)	2.34 (±0.16)	447 (97.2)	2.36 (±0.12)
Elevated Calcium (>2.54 mmol/l)	13 (7.1)	2.61 (±0.07)	9 (4.1)	2.65 (±0.07)	24 (5.2)	2.62 (±0.06)

For peer review only

Haemoglobin level (mmol/l)	182 (99.5)	7.6 (±1.0)	217 (99.1)	7.7 (±1.2)	458 (99.6)	7.8 (±1.0)
Low haemoglobin level (<7.5 mmol/l)	82 (44.8)	6.7 (±0.7)	89 (40.6)	6.6 (±0.6)	175 (38.0)	6.7 (±0.6)
**CKD stage 5**	**Clinic A (N=128)**		**Clinic B (N=76)**		**Clinic (N=98)**	
	**N (%)**	**Mean (±SD)**	**N (%)**	**Mean (±SD)**	**N (%)**	**Mean (±SD)**
Age	128 (100)	65.5 (±14.7)	100 (76)	65.5 (±15.9)	100 (98)	76.9 (±10.6)
< 50 years	15 (11.7)		12 (15.8)		2.0 (2.0)	
50-80 years	94 (73.4)		40 (65.8)		52 (53.1)	
>= 80 years	19 (14.8)		14 (18.4)		44 (44.9)	
Gender (males)	81 (63.3)		45 (59.2)		54 (55.1)	
Diabetes (yes)	32 (25.0)		13 (17.1)		13 (13.3)	
eGFR (MDRD) ml/min	128 (100)	10.5 (±2.6)	76 (100)	11.2 (±2.4)	98 (100)	11.9 (±2.6)
SBP (mmHg)	122 (95.3)	137.9 (±18.6)	48 (63.2)	142.1 (±19.8)	95 (96.9)	139.2 (±19.7)
Elevated SBP (>140 mmHg)	44 (34.4)	157.1 (±12.2)	21 (27.6)	159.6 (±14.2)	44 (44.9)	155.9 (±12.2)
DBP (mmHg)	122 (95.3)	71.1 (±11.6)	48 (63.2)	80.6 (±11.4)	95 (96.9)	74.4 (±11.9)
Low DBP (<70 mmHg)	44 (34.4)	58.5 (±6.7)	5 (6.6)	62.2 (±4.1)	29 (29.6)	60.2 (±6.4)
Total protein (g/24h urine)	118 (92.2)	1.3 [0.5-2.7][a]	40 (52.6)	1.9 [0.9-4.1][a]	49 (50.0)	1.0 [0.4-2.6][a]
Total protein (g/l urine)	118 (92.2)	0.7 [0.3-1.4][a]	57 (75.0)	1.4 [0.5-2.4][a]	73 (74.5)	0.6 [0.3-1.1][a]
Proteinuria (>0.5 g/24h or L urine)	84 (65.6)		57 (60.5)		48 (49.0)	
Phosphate (mmol/l)	125 (97.7)	1.55 (±0.39)	71 (93.4)	1.47 (±0.42)	97 (99.0)	1.39 (±0.36)
Elevated phosphate (>1.49 mmol/l)	57 (44.5)	1.86 (±0.36)	24 (31.6)	1.92 (±0.39)	30 (30.6)	1.79 (±0.34)
Calcium (mmol/l)	126 (98.4)	2.31 (±0.16)	74 (97.4)	2.27 (±0.19)	98 (100)	2.32 (±0.14)
Elevated Calcium (>2.54 mmol/l)	13 (10.2)	2.65 (±0.07)	5 (6.6)	2.63 (±0.08)	5 (5.1)	2.66 (±0.11)
Haemoglobin level (mmol/l)	127 (99.2)	7.0 (±0.8)	76 (100)	7.0 (±1.2)	98 (100)	7.0 (±0.9)
Low haemoglobin level (<7.5 mmol/l)	88 (68.8)	6.6 (±0.5)	54 (71.1)	6.4 (±0.8)	68 (69.4)	6.6 (±0.7)

CKD: chronic kidney disease; SD: standard deviation; eGFR: estimated glomerular filtration rate; MDRD: Modification of Diet in Renal Disease; SBP: systolic blood pressure; DBP: diastolic blood pressure.

Clinic A and B: university nephrology outpatient clinics; clinic C: non-university nephrology outpatient clinic.

[a] Median with interquartile range.

For peer review only

S3 Table. Differences between nephrology outpatient clinics per indicator stratified for chronic kidney disease stage.

Indicator 1 - Antihypertensive use

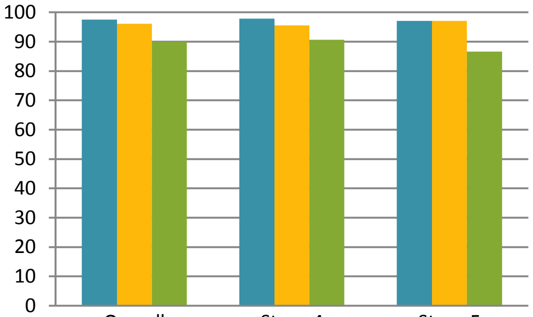

	Overall	Stage 4	Stage 5
Clinic A	97.6	97.9	97.1
Clinic B	96.1	95.6	97.1
Clinic C	90.0	90.6	86.7

Overall	*p-value χ^2*
Clinic A vs. Clinic B	0.316
Clinic A vs. Clinic C	**0.003**
Clinic B vs. Clinic C	0.095
CKD stage 4	
Clinic A vs. Clinic B	0.406[†]
Clinic A vs. Clinic C	**0.011**
Clinic B vs. Clinic C	0.196
CKD stage 5	
Clinic A vs. Clinic B	1.000[†]
Clinic A vs. Clinic C	0.103[†]
Clinic B vs. Clinic C	0.306[†]

Indicator 2 - RAASi with proteinuria

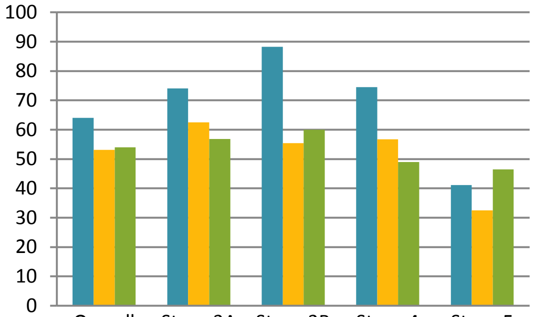

	Overall	Stage 3A	Stage 3B	Stage 4	Stage 5
Clinic A	64.0	74.1	88.2	74.5	41.1
Clinic B	53.1	62.5	55.4	56.7	32.5
Clinic C	54.0	56.9	60.0	49.0	46.4

Overall	*p-value χ^2*
Clinic A vs. Clinic B	0.030
Clinic A vs. Clinic C	**0.003**
Clinic B vs. Clinic C	0.503
CKD stage 3a	
Clinic A vs. Clinic B	0.190
Clinic A vs. Clinic C	0.095
Clinic B vs. Clinic C	0.675
CKD stage 3b	
Clinic A vs. Clinic B	**0.002**
Clinic A vs. Clinic C	**0.001**
Clinic B vs. Clinic C	0.819
CKD stage 4	
Clinic A vs. Clinic B	0.089
Clinic A vs. Clinic C	**0.001**
Clinic B vs. Clinic C	0.117
CKD stage 5	
Clinic A vs. Clinic B	0.209
Clinic A vs. Clinic C	0.243
Clinic B vs. Clinic C	0.923

For peer review only

Indicator 4 - RAASi and diuretics

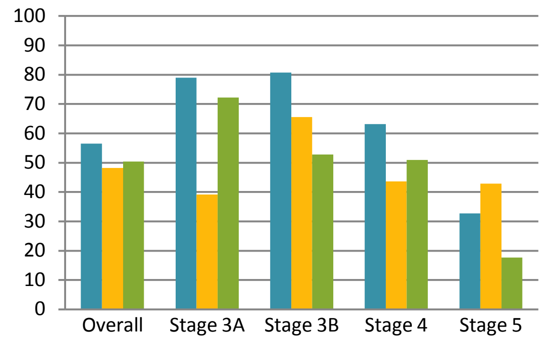

	Overall	Stage 3A	Stage 3B	Stage 4	Stage 5
Clinic A	56.5	78.9	80.8	63.2	32.7
Clinic B	48.2	39.1	65.5	43.6	42.9
Clinic C	50.4	72.2	52.8	50.9	17.6

Overall	*p-value χ^2*
Clinic A vs. Clinic B	0.184
Clinic A vs. Clinic C	0.081
Clinic B vs. Clinic C	0.786
CKD stage 3a	
Clinic A vs. Clinic B	**0.014**
Clinic A vs. Clinic C	1.000
Clinic B vs. Clinic C	**0.014**
CKD stage 3b	
Clinic A vs. Clinic B	0.068
Clinic A vs. Clinic C	**0.002**
Clinic B vs. Clinic C	0.235
CKD stage 4	
Clinic A vs. Clinic B	0.245
Clinic A vs. Clinic C	0.337
Clinic B vs. Clinic C	0.766
CKD stage 5	
Clinic A vs. Clinic B	0.586
Clinic A vs. Clinic C	0.033
Clinic B vs. Clinic C	0.021

Indicator 6 - Statin use

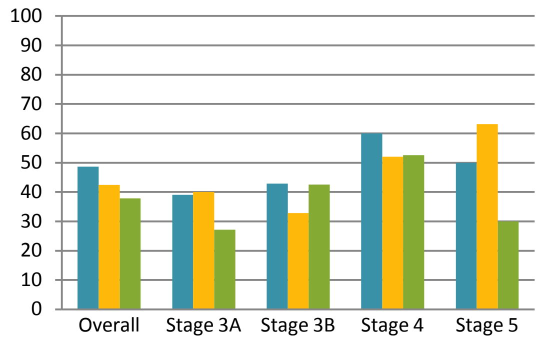

	Overall	Stage 3A	Stage 3B	Stage 4	Stage 5
Clinic A	48.7	39.0	42.9	60.0	50.0
Clinic B	42.4	40.0	32.9	52.0	63.2
Clinic C	37.9	27.2	42.5	52.6	30.0

Overall	*p-value χ^2*
Clinic A vs. Clinic B	0.205
Clinic A vs. Clinic C	**0.015**
Clinic B vs. Clinic C	0.277
CKD stage 3a	
Clinic A vs. Clinic B	0.916
Clinic A vs. Clinic C	0.140
Clinic B vs. Clinic C	0.042
CKD stage 3b	
Clinic A vs. Clinic B	0.266
Clinic A vs. Clinic C	0.966
Clinic B vs. Clinic C	0.189
CKD stage 4	
Clinic A vs. Clinic B	0.409
Clinic A vs. Clinic C	0.395
Clinic B vs. Clinic C	0.950
CKD stage 5	
Clinic A vs. Clinic B	0.340
Clinic A vs. Clinic C	0.309†
Clinic B vs. Clinic C	0.128†

Indicator 7 - Phosphate binder use

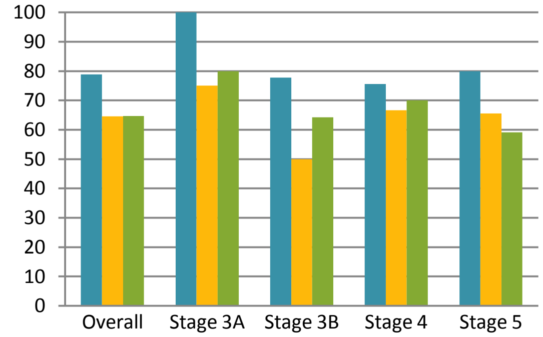

	Overall	Stage 3A	Stage 3B	Stage 4	Stage 5
Clinic A	78.9	100.0	77.8	75.6	80.0
Clinic B	64.6	75.0	50.0	66.7	65.5
Clinic C	64.7	80.0	64.3	70.0	59.1

Overall	*p-value χ^2*
Clinic A vs. Clinic B	**0.016**

Indicator 10 - Dual RAAS blockade

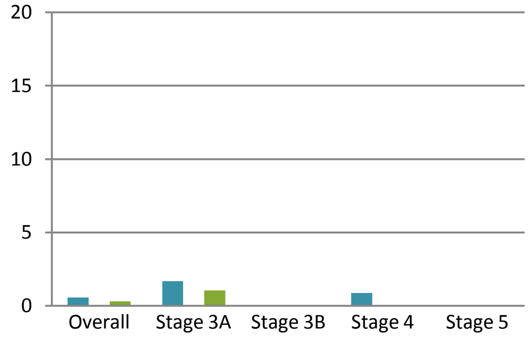

	Overall	Stage 3A	Stage 3B	Stage 4	Stage 5
Clinic A	0.6	1.7	0.0	0.9	0.0
Clinic B	0.0	0.0	0.0	0.0	0.0
Clinic C	0.3	1.0	0.0	0.0	0.0

Overall	*p-value χ^2*
Clinic A vs. Clinic B	0.218†

Clinic A vs. Clinic C	0.030
Clinic B vs. Clinic C	0.849
CKD stage 3a	
Clinic A vs. Clinic B	1.000†
Clinic A vs. Clinic C	1.000†
Clinic B vs. Clinic C	1.000†
CKD stage 3b	
Clinic A vs. Clinic B	0.650†
Clinic A vs. Clinic C	1.000†
Clinic B vs. Clinic C	0.692†
CKD stage 4	
Clinic A vs. Clinic B	0.191
Clinic A vs. Clinic C	0.548
Clinic B vs. Clinic C	0.620
CKD stage 5	
Clinic A vs. Clinic B	0.067
Clinic A vs. Clinic C	**0.013**
Clinic B vs. Clinic C	0.575

Clinic A vs. Clinic C	0.612†
Clinic B vs. Clinic C	0.530†
CKD stage 3a	
Clinic A vs. Clinic B	0.323†
Clinic A vs. Clinic C	0.558†
Clinic B vs. Clinic C	0.521†
CKD stage 3b	
Clinic A vs. Clinic B	No prescriptions
Clinic A vs. Clinic C	No prescriptions
Clinic B vs. Clinic C	No prescriptions
CKD stage 4	
Clinic A vs. Clinic B	1.000†
Clinic A vs. Clinic C	0.391†
Clinic B vs. Clinic C	No prescriptions
CKD stage 5	
Clinic A vs. Clinic B	No prescriptions
Clinic A vs. Clinic C	No prescriptions
Clinic B vs. Clinic C	No prescriptions

Indicator 11 - Vitamin D use

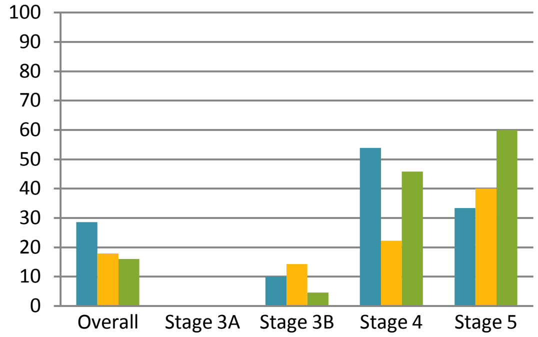

	Overall	Stage 3A	Stage 3B	Stage 4	Stage 5
Clinic A	28.6	0.0	10.0	53.8	33.3
Clinic B	17.9	0.0	14.3	22.2	40.0
Clinic C	16.0	0.0	4.5	45.8	60.0

Indicator 12 - ESA use

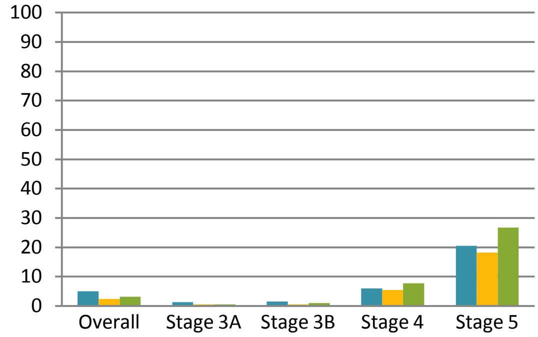

	Overall	Stage 3A	Stage 3B	Stage 4	Stage 5
Clinic A	5.0	1.3	1.6	6.0	20.5
Clinic B	2.3	0.5	0.5	5.5	18.2
Clinic C	3.1	0.5	1.0	7.8	26.7

Overall	*p-value χ^2*
Clinic A vs. Clinic B	0.321
Clinic A vs. Clinic C	0.105
Clinic B vs. Clinic C	0.779†
CKD stage 3a	
Clinic A vs. Clinic B	No prescriptions
Clinic A vs. Clinic C	No prescriptions
Clinic B vs. Clinic C	No prescriptions
CKD stage 3b	
Clinic A vs. Clinic B	1.000†
Clinic A vs. Clinic C	0.466†
Clinic B vs. Clinic C	0.364†
CKD stage 4	
Clinic A vs. Clinic B	0.203†
Clinic A vs. Clinic C	0.642
Clinic B vs. Clinic C	0.263†
CKD stage 5	
Clinic A vs. Clinic B	1.000†
Clinic A vs. Clinic C	0.567†

Overall	*p-value χ^2*
Clinic A vs. Clinic B	0.034
Clinic A vs. Clinic C	0.100
Clinic B vs. Clinic C	0.375
CKD stage 3a	
Clinic A vs. Clinic B	0.469†
Clinic A vs. Clinic C	0.417†
Clinic B vs. Clinic C	1.000†
CKD stage 3b	
Clinic A vs. Clinic B	0.562†
Clinic A vs. Clinic C	0.637†
Clinic B vs. Clinic C	0.678†
CKD stage 4	
Clinic A vs. Clinic B	0.864
Clinic A vs. Clinic C	0.558
Clinic B vs. Clinic C	0.398
CKD stage 5	
Clinic A vs. Clinic B	1.000†
Clinic A vs. Clinic C	0.548

Clinic B vs. Clinic C	1.000†

Clinic B vs. Clinic C	0.473

Indicator 13 - NSAIDs use

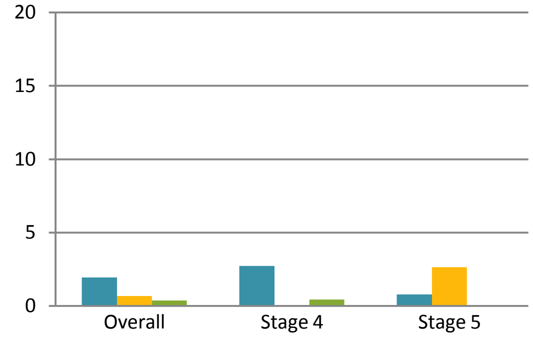

	Overall	Stage 4	Stage 5
Clinic A	1.9	2.7	0.8
Clinic B	0.7	0.0	2.6
Clinic C	0.4	0.4	0.0

Overall	*p-value* χ^2
Clinic A vs. Clinic B	0.287†
Clinic A vs. Clinic C	0.028†
Clinic B vs. Clinic C	0.612†
CKD stage 4	
Clinic A vs. Clinic B	0.019†
Clinic A vs. Clinic C	0.022†
Clinic B vs. Clinic C	1.000†
CKD stage 5	
Clinic A vs. Clinic B	0.557†
Clinic A vs. Clinic C	1.000†
Clinic B vs. Clinic C	0.189†

Indicator 14 - Metformin use

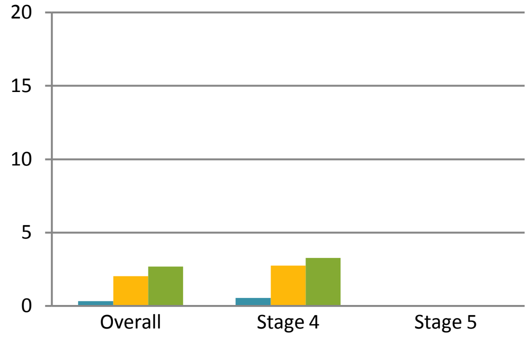

	Overall	Stage 4	Stage 5
Clinic A	0.3	0.5	0.0
Clinic B	2.0	2.7	0.0
Clinic C	2.7	3.3	0.0

Overall	*p-value* χ^2
Clinic A vs. Clinic B	0.062
Clinic A vs. Clinic C	**0.013**
Clinic B vs. Clinic C	0.558
CKD stage 4	
Clinic A vs. Clinic B	0.132†
Clinic A vs. Clinic C	0.050†
Clinic B vs. Clinic C	0.714
CKD stage 5	
Clinic A vs. Clinic B	No prescriptions
Clinic A vs. Clinic C	No prescriptions
Clinic B vs. Clinic C	No prescriptions

Indicator 15 - HD digoxin use

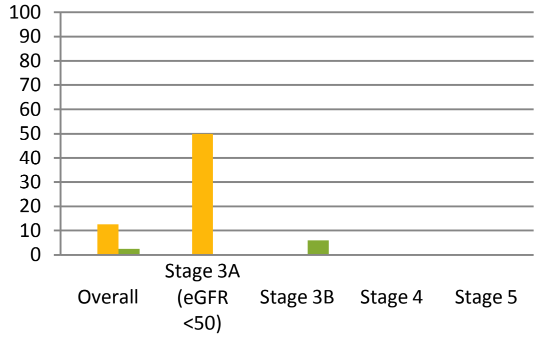

	Overall	Stage 3A (eGFR <50)	Stage 3B	Stage 4	Stage 5
Clinic A	0.0	n.a.	0.0	0.0	0.0
Clinic B	12.5	50.0	0.0	0.0	n.a.
Clinic C	2.4	0.0	5.9	0.0	0.0

Overall	*p-value* χ^2
Clinic A vs. Clinic B	1.000†
Clinic A vs. Clinic C	1.000†
Clinic B vs. Clinic C	0.202†
CKD stage 3a	
Clinic A vs. Clinic B	No prescriptions
Clinic A vs. Clinic C	No prescriptions
Clinic B vs. Clinic C	0.333†
CKD stage 3b	

Indicator 16 - NSAIDs, RAAS and diuretics

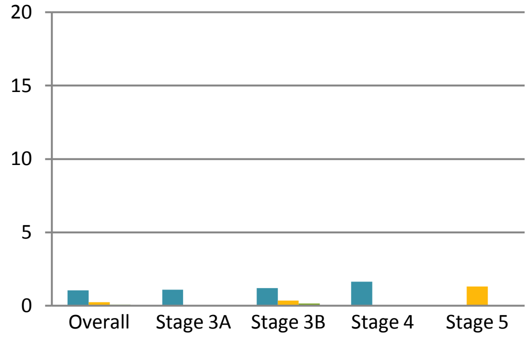

	Overall	Stage 3A	Stage 3B	Stage 4	Stage 5
Clinic A	1.1	1.1	1.2	1.6	0.0
Clinic B	0.2	0.0	0.3	0.0	1.3
Clinic C	0.1	0.0	0.2	0.0	0.0

Overall	*p-value* χ^2
Clinic A vs. Clinic B	0.067†
Clinic A vs. Clinic C	**0.001†**
Clinic B vs. Clinic C	0.254†
CKD stage 3a	
Clinic A vs. Clinic B	0.265†
Clinic A vs. Clinic C	0.156†
Clinic B vs. Clinic C	No prescriptions
CKD stage 3b	

For peer review only - http://bmjopen.bmj.com/site/about/guidelines.xhtml

Clinic A vs. Clinic B	No prescriptions	Clinic A vs. Clinic B	0.295†
Clinic A vs. Clinic C	1.000†	Clinic A vs. Clinic C	0.104†
Clinic B vs. Clinic C	1.000†	Clinic B vs. Clinic C	0.521†
CKD stage 4		*CKD stage 4*	
Clinic A vs. Clinic B	No prescriptions	Clinic A vs. Clinic B	0.093†
Clinic A vs. Clinic C	No prescriptions	Clinic A vs. Clinic C	0.023†
Clinic B vs. Clinic C	No prescriptions	Clinic B vs. Clinic C	1.000†
CKD stage 5		*CKD stage 5*	
Clinic A vs. Clinic B	No prescriptions	Clinic A vs. Clinic B	0.373†
Clinic A vs. Clinic C	No prescriptions	Clinic A vs. Clinic C	No prescriptions
Clinic B vs. Clinic C	No prescriptions	Clinic B vs. Clinic C	0.437†

CKD: chronic kidney disease; RAAS: renin-angiotensin-aldosterone system; ESA: erythropoiesis-stimulating agent; NSAID: non-steroidal anti-inflammatory drug.

Clinic A and B: university nephrology outpatient clinics; clinic C: non-university nephrology outpatient clinic.

Indicators 3, 5, 8 and 9 were excluded because of insufficient validity.

† Fisher's exact because of <5 expected number of patients in one or more cells.

For peer review only

STROBE 2007 (v4) Statement—Checklist of items that should be included in reports of *cohort studies*

**Section/Topic**	**Item #**	**Recommendation**	**Reported on page #**
**Title and abstract**	1	(*a*) Indicate the study's design with a commonly used term in the title or the abstract	1
		(*b*) Provide in the abstract an informative and balanced summary of what was done and what was found	2-3
**Introduction**			
Background/rationale	2	Explain the scientific background and rationale for the investigation being reported	3-4
Objectives	3	State specific objectives, including any prespecified hypotheses	4
**Methods**			
Study design	4	Present key elements of study design early in the paper	4
Setting	5	Describe the setting, locations, and relevant dates, including periods of recruitment, exposure, follow-up, and data collection	5
Participants	6	(*a*) Give the eligibility criteria, and the sources and methods of selection of participants. Describe methods of follow-up	4-5
		(*b*) For matched studies, give matching criteria and number of exposed and unexposed	NA
Variables	7	Clearly define all outcomes, exposures, predictors, potential confounders, and effect modifiers. Give diagnostic criteria, if applicable	5-7
Data sources/ measurement	8*	For each variable of interest, give sources of data and details of methods of assessment (measurement). Describe comparability of assessment methods if there is more than one group	7
Bias	9	Describe any efforts to address potential sources of bias	7
Study size	10	Explain how the study size was arrived at	4-5
Quantitative variables	11	Explain how quantitative variables were handled in the analyses. If applicable, describe which groupings were chosen and why	7
Statistical methods	12	(*a*) Describe all statistical methods, including those used to control for confounding	7
		(*b*) Describe any methods used to examine subgroups and interactions	7
		(*c*) Explain how missing data were addressed	7
		(*d*) If applicable, explain how loss to follow-up was addressed	NA
		(*e*) Describe any sensitivity analyses	NA
**Results**			

For peer review only

Participants	13*	(a) Report numbers of individuals at each stage of study—eg numbers potentially eligible, examined for eligibility, confirmed eligible, included in the study, completing follow-up, and analysed	8
		(b) Give reasons for non-participation at each stage	NA
		(c) Consider use of a flow diagram	NA
Descriptive data	14*	(a) Give characteristics of study participants (eg demographic, clinical, social) and information on exposures and potential confounders	8-9
		(b) Indicate number of participants with missing data for each variable of interest	9
		(c) Summarise follow-up time (eg, average and total amount)	NA
Outcome data	15*	Report numbers of outcome events or summary measures over time	10
Main results	16	(*a*) Give unadjusted estimates and, if applicable, confounder-adjusted estimates and their precision (eg, 95% confidence interval). Make clear which confounders were adjusted for and why they were included	10-11
		(*b*) Report category boundaries when continuous variables were categorized	NA
		(*c*) If relevant, consider translating estimates of relative risk into absolute risk for a meaningful time period	NA
Other analyses	17	Report other analyses done—eg analyses of subgroups and interactions, and sensitivity analyses	10-11
**Discussion**			
Key results	18	Summarise key results with reference to study objectives	11-12
**Limitations**			
Interpretation	20	Give a cautious overall interpretation of results considering objectives, limitations, multiplicity of analyses, results from similar studies, and other relevant evidence	13-14
Generalisability	21	Discuss the generalisability (external validity) of the study results	14
**Other information**			
Funding	22	Give the source of funding and the role of the funders for the present study and, if applicable, for the original study on which the present article is based	15

*Give information separately for cases and controls in case-control studies and, if applicable, for exposed and unexposed groups in cohort and cross-sectional studies.

Note: An Explanation and Elaboration article discusses each checklist item and gives methodological background and published examples of transparent reporting. The STROBE checklist is best used in conjunction with this article (freely available on the Web sites of PLoS Medicine at http://www.plosmedicine.org/, Annals of Internal Medicine at http://www.annals.org/, and Epidemiology at http://www.epidem.com/). Information on the STROBE Initiative is available at www.strobe-statement.org.

For peer review only

BMJ Open

Prescribing quality in secondary care patients with different stages of chronic kidney disease: a retrospective study in the Netherlands

Journal:	*BMJ Open*
Manuscript ID	bmjopen-2018-025784.R1
Article Type:	Research
Date Submitted by the Author:	23-Apr-2019

Complete List of Authors:	Smits, Kirsten; University Medical Center Groningen, Department of Clinical Pharmacy and Pharmacology<br>Sidorenkov, Grigory; University Medical Center Groningen, Department of Clinical Pharmacy and Pharmacology<br>van Ittersum, Frans; VU medisch centrum School of Medical Sciences, Department of Nephrology<br>Waanders, Femke; Isala, Department of Nephrology<br>Bilo, Henk; Isala Clinics, Diabetes Centre<br>Navis, GJ; University Medical Center Groningen, Department of Nephrology<br>Denig, Petra; University Medical Center Groningen, Department of Clinical Pharmacy and Pharmacology

**Primary Subject Heading**:	Renal medicine
Secondary Subject Heading:	Health services research, Epidemiology, Pharmacology and therapeutics
Keywords:	secondary care, chronic kidney disease, prescribing quality, quality indicator, quality assessment

SCHOLARONE™
Manuscripts

PRESCRIBING QUALITY IN SECONDARY CARE PATIENTS WITH DIFFERENT STAGES OF CHRONIC KIDNEY DISEASE: A RETROSPECTIVE STUDY IN THE NETHERLANDS

Kirsten P.J. Smits[1]*, Grigory Sidorenkov[1], Frans J. van Ittersum[2], Femke Waanders[3], Henk J.G. Bilo[4], Gerjan J. Navis[5], Petra Denig[1]

[1] Department of Clinical Pharmacy and Pharmacology, University of Groningen, University Medical Center Groningen, the Netherlands
[2] Department of Nephrology, VU University Medical Center Amsterdam, Amsterdam, the Netherlands
[3] Department of Nephrology, Isala Clinics, Zwolle, the Netherlands
[4] Diabetes Centre, Isala Clinics, Zwolle, the Netherlands
[5] Department of Nephrology, University of Groningen, University Medical Center Groningen, Groningen, the Netherlands

* Corresponding author:
Dr. Kirsten P.J. Smits
Department of Quality and Safety of Oral Healthcare
Radboud university medical center
Philips van Leydenlaan 25, 6525 EX Nijmegen
Email: kirsten.smits@radboudumc.nl

Word count: 2895

For peer review only

For peer review only - http://bmjopen.bmj.com/site/about/guidelines.xhtml

ABSTRACT

Objectives: Insight in the prescribing quality for patients with chronic kidney disease (CKD) in secondary care is limited. The aim of this study is to assess the prescribing quality in secondary care patients with CKD stages 3-5, and assess possible differences in quality between CKD stages.

Design: This was a retrospective cohort study.

Setting: Data were collected at two university (n=569 and n=845) and one non-university nephrology outpatient clinic (n=1,718) in the Netherlands.

Participants: Between March 2015 and August 2016, data were collected from patients with stage 3a-5 CKD seen at the clinics. Blood pressure measurements, laboratory measurements and prescription data were extracted from medical records. For each prescribing quality indicator, patients with incomplete data required for calculation were excluded.

Outcome measures: Potentially appropriate prescribing of antihypertensives, renin-angiotensin-aldosterone-system (RAAS) inhibitors, statins, phosphate binders, and potentially inappropriate prescribing according to prevailing guidelines was assessed using prescribing quality indicators. Chi-square or Fisher's Exact tests were used to test for differences in prescribing quality.

Results: RAAS inhibitors alone or in combination with diuretics (57% respectively 52%) and statins (42%) were prescribed less often than phosphate binders (72%) or antihypertensives (94%) when indicated. Active vitamin D was relatively often prescribed when potentially not indicated (19%). Patients with high CKD stages were less likely to receive RAAS inhibitors but more likely to receive statins when indicated than stage 3 CKD patients. They also received more active vitamin D and erythropoietin stimulating agents when potentially not indicated.

For peer review only - http://bmjopen.bmj.com/site/about/guidelines.xhtml

For peer review only

Conclusions: Priority areas for improvement of prescribing in CKD outpatients include potential underprescribing of RAAS inhibitors and statins, and potential overprescribing of active vitamin D. CKD stage should be taken into account when assessing prescribing quality.

STRENGTHS AND LIMITATIONS OF THIS STUDY

- Quality of prescribing was assessed with validated quality indicators measuring both potential appropriate and inappropriate prescribing taking individual patient characteristics into account
- Quality of prescribing was compared between patients with different CKD stages and from different outpatient clinics
- Quality of prescribing was assessed in a cross-sectional manner, which disregards longitudinal aspects of care
- There were differences in patient population and data collection between the three outpatient clinics

Keywords: chronic kidney disease, secondary care, prescribing quality, quality indicator, quality assessment

For peer review only

INTRODUCTION

Assessing quality of care in chronic kidney disease patients (CKD) is important for identifying areas for improvement. Several recent studies have shown that detection of CKD, monitoring of disease progression and metabolic parameters, and achievement of risk factor target levels are suboptimal in CKD care.[1-4] Three of these studies showed that prescribing of selected medication treatment may also be suboptimal, for example, showing potential underprescribing of renin-angiotensin-aldosterone system (RAAS) inhibitors and statins, and overprescribing of non-steroidal anti-inflammatory drugs (NSAIDs) in primary care patients with CKD. In addition, one study showed an increasing trend in prescribing of RAAS inhibitors and statins in secondary CKD patients.[5] Not much is known about differences in prescribing quality in CKD patients between healthcare organizations.

Recently, our research group has developed a set of prescribing quality indicators (PQIs) for assessing the prescribing quality in patients with CKD according to clinical guideline recommendations, which has been validated in a primary care population.[6] The set is intended also for secondary care, and includes several indicators which are specifically relevant for patients with higher CKD stages. The PQIs give new insights in quality of prescribing in CKD patients, since they assess a broad range of prescribing issues at patient level and incorporate patient characteristics that are relevant for treatment decisions. Previously it was found that with increasing CKD stages prescribing of RAAS inhibitors and NSAIDs decreased, while prescribing of phosphate binders, vitamin D and erythropoietin-stimulating-agents (ESA) increased.[1] However, this was based on the number of prescriptions regardless of whether the medication was indicated for the included patients. The aim of this study is to assess prescribing quality in secondary care patients with CKD stage 3a-5 using PQIs that take relevant patient characteristics into account. Differences in prescribing quality between patients with different CKD stages were evaluated. In a secondary analysis, also differences between different outpatient clinics in the Netherlands were explored.

METHODS

For peer review only - http://bmjopen.bmj.com/site/about/guidelines.xhtml

This was a retrospective cross-sectional study assessing the prescribing quality between March 2015 and August 2016 in the Netherlands in two university nephrology outpatient clinics A and B, and one non-university nephrology outpatient clinic C. Included were patients with CKD stage 3a-5 based on estimated glomerular filtration rate (eGFR), i.e. stage 3a was defined as an eGFR ≥45 and <60 ml/min/1.73m2, stage 3b as an eGFR ≥30 and <45 ml/min/1.73m2, stage 4 as an eGFR ≥15 and <30 ml/min/1.73m2, and stage 5 as an eGFR <15 ml/min/1.73m2.[7] Patients who received dialysis or renal transplantation were excluded from the study.

The medical ethical committee of the University Medical Center Groningen ascertained that this study using anonymized medical record data does not fall under the Medical Research Involving Human Subjects Act.

Clinics and setting

Clinic A and clinic B are academic hospitals, which provided data from their general nephrology outpatient clinic and their predialysis outpatient clinic. Clinic C is a nonacademic, public hospital, which provided data from the general nephrology outpatient clinic. Twelve months of consecutive data were collected from June 1st 2015 to May 31st 2016 for clinic A, from March 1st 2015 to February 29th 2016 for clinic B and September 1st 2015 to August 31st 2016 for clinic C. In all three clinics, the included CKD patients commonly visit the outpatient clinics 2-4 times per year depending on the progression of their disease. At these visits, all medication can be reviewed and changed based on a medication list which is available for the nephrologist. In all clinics, the medication included in this study is usually prescribed by the nephrologist or nephrologist in training, although other specialists or the general practitioner may also prescribe medication during the year. Visits for the nephrology and the predialysis outpatient clinics are conducted in a similar way. All patients collect their medication at community pharmacies, where medication reviews may be conducted regardless of the outpatient clinic they visit. All patients have similar access to prescribed medication.

Prescribing quality

For peer review only - http://bmjopen.bmj.com/site/about/guidelines.xhtml

For peer review only

A previously developed set of patient-oriented PQIs was used for the assessment of prescribing quality of patients with CKD not undergoing renal replacement therapy.[6] This set was based on clinical guideline recommendations. The PQIs intend to provide insight in prescribing behaviour of physicians with regards to antihypertensives, RAAS inhibitors, statins and phosphate binders when recommended (potentially appropriate prescribing) as well as prescribing of dual RAAS blockade, active vitamin D, ESA, NSAIDs, metformin, and digoxin when considered not needed or unsafe (potentially inappropriate prescribing) (Table 1). To specify the indicators to specific needs of patients, most indicators focus on a subgroup of the population selected based on kidney function, risk factor levels and/or age as described in the clinical guidelines or added by the expert panel in the development phase. Since there will always be individual cases for whom this is not the case, we speak of 'potentially' appropriate (or inappropriate) prescribing. Antihypertensives include diuretics, beta blocking agents, calcium channel blockers, agents acting on the RAAS system and other antihypertensives such as centrally acting agents. Diuretics include all types of diuretics, e.g. low-ceiling and high-ceiling diuretics and potassium-sparing agents. RAAS inhibitors include angiotensin-converting-enzyme inhibitors and angiotensin-receptor-blockers. In the Netherlands, this medication is reimbursed by the health insurance after the patient's annual deductible excess has been exceeded. This is the same for all three clinics.

Table 1. Indicator definitions for the PQIs

Nr.	Indicator definition
Appropriate prescribing	
**1**	The percentage of patients between 18 and 80 years with CKD stages 4-5 and hypertension[†] that is prescribed antihypertensives unless undesirable because of low diastolic blood pressure (<70 mmHg)
**2**	The percentage of patients between 18 and 80 years with CKD stages 3-5 and proteinuria[‡] that is prescribed an ACE-i or ARB
**3**	The percentage of patients between 18 and 80 years with CKD stages 3-5, microalbuminuria[$] and diabetes[§] that is prescribed an ACE-I or ARB
**4**	The percentage of patients between 18 and 80 years with CKD stages 3-5 and proteinuria[‡] treated with multiple antihypertensives that is prescribed a combination of an ACE-i or ARB and a diuretic
**5**	The percentage of patients between 18 and 80 years with CKD stages 3-5, microalbuminuria[$] and diabetes[§] treated with multiple antihypertensives that is prescribed a combination of an ACE-I or ARB and a diuretic.

For peer review only - http://bmjopen.bmj.com/site/about/guidelines.xhtml

**6**	The percentage of patients between 50 and 65 years with CKD stages 3-5 that is prescribed a statin
**7**	The percentage of patients between 18 and 80 years with CKD stages 3-5 and an elevated phosphate level (>1.49 mmol/l) that is prescribed a phosphate binder
**8**	The percentage of patients between 18 and 80 years with CKD stages 3-5 treated with phosphate binders and with an elevated calcium level (>2.54 mmol/l) that is prescribed a non-calcium-containing phosphate binder
**9**	The percentage of patients between 18 and 80 years with CKD stages 3-5 treated with phosphate binders and with a low calcium level (<2.10 mmol/l) that is prescribed a calcium-containing phosphate binder
Inappropriate prescribing	
**10**	The percentage of patients 18 years or older with CKD stages 3-5 treated with RAAS inhibitors that is prescribed at least two RAAS inhibitors simultaneously (dual RAAS blockade)
**11**	The percentage of patients 18 years or older with CKD stages 3-5 and an elevated calcium level (>2.54 mmol/l) that is prescribed active vitamin D
**12**	The percentage of patients 18 years or older with CKD stages 3-5 and a normal haemoglobin level (≥7.5 mmol/l) that is prescribed an ESA
**13**	The percentage of patients 18 years or older with eGFR < 30 ml/min/1.73 m^2 that is prescribed an NSAID
**14**	The percentage of patients 18 years or older with eGFR < 30 ml/min/1.73 m^2 and diabetes[§] that is prescribed metformin
**15**	The percentage of patients 18 years or older with eGFR < 50 ml/min/1.73 m^2 treated with digoxin that is prescribed high dose digoxin (>0.125 mg/day)
**16**	The percentage of patients 18 years or older with CKD stages 3-5 that is prescribed a combination of NSAIDs, RAAS inhibitors and diuretics

CKD: chronic kidney disease; ACE-i: angiotensin-converting-enzyme inhibitor; ARB: angiotensin-receptor-blocker; RAAS: renin-angiotensin-aldosterone-system; ESA: erythropoietin-stimulating agent; eGFR: estimated glomerular filtration rate; NSAIDs: non-steroidal anti-inflammatory drugs.

† Hypertension is defined as having a systolic blood pressure > 140 mmHg or being prescribed antihypertensives. $ Micro-albuminuria is defined as albumin/creatinine ratio ≥3.0 mg/mmol and <30 mg/mmol. ‡ Proteinuria is defined as > 0.5 g protein per 24h or l urine or albumin/creatinine ratio ≥30 mg/mmol. § diabetes is defined as either the diagnosis for diabetes or being prescribed with glucose lowering drugs.

Data collection

For each patient with at least one visit to a nephrologist within the study period, blood pressure measurement, laboratory measurements and prescription data of the most recent visit were extracted from the medical records, either by manual (clinic A) or computerized (clinic B and C) extraction routines. Age was determined on the visit day. For some patients, the visit date was unknown, in which case the most recent date of the eGFR assessment was used as a proxy for the visit date. The eGFR was calculated from serum creatinine using the Modification of Diet in Renal Disease (MDRD) formula.[8] If serum creatinine was not available, the reported eGFR calculated with

For peer review only

For peer review only - http://bmjopen.bmj.com/site/about/guidelines.xhtml

the MDRD formula was used. Proteinuria was defined as more than 0.5 g of protein in 24 hour or per liter urine, depending on availability.

Patient and Public involvement

Patients or public were not directly involved in this study. Three patient representatives were, however, involved in the development phase of the prescribing quality indicators applied in this study to ascertain that all relevant topics from the patient perspective were included [6].

Statistical analysis

Means with standard deviations (SD) are reported for normally distributed continuous variables, medians with interquartile ranges for non-normally distributed variables, and percentages for categorical variables. The PQI scores are presented as percentages with 95% confidence intervals (CI). Chi-square tests or Fisher's Exact tests, in case of cell frequencies below 5, were used to test for differences in prescribing quality across different CKD stages and different clinics. P-values <0.05 were considered statistically significant. When comparing individual PQIs between CKD stages or clinics, Bonferroni correction for multiple testing was applied. Analyses were conducted using Stata version 14.2 Special Edition (Stata Corp., College Station, TX).

RESULTS

In total, 3,132 patients with CKD stage 3a-5 were included in this study. Included patients were on average 68 years (SD: 14) old, 56% were males, the median eGFR was 35 ml/min/1.73m2 (interquartile range: 24-46) and 16% had diabetes. Patients with higher CKD stages more often had blood pressure and laboratory measurements (Table 2).

For peer review only

Table 2. Patient characteristics for the whole population and separate per CKD stage

	Overall (n=3,132)		CKD 3a (n=843)		CKD 3b (n=1,125)		CKD 4 (n=862)		CKD 5 (n=302)		Chi^2
	N (%)	Mean (±SD)	N (%)	Mean (±SD)	N (%)	Mean (±SD)	N (%)	Mean (±SD)	N (%)	Mean (±SD)	p-value
Age (years)		67.7 (±14.1)		63.1 (±14.2)		68.9 (±13.2)		70.2 (±13.8)		69.2 (±14.8)	
< 50 years	363 (11.6)		151 (17.9)		113 (10.0)		70 (8.1)		29 (9.6)		
50-80 years	2,149 (68.6)		604 (71.7)		793 (70.5)		556 (64.5)		196 (64.9)		
>= 80 years	620 (19.8)		88 (10.4)		219 (19.5)		236 (27.4)		77 (25.5)		
Gender (males)	1,738 (55.5)		456 (54.1)		614 (54.6)		488 (56.6)		180 (59.6)		0.314
Diabetes (yes)	485 (15.5)		96 (11.4)		166 (14.8)		165 (19.1)		58 (19.2)		<0.001
eGFR (MDRD) (ml/min/1.73m^2	3,132 (100)	35 [24-46][a]	843 (100)	52.2 (±4.3)	1,125 (100)	37.3 (±4.2)	862 (100)	23.1 (±4.3)	302 (100)	11.1 (±2.6)	
SBP (mmHg)	2,511 (80.2)	132.0 (±18.8)	601 (71.3)	129.4 (±17.3)	910 (80.9)	130.7 (±18.6)	735 (85.3)	133.3 (±19.4)	265 (87.7)	139.1 (±19.2)	<0.001
Elevated SBP (>140 mmHg)	700 (22.3)	155.3 (±12.4)	119 (14.1)	155.3 (±12.3)	239 (21.2)	154.3 (±12.5)	233 (27.0)	155.4 (±12.2)	109 (36.1)	157.1 (±12.6)	
DBP (mmHg)	2,511 (80.2)	75.1 (±11.2)	601 (71.3)	76.7 (±10.6)	910 (80.9)	74.9 (±11.3)	735 (85.3)	74.6 (±11.2)	265 (87.7)	74.0 (±12.1)	<0.001
Low DBP (<70 mmHg)	704 (22.5)	61.6 (±5.8)	130 (15.4)	62.1 (±5.5)	282 (25.1)	62.1 (±5.4)	214 (24.8)	61.3 (±6.1)	78 (25.8)	59.4 (±6.5)	
Total protein (g/24h urine)	1,314 (42.0)	0.4 [0.1-1.3][a]	278 (33.0)	0.3 [0.1-0.8][a]	426 (37.9)	0.2 [0.1-0.8][a]	403 (46.8)	0.4 [0.2-1.3][a]	207 (68.5)	1.3 [0.5-2.8][a]	<0.001
Total protein (g/L urine)	2,328 (74.3)	0.2 [0.1-0.6][a]	552 (65.5)	0.1 [0.1-0.3][a]	841 (74.8)	0.2 [0.1-0.4][a]	687 (79.7)	0.3 [0.1-0.6][a]	248 (82.1)	0.8 [0.3-1.7][a]	<0.001
Proteinuria (>0.5 g/24h or L urine)	810 (25.9)		140 (16.6)		224 (19.9)		268 (31.1)		178 (58.9)		
Phosphate (mmol/l)	2,606 (83.2)	1.08 (±0.29)	539 (63.9)	0.96 (±0.20)	959 (85.2)	1.00 (±0.20)	815 (94.5)	1.10 (±0.25)	293 (97.0)	1.48 (±0.39)	<0.001
Elevated phosphate (>1.49 mmol/l)	172 (5.5)	1.80 (±0.34)	3 (0.4)	1.69 (±0.22)	17 (1.5)	1.65 (±0.20)	41 (4.8)	1.72 (±0.32)	111 (36.8)	1.85 (±0.36)	
Calcium (mmol/l)	2,734 (87.3)	2.36 (±0.14)	616 (73.1)	2.38 (±0.11)	998 (88.7)	2.37 (±0.13)	822 (95.4)	2.35 (±0.15)	298 (98.7)	2.30 (±0.16)	<0.001
Elevated Calcium (>2.54 mmol/l)	163 (5.2)	2.62 (±0.08)	40 (4.7)	2.61 (±0.08)	61 (5.4)	2.62 (±0.09)	46 (5.3)	2.62 (±0.06)	16 (5.3)	2.64 (±0.08)	
Haemoglobin (mmol/l)	3,024 (96.6)	8.0 (±1.1)	771 (91.5)	8.5 (±1.1)	1,095 (97.3)	8.2 (±1.0)	857 (99.4)	7.7 (±1.1)	301 (99.7)	7.0 (±0.9)	<0.001
Low haemoglobin level (<7.5 mmol/l)	933 (29.8)	6.7 (±0.6)	113 (13.4)	6.8 (±0.6)	264 (23.5)	6.8 (±0.5)	346 (40.1)	6.7 (±0.6)	210 (69.5)	6.6 (±0.7)	
Clinic											<0.001
A	569 (18.2)		92 (10.9)		166 (14.8)		183 (21.2)		128 (42.4)		
B	845 (27.0)		255 (30.3)		295 (26.2)		219 (25.4)		76 (25.2)		
C	1,718 (54.9)		496 (58.8)		664 (59.0)		460 (53.4)		98 (32.5)		

CKD: chronic kidney disease; eGFR: estimated glomerular filtration rate; MDRD: Modification of Diet in Renal Disease; SBP: systolic blood pressure; DBP: diastolic blood pressure. Clinic A and B: university nephrology outpatient clinics; clinic C: non-university nephrology outpatient clinic.

[a] Median with interquartile range.

For peer review only - http://bmjopen.bmj.com/site/about/guidelines.xhtml

Overall prescribing quality

Two PQIs focusing on prescribing of RAAS inhibitors in patients with micro-albuminuria and diabetes were not operational valid in this population because the limited availability of albumin/creatinin ratios (indicators 3 and 5). Furthermore, two PQIs focusing on prescribing of non-calcium-containing and calcium-containing phosphate binders in patients with respectively high and low calcium levels were not operational valid because of the low inclusion of eligible patients (indicators 8 and 9). These four PQIs were excluded from further analysis.

Potentially appropriate prescribing rates varied from 94% of patients receiving antihypertensives, 57% and 52% receiving RAAS inhibitors alone or in combination with a diuretic, 42% receiving statins, and 72% receiving phosphate binders when indicated according to the guideline (Fig 1). Potentially inappropriate prescribing rates varied from 19% of patients receiving active vitamin D, 3% receiving ESA, 1% receiving NSAIDs, 3% receiving metformin and 4% receiving high-dose digoxin when this was possibly not needed or unsafe.

Prescribing quality across chronic kidney disease stages

Potential appropriate prescribing of RAAS inhibitors alone occurred significantly less in patients with CKD stage 5 compared to all other stages, which was also true for the combination of RAAS inhibitors and diuretics (Fig 2). Patients with stage 3a were less likely to receive recommended treatment with statins than patients with stage 4 or 5. Similarly, patients with stage 3b were less likely to receive statins compared to patients with stage 4. Potential inappropriate prescribing of active vitamin D in patients with elevated calcium occurred significantly less in patients with stages 3a and 3b compared to patients with stages 4 and 5. This was also the case for potentially inappropriate prescribing of ESA. Finally, potentially inappropriate prescribing of metformin in patients with an eGFR < 30 ml/min/1.73 m2 was significantly lower for stage 5 as compared to stage 4.

Prescribing quality across nephrology outpatient clinics

For peer review only - http://bmjopen.bmj.com/site/about/guidelines.xhtml

Patients visiting the university outpatient clinics A and B were on average younger (63 years (SD: 15) and 65 years (SD: 15)) compared to those visiting the non-university outpatient clinic C (71 years (SD: 13)). Furthermore, patients visiting clinic A more often had CKD stage 4 or 5 compared to patients from clinics B and C (Table 2). The diabetes prevalence was higher at clinic A (26%) compared to clinic B (19%) and clinic C (10%) (Supplementary table 1).

Significant differences were seen between clinic A and clinic C in potentially appropriate prescribing of antihypertensives, RAAS inhibitors alone, statins, and in potentially inappropriate prescribing of metformin as well as the combination of NSAIDs, RAAS inhibitors and diuretics (Fig 3). Furthermore, significantly more potentially appropriate prescribing of phosphate binders was seen in clinic A as compared to clinic B.

In the analyses per CKD stage (Supplementary tables 2 and 3), similar differences were found between the clinics. In patients with stage 3a CKD, potentially appropriate prescribing of RAAS inhibitors combined with diuretics occurred the least in clinic B. In patients with stage 3b CKD, potential appropriate prescribing of RAAS inhibitors alone or combined with diuretics occurred the most in clinic A. Patients with stage 4 CKD were significantly more likely in clinic A as compared to clinic C to receive antihypertensives and RAAS inhibitors alone. Also, patients with CKD stage 5 were more likely in clinic A as compared to clinic C to receive phosphate binders when indicated.

DISCUSSION

This is a first study to assess the prescribing quality in secondary care CKD patients using a broad set of patient-oriented PQIs and comparing patients with different CKD stages and from different outpatient clinics. The results show that the prescribing quality seem to vary between therapeutic areas. RAAS inhibitors and statins were prescribed in less than 60% of the patients for whom this is potentially indicated, whereas prescribing rates for antihypertensives and phosphate binders when potentially indicated were much higher. Active vitamin D was prescribed in almost one fifth of all patients while considered potentially not needed or unsafe. The prescribing levels also varied across

For peer review only - http://bmjopen.bmj.com/site/about/guidelines.xhtml

For peer review only

different CKD stages, with decreasing prescribing of RAAS inhibitors, increasing prescribing of statins, and increasing prescribing of active vitamin D and ESA with higher CKD stages. Finally, significant differences were observed in prescribing between the different outpatient clinics, also after stratification for CKD stage.

Previous studies looking at the overall volume of prescribing suggested that there was underprescribing of RAAS inhibitors and statins[1, 5] and overprescribing of NSAIDs[1] in patients with CKD. In addition, it was shown that patients with higher CKD stages receive more treatment with antihypertensives, phosphate binders, vitamin D and ESA than patients with lower CKD stages.[1, 9] On the other hand, RAAS inhibitors and NSAIDs were less prescribed with increasing CKD stages.[1] These studies, however, did not take the specific indications for treatment into account. Our study using validated PQIs, which assess prescribing in patients for whom this is indicated, confirmed that potential underprescribing of RAAS inhibitors and statins may be areas for possible improvement in CKD care. This was also found in a recent study among patients with stage 3-4 CKD in Canada.[3] In some patients with CKD stage 5 who are in preparation of dialysis or transplantation, RAAS inhibitors may be deliberately stopped to retain the residual kidney function.[10] Lower statin prescribing rates in patients with lower CKD stages suggests that prescribers may be less aware or convinced of the need to prescribe statins in these patients. A similar pattern of less statin prescribing in patients with a higher eGFR compared to lower eGFR was observed for the elderly primary care patients in Canada.[11] Regarding potentially inappropriate prescribing, the Canadian primary care study observed a relatively high prescribing rate of NSAIDs (16%) and low prescribing rate of dual RAAS blockade (3%). Our study showed that potentially unsafe prescribing of both NSAIDs and dual RAAS blockade was uncommon in secondary care patients managed in the Netherlands.

Although the applied PQIs are patient-oriented, taking relevant patient characteristics into account, they reflect general guideline recommendations, and therefore a perfect score is never pursued.

For peer review only - http://bmjopen.bmj.com/site/about/guidelines.xhtml

There can be valid reasons to refrain from prescribing according to guideline recommendations in certain patients. Valid reasons include lack of response to certain drugs, drug intolerances or patient preferences for or against certain treatment. For example, hyperkalaemia could be a reason to stop treatment with RAAS inhibitors. The assessment using PQIs is therefore useful to provide insight and monitor the quality of care at population level and not to assess the quality of treatment for each individual patient. Furthermore, it has been argued that patient case-mix including difference in aspects, such as age or comorbidities, may explain differences in quality scores.[12] However, these may not necessarily be valid reasons for not complying with guideline recommendations. When developing the PQIs, such differences are to some extent included in the indicator definitions (e.g. age limits), thereby ensuring that the treatments are in general either recommended or inappropriate in the patients included in the indicator. Furthermore, a recent review showed that unjustified case-mix corrections can mask actual differences in quality of care.[13] Therefore, no case-mix adjustment was made when applying the PQIs.

This study assessed the prescribing quality in a cross-sectional manner, since the PQIs were defined as cross-sectional measures. This may lead to including patients who reached abnormal risk factor levels for the first time. In some cases, the healthcare provider may decide to postpone the start of treatment until a next measurement to make sure that the abnormal risk factor level persists. This also holds for discontinuation of active vitamin D in patients with elevated calcium levels and ESA in patients with normal haemoglobin levels. Furthermore, it is possible that the laboratory results became available after the visit. Therefore, the healthcare provider may not have been aware at the time of the visit that they should start or discontinue medication. In the diabetes field, it has been proposed that indicators using multiple time points may give a more accurate assessment of the prescribing quality.[14, 15] Such indicators assess whether the healthcare providers start or intensify treatment when patients do not return to normal risk factor levels.

For peer review only - http://bmjopen.bmj.com/site/about/guidelines.xhtml

Differences between clinics may in part be due to differences in the underlying patient population, such as the age and comorbidity. All PQIs focusing on recommended treatment with antihypertensives, RAAS inhibitors, statins and phosphate binders, however, have an age limit which excludes the older, more frail patients. Diabetes prevalence was higher in clinic A, which may have affected prescribing. Other studies indicate that CKD patients with diabetes receive better quality of care in general,[16] and have higher prescription rates of RAAS inhibitors and statins.[3] There were also differences in data collection methods between the clinics. Although we were able to extract and combine the data in order to make comparisons possible, there were some differences in availability of measurement values. Furthermore, data from the blood pressure and laboratory measurements were sometimes missing (table 2), with the highest rate of missingness for clinic B (Supplementary table 2). This could have influenced the outcome of the PQIs, since patients with unknown values were not included in the PQIs. We can only speculate why these values were missing, and how this may have influenced the assessments. It could be that the blood pressure and laboratory measurements were not performed, not recorded or lost during data extraction. Our aim was not to explain differences in prescribing. To understand the causes underlying prescribing variation, future studies should look at the influence of patient, prescriber and organisation characteristics.

In conclusion, using a novel set of PQIs assessing prescribing quality with patient level data we successfully identified several areas for potential improvement. This included potential underprescribing of RAAS inhibitors and statins and potential overprescribing of active vitamin D in secondary care patients with CKD stages 3-5. This information can support clinicians to identify the patients that are truly in need of improved treatment. We observed differences in prescribing quality between the CKD stages and between the outpatient clinics. We conclude that monitoring of the prescribing quality with PQIs in secondary care and stratification on CKD stage can be used to identify priority areas for quality improvement initiatives.

For peer review only

ACKNOWLEDGEMENTS

We would like to thank the following people for the data collection: Judith Bos (Clinic A), Thijme Last (Clinic B) and Bert Dikkeschei and Erwin Booij (Clinic C).

COMPETING INTERESTS

None declared.

AUTHOR CONTRIBUTIONS

KSmits: Research idea; study design; planning; data acquisition; data analysis and interpretation; GSidorenkov: Research idea; study design; data analysis and interpretation; supervision or mentorship; FvanIttersum: Data acquisition; data interpretation; FWaanders: Data acquisition; data interpretation; HBilo: Research idea; study design; data acquisition; data analysis and interpretation; supervision or mentorship; GNavis: Research idea; study design; data acquisition; data analysis and interpretation; supervision or mentorship; PDenig: Research idea; study design; data acquisition; data analysis and interpretation; supervision or mentorship. Each author contributed important intellectual content during manuscript drafting or revisions. All authors approved the manuscript and this submission.

FUNDING

This work was supported by ZonMW, the Netherlands Organisation for Health Research and Development, grant number 836021013. The funder had no role in study design, data collection and analysis, decision to publish, or preparation of the manuscript.

DATA SHARING STATEMENT

Clinical data as collected from the outpatient clinics cannot be made publicly available according to Dutch privacy regulations. Datasets are only available for data integrity auditing procedures.

For peer review only - http://bmjopen.bmj.com/site/about/guidelines.xhtml

REFERENCES

1. Arora P, Elkin PL, Eberle J, Bono JJ, Argauer L, Murray BM, et al. An observational study of the quality of care for chronic kidney disease: a Buffalo and Albany, New York metropolitan area study. BMC Nephrol. 2015;16:199.
2. Jolly SE, Navaneethan SD, Schold JD, Arrigain S, Sharp JW, Jain AK, et al. Chronic kidney disease in an electronic health record problem list: quality of care, ESRD, and mortality. Am J Nephrol. 2014;39(4):288-96.
3. Manns L, Scott-Douglas N, Tonelli M, Weaver R, Tam-Tham H, Chong C, et al. A Population-Based Analysis of Quality Indicators in CKD. Clin J Am Soc Nephrol. 2017;12(5):727-33.
4. Van Gelder VA, Scherpbier-De Haan ND, De Grauw WJ, Vervoort GM, Van Weel C, Biermans MC, et al. Quality of chronic kidney disease management in primary care: a retrospective study. Scand J Prim Health Care. 2016;34(1):73-80.
5. Ang GY, Heng BH, Liew AS, Chong PN. Quality of care of patients with chronic kidney disease in national healthcare group polyclinics from 2007 to 2011. Ann Acad Med Singapore. 2013;42(12):632-9.
6. Smits KP, Sidorenkov G, Bilo HJ, Bouma M, van Ittersum FJ, Voorham J, et al. Development and initial validation of prescribing quality indicators for patients with chronic kidney disease. Nephrol Dial Transplant. 2016;31(11):1876-86.
7. Kidney Disease: Improving Global Outcomes (KDIGO) CKD Work Group. KDIGO 2012 Clinical Practice Guideline for the Evaluation and Management of Chronic Kidney Disease. Kidney Inter, Suppl. 2013;3(1):S1-150.
8. Levey AS, Bosch JP, Lewis JB, Greene T, Rogers N, Roth D. A more accurate method to estimate glomerular filtration rate from serum creatinine: a new prediction equation. Modification of Diet in Renal Disease Study Group. Ann Intern Med. 1999;130(6):461-70.
9. Jameson K, Jick S, Hagberg KW, Ambegaonkar B, Giles A, O'Donoghue D. Prevalence and management of chronic kidney disease in primary care patients in the UK. Int J Clin Pract. 2014;68(9):1110-21.
10. Ahmed AK, Kamath NS, El Kossi M, El Nahas AM. The impact of stopping inhibitors of the renin-angiotensin system in patients with advanced chronic kidney disease. Nephrol Dial Transplant. 2010;25(12):3977-82.
11. Nash DM, Brimble S, Markle-Reid M, McArthur E, Tu K, Nesrallah GE, et al. Quality of Care for Patients With Chronic Kidney Disease in the Primary Care Setting: A Retrospective Cohort Study From Ontario, Canada. Can J Kidney Health Dis. 2017;4:1-14.
12. Lilford R, Mohammed MA, Spiegelhalter D, Thomson R. Use and misuse of process and outcome data in managing performance of acute medical care: avoiding institutional stigma. Lancet. 2004;363(9415):1147-54.
13. Calsbeek H, Markhorst JG, Voerman GE, Braspenning JC. Case-mix adjustment for diabetes indicators: a systematic review. Am J Manag Care. 2016;22(2):e45-52.
14. Voorham J, Denig P, Wolffenbuttel BH, Haaijer-Ruskamp FM. Cross-sectional versus sequential quality indicators of risk factor management in patients with type 2 diabetes. Med Care. 2008;46(2):133-41.
15. Aron DC. Quality indicators and performance measures in diabetes care. Curr Diab Rep. 2014;14(3):472.
16. van Dipten C, van Berkel S, van Gelder VA, Wetzels JFM, Akkermans RP, de Grauw WJC, et al. Adherence to chronic kidney disease guidelines in primary care patients is associated with comorbidity. Fam Pract. 2017;34(4):459-66.

For peer review only - http://bmjopen.bmj.com/site/about/guidelines.xhtml

FIGURE LEGENDS

Fig 1. Overall prescribing quality assessed with 5 PQIs for appropriate prescribing (Ind 1-7) and 7 PQIs for potential inappropriate prescribing (Ind 10-16). Ind 1: Patients with hypertension prescribed antihypertensives; Ind 2: Patients with albuminuria prescribed renin-angiotensin-aldosterone system (RAAS) inhibitors; Ind 4: Patients on multiple antihypertensives prescribed a combination of RAAS inhibitors and diuretics; Ind 6: Patients aged 50 to 65 years prescribed statins; Ind 7: Patients with high phosphate levels prescribed phosphate binders; Ind 10: Patients prescribed dual RAAS blockade; Ind 11: Patients with high calcium levels prescribed active vitamin D; Ind 12: Patients with normal haemoglobin levels prescribed erythropoietin stimulating agents; Ind 13: Patients with an estimated glomerular filtration rate (eGFR) lower than 30 ml/min/1.73m^2 prescribed high dose non-steroidal anti-inflammatory drugs (NSAIDs); Ind 14: Patients with diabetes and an eGFR lower than 30 ml/min/1.73m^2 prescribed metformin; Ind 15: Patients with an eGFR lower than 50 ml/min/1.73m^2 prescribed high dose digoxin; Ind 16: Patients prescribed a combination of NSAIDs, RAAS inhibitors and diuretics. 95% CIs were calculated based on included number of patients in the denominator of each indicator.

Fig 2. Prescribing quality across different CKD stages (3A-5) assessed with 5 PQIs for appropriate prescribing (Ind 1-7) and 7 PQIs for potential inappropriate prescribing (Ind 10-16). Ind 1: Patients with hypertension prescribed antihypertensives; Ind 2: Patients with albuminuria prescribed renin-angiotensin-aldosterone system (RAAS) inhibitors; Ind 4: Patients on multiple antihypertensives prescribed a combination of RAAS inhibitors and diuretics; Ind 6: Patients aged 50 to 65 years prescribed statins; Ind 7: Patients with high phosphate levels prescribed phosphate binders; Ind 10: Patients prescribed dual RAAS blockade; Ind 11: Patients with high calcium levels prescribed active vitamin D; Ind 12: Patients with normal haemoglobin levels prescribed erythropoietin stimulating agents; Ind 13: Patients with an estimated glomerular filtration rate (eGFR) lower than 30 ml/min/1.73m^2 prescribed high dose non-steroidal anti-inflammatory drugs (NSAIDs); Ind 14: Patients with diabetes and an eGFR lower than 30 ml/min/1.73m^2 prescribed metformin; Ind 15: Patients with an eGFR lower than 50 ml/min/1.73m^2 prescribed high dose digoxin; Ind 16: Patients prescribed a combination of NSAIDs, RAAS inhibitors and diuretics. 95% CIs were calculated based on included number of patients in the denominator of each indicator. * Significant difference between 2 or more CKD stages using Chi-square or Fisher's exact test with Bonferroni-correction for multiple testing.

Fig 3: Prescribing quality across different outpatient clinics. Clinic A and B: university nephrology outpatient clinics; clinic C: non-university nephrology outpatient clinic. Ind 1: Patients with hypertension prescribed antihypertensives; Ind 2: Patients with albuminuria prescribed renin-angiotensin-aldosterone system (RAAS) inhibitors; Ind 4: Patients on multiple antihypertensives prescribed a combination of RAAS inhibitors and diuretics; Ind 6: Patients aged 50 to 65 years prescribed statins; Ind 7: Patients with high phosphate levels prescribed phosphate binders; Ind 10: Patients prescribed dual RAAS blockade; Ind 11: Patients with high calcium levels prescribed active vitamin D; Ind 12: Patients with normal haemoglobin levels prescribed erythropoietin stimulating agents; Ind 13: Patients with an estimated glomerular filtration rate (eGFR) lower than 30 ml/min/1.73m^2 prescribed high dose non-steroidal anti-inflammatory drugs (NSAIDs); Ind 14: Patients with diabetes and an eGFR lower than 30 ml/min/1.73m^2 prescribed metformin; Ind 15: Patients with an eGFR lower than 50 ml/min/1.73m^2 prescribed high dose digoxin; Ind 16: Patients

For peer review only - http://bmjopen.bmj.com/site/about/guidelines.xhtml

prescribed a combination of NSAIDs, RAAS inhibitors and diuretics. 95% CIs were calculated based on included number of patients in the denominator of each indicator. * Significant difference between 2 or all outpatient clinics using Chi-square or Fisher's exact test with Bonferroni-correction for multiple testing.

SUPPORTING INFORMATION

Supplementary table 1. Baseline characteristics per nephrology outpatient clinic. eGFR: estimated glomerular filtration rate; MDRD: Modification of Diet in Renal Disease; SBP: systolic blood pressure; DBP: diastolic blood pressure. Clinic A and B: university nephrology outpatient clinics; clinic C: non-university nephrology outpatient clinic. [a] Median with interquartile range.

Supplementary table 2. Baseline table per chronic kidney disease stage per location. CKD: chronic kidney disease; SD: standard deviation; eGFR: estimated glomerular filtration rate; MDRD: Modification of Diet in Renal Disease; SBP: systolic blood pressure; DBP: diastolic blood pressure. Clinic A and B: university nephrology outpatient clinics; clinic C: non-university nephrology outpatient clinic. [a] Median with interquartile range.

Supplementary table 3. Differences between nephrology outpatient clinics per indicator stratified for chronic kidney disease stage. CKD: chronic kidney disease; RAAS: renin-angiotensin-aldosterone system; ESA: erythropoiesis-stimulating agent; NSAID: non-steroidal anti-inflammatory drug. Clinic A and B: university nephrology outpatient clinics; clinic C: non-university nephrology outpatient clinic. † Fisher's exact because of <5 expected number of patients in one or more cells.

For peer review only

For peer review only - http://bmjopen.bmj.com/site/about/guidelines.xhtml

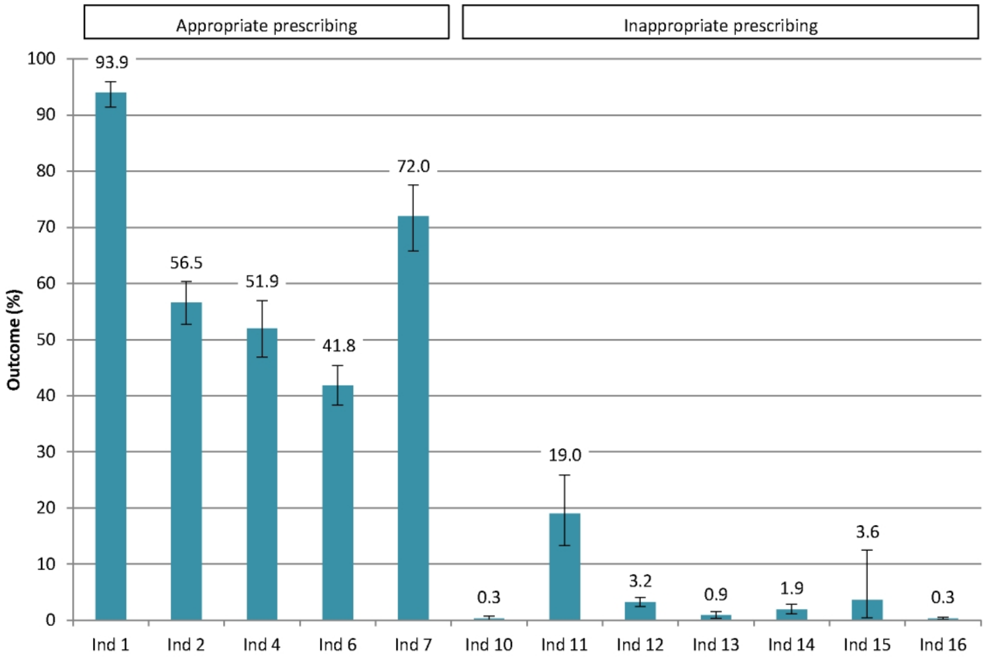

Fig 1. Overall prescribing quality assessed with 5 PQIs for appropriate prescribing (Ind 1-7) and 7 PQIs for potential inappropriate prescribing (Ind 10-16). Ind 1: Patients with hypertension prescribed antihypertensives; Ind 2: Patients with albuminuria prescribed renin-angiotensin-aldosterone system (RAAS) inhibitors; Ind 4: Patients on multiple antihypertensives prescribed a combination of RAAS inhibitors and diuretics; Ind 6: Patients aged 50 to 65 years prescribed statins; Ind 7: Patients with high phosphate levels prescribed phosphate binders; Ind 10: Patients prescribed dual RAAS blockade; Ind 11: Patients with high calcium levels prescribed active vitamin D; Ind 12: Patients with normal haemoglobin levels prescribed erythropoietin stimulating agents; Ind 13: Patients with an estimated glomerular filtration rate (eGFR) lower than 30 ml/min/1.73m2 prescribed high dose non-steroidal anti-inflammatory drugs (NSAIDs); Ind 14: Patients with diabetes and an eGFR lower than 30 ml/min/1.73m2 prescribed metformin; Ind 15: Patients with an eGFR lower than 50 ml/min/1.73m2 prescribed high dose digoxin; Ind 16: Patients prescribed a combination of NSAIDs, RAAS inhibitors and diuretics. 95% CIs were calculated based on included number of patients in the denominator of each indicator.

150x100mm (300 x 300 DPI)

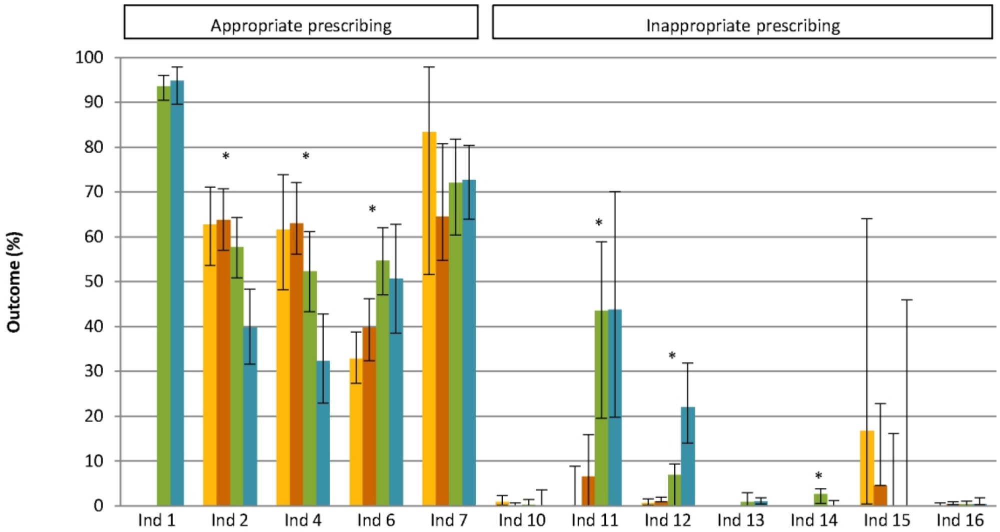

	Ind 1	Ind 2	Ind 4	Ind 6	Ind 7	Ind 10	Ind 11	Ind 12	Ind 13	Ind 14	Ind 15	Ind 16
Stage 3A		62.7	61.7	32.9	83.3	0.8	0.0	0.6			16.7	0.1
Stage 3B		63.8	63.0	39.7	64.5	0.0	6.6	1.0			4.5	0.4
Stage 4	93.6	57.7	52.3	54.6	72.0	0.2	43.5	6.8	0.8	2.6	0.0	0.3
Stage 5	94.8	39.7	32.3	50.7	72.7	0.0	43.8	22.0	1.0	0.0	0.0	0.3

Fig 2. Prescribing quality across different CKD stages (3A-5) assessed with 5 PQIs for appropriate prescribing (Ind 1-7) and 7 PQIs for potential inappropriate prescribing (Ind 10-16). Ind 1: Patients with hypertension prescribed antihypertensives; Ind 2: Patients with albuminuria prescribed renin-angiotensin-aldosterone system (RAAS) inhibitors; Ind 4: Patients on multiple antihypertensives prescribed a combination of RAAS inhibitors and diuretics; Ind 6: Patients aged 50 to 65 years prescribed statins; Ind 7: Patients with high phosphate levels prescribed phosphate binders; Ind 10: Patients prescribed dual RAAS blockade; Ind 11: Patients with high calcium levels prescribed active vitamin D; Ind 12: Patients with normal haemoglobin levels prescribed erythropoietin stimulating agents; Ind 13: Patients with an estimated glomerular filtration rate (eGFR) lower than 30 ml/min/1.73m2 prescribed high dose non-steroidal anti-inflammatory drugs (NSAIDs); Ind 14: Patients with diabetes and an eGFR lower than 30 ml/min/1.73m2 prescribed metformin; Ind 15: Patients with an eGFR lower than 50 ml/min/1.73m2 prescribed high dose digoxin; Ind 16: Patients prescribed a combination of NSAIDs, RAAS inhibitors and diuretics. 95% CIs were calculated based on included number of patients in the denominator of each indicator. * Significant difference between 2 or more CKD stages using Chi-square or Fisher’s exact test with Bonferroni-correction for multiple testing.

150x100mm (300 x 300 DPI)

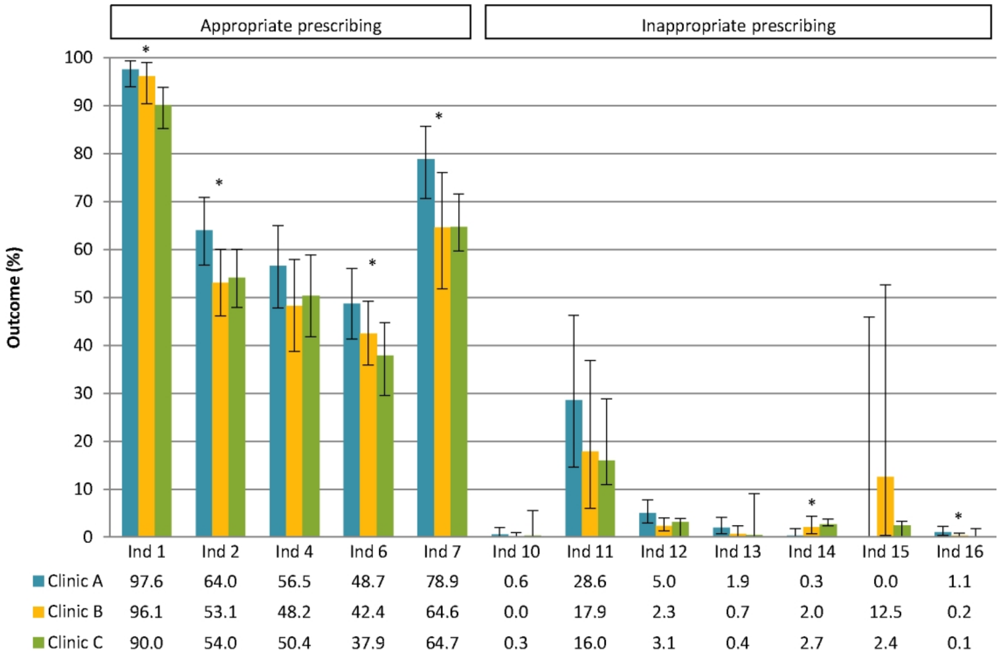

Fig 3: Prescribing quality across different outpatient clinics. Clinic A and B: university nephrology outpatient clinics; clinic C: non-university nephrology outpatient clinic. Ind 1: Patients with hypertension prescribed antihypertensives; Ind 2: Patients with albuminuria prescribed renin-angiotensin-aldosterone system (RAAS) inhibitors; Ind 4: Patients on multiple antihypertensives prescribed a combination of RAAS inhibitors and diuretics; Ind 6: Patients aged 50 to 65 years prescribed statins; Ind 7: Patients with high phosphate levels prescribed phosphate binders; Ind 10: Patients prescribed dual RAAS blockade; Ind 11: Patients with high calcium levels prescribed active vitamin D; Ind 12: Patients with normal haemoglobin levels prescribed erythropoietin stimulating agents; Ind 13: Patients with an estimated glomerular filtration rate (eGFR) lower than 30 ml/min/1.73m2 prescribed high dose non-steroidal anti-inflammatory drugs (NSAIDs); Ind 14: Patients with diabetes and an eGFR lower than 30 ml/min/1.73m2 prescribed metformin; Ind 15: Patients with an eGFR lower than 50 ml/min/1.73m2 prescribed high dose digoxin; Ind 16: Patients prescribed a combination of NSAIDs, RAAS inhibitors and diuretics. 95% CIs were calculated based on included number of patients in the denominator of each indicator. * Significant difference between 2 or all outpatient clinics using Chi-square or Fisher's exact test with Bonferroni-correction for multiple testing.

150x100mm (300 x 300 DPI)

S1 Table. Baseline characteristics per nephrology outpatient clinic.

	Clinic A (n=569)		Clinic B (n=845)		Clinic C (n=1,718)	
	N (%)	Mean (±SD)	N (%)	Mean (±SD)	N (%)	Mean (±SD)
Age (years)	569 (100)	63.4 (±14.6)	845 (100)	65.0 (±15.3)	1,718 (100)	70.5 (±12.6)
< 50 years	98 (17.2)		134 (15.9)		131 (7.6)	
50-80 years	397 (69.8)		581 (68.8)		1,171 (68.2)	
>= 80 years	74 (13.0)		130 (15.4)		416 (24.2)	
Gender (males)	323 (56.8)		446 (52.8)		969 (56.4)	
Diabetes (yes)	145 (25.5)		164 (19.4)		176 (10.2)	
eGFR (MDRD) ml/min	569 (100)	28.4 (±14.2)	845 (100)	35.9 (±14.1)	1,718 (100)	36.5 (±13.1)
Stage 3a	92 (16.2)		255 (30.2)		496 (28.9)	
Stage 3b	166 (29.2)		295 (34.9)		664 (38.7)	
Stage 4	183 (32.2)		219 (25.9)		460 (26.8)	
Stage 5	128 (22.5)		76 (9.0)		98 (5.7)	
SBP (mmHg)	552 (97.0)	131.7 (±18.6)	470 (55.6)	133.4 (±20.7)	1,489 (86.7)	131.7 (±18.2)
Elevated SBP (>140 mmHg)	137 (24.1)	156.2 (±12.7)	143 (16.9)	158.0 (±13.3)	420 (24.4)	154.0 (±11.8)
DBP (mmHg)	552 (97.0)	72.0 (±12.4)	470 (55.6)	76.0 (±10.7)	1,489 (86.7)	76.0 (±10.7)
Low DBP (<70 mmHg)	202 (35.5)	59.4 (±6.9)	100 (11.8)	61.7 (±4.4)	402 (23.4)	62.6 (±5.2)
Total protein (g/24h urine)	513 (90.2)	0.4 [0.1-1.2][a]	244 (28.9)	0.9 [0.3-2.1][a]	557 (32.4)	0.3 [0.1-0.9][a]
Total protein (g/l urine)	515 (90.5)	0.2 [0.1-0.7][a]	574 (67.9)	0.3 [0.1-0.7][a]	1,239 (72.1)	0.2 [0.1-0.4][a]
Proteinuria (>0.5 g/24h or L urine)	215 (37.8)		238 (28.2)		357 (20.8)	
Phosphate (mmol/l)	544 (95.6)	1.20 (±0.33)	623 (73.7)	1.10 (±0.32)	1,439 (83.8)	1.02 (±0.24)
Elevated phosphate (>1.49 mmol/l)	78 (13.7)	1.79 (±0.33)	49 (5.8)	1.87 (±0.40)	45 (2.6)	1.73 (±0.29)
Calcium (mmol/l)	546 (96.0)	2.35 (±0.17)	663 (78.5)	2.34 (±0.14)	1,525 (88.8)	2.37 (±0.12)
Elevated Calcium (>2.54 mmol/l)	35 (6.2)	2.62 (±0.06)	28 (3.3)	2.62 (±0.06)	100 (5.8)	2.62 (±0.09)
Haemoglobin level (mmol/l)	565 (99.3)	7.75 (±1.09)	808 (95.6)	8.0 (±1.2)	1,651 (96.1)	8.1 (±1.1)
Low haemoglobin level (<7.5 mmol/l)	222 (39.0)	6.7 (±0.6)	253 (29.9)	6.7 (±0.6)	458 (26.7)	6.8 (±0.6)

eGFR: estimated glomerular filtration rate; MDRD: Modification of Diet in Renal Disease; SBP: systolic blood pressure; DBP: diastolic blood pressure.
Clinic A and B: university nephrology outpatient clinics; clinic C: non-university nephrology outpatient clinic.
[a] Median with interquartile range.

For peer review only

S2 Table. Baseline table per chronic kidney disease stage per location.

	Clinic A (N=92)		Clinic B (N=255)		Clinic C (N=496)	
**CKD stage 3a**	**N (%)**	**Mean (±SD)**	**N (%)**	**Mean (±SD)**	**N (%)**	**Mean (±SD)**
Age (years)	92 (100)	56.8 (±13.5)	255 (100)	60.5 (±14.9)	496 (100)	65.6 (±13.4)
< 50 years	26 (28.3)		56 (22.0)		69 (13.9)	
50-80 years	62 (67.4)		182 (71.4)		360 (72.6)	
>= 80 years	4 (4.4)		17 (6.7)		67 (13.5)	
Gender (males)	46 (50.0)		135 (52.9)		275 (55.4)	
Diabetes (yes)	14 (15.2)		39 (15.3)		43 (8.7)	
eGFR (MDRD) ml/min	92 (100)	51.1 (±4.2)	255 (100)	52.4 (±4.2)	496 (100)	52.3 (±4.4)
SBP (mmHg)	91 (98.9)	126.3 (±17.4)	122 (47.8)	128.5 (±18.8)	388 (78.2)	130.4 (±16.7)
Elevated SBP (>140 mmHg)	12 (13.0)	157.1 (±15.7)	23 (9.0)	157.4 (±12.9)	84 (16.9)	154.5 (±11.7)
DBP (mmHg)	91 (98.9)	73.4 (±12.5)	122 (47.8)	76.0 (±10.5)	388 (78.2)	77.6 (±10.0)
Low DBP (<70 mmHg)	31 (33.7)	59.8 (±6.1)	22 (8.6)	61.3 (±4.4)	77 (15.5)	63.2 (±5.2)
Total protein (g/24h urine)	84 (91.3)	0.2 [0.1-1.0][a]	62 (24.3)	0.5 [0.3-1.3][a]	132 (26.6)	0.2 [0.1-0.5][a]
Total protein (g/l urine)	84 (91.3)	0.1 [0.1-0.4][a]	165 (65.7)	0.1 [0.0-0.4][a]	303 (61.1)	0.1 [0.1-0.3][a]
Proteinuria (>0.5 g/24h or L urine)	29 (31.5)		52 (20.4)		59 (11.9)	
Phosphate (mmol/l)	83 (90.2)	1.02 (±0.20)	139 (54.5)	0.98 (±0.22)	317 (63.9)	0.94 (±0.19)
Elevated phosphate (>1.49 mmol/l)	0 (0.0)	-	2 (0.8)	1.72 (±0.30)	1 (0.2)	1.63 (-)
Calcium (mmol/l)	84 (91.3)	2.38 (±0.14)	159 (62.4)	2.37 (±0.11)	373 (75.2)	2.38 (±0.11)
Elevated Calcium (>2.54 mmol/l)	6 (6.5)	2.60 (±0.05)	7 (2.7)	2.62 (±0.06)	27 (5.4)	2.62 (±0.08)
Haemoglobin level (mmol/l)	90 (97.8)	8.4 (±1.2)	231 (90.6)	8.6 (±1.0)	450 (90.7)	8.4 (±1.0)
Low haemoglobin level (<7.5 mmol/l)	15 (16.3)	6.6 (±0.5)	29 (11.4)	6.8 (±0.6)	69 (13.9)	6.8 (±0.6)
**CKD stage 3b**	**Clinic A (N=166)**		**Clinic B (N=295)**		**Clinic C (N=664)**	
	**N (%)**	**Mean (±SD)**	**N (%)**	**Mean (±SD)**	**N (%)**	**Mean (±SD)**
Age	166 (100)	63.4 (±14.3)	295 (100)	66.9 (±14.8)	664 (100)	71.2 (±11.5)
< 50 years	33 (19.9)		40 (13.6)		40 (6.0)	
50-80 years	115 (69.3)		200 (67.8)		478 (72.0)	
>= 80 years	18 (10.8)		55 (18.6)		146 (22.0)	
Gender (males)	98 (59.0)		147 (49.8)		369 (55.6)	
Diabetes (yes)	44 (26.5)		54 (18.3)		68 (10.2)	
eGFR (MDRD) ml/min	166 (100)	36.4 (±4.4)	295 (100)	37.6 (±4.1)	664 (100)	37.4 (±4.2)

For peer review only

SBP (mmHg)	163 (98.2)	129.6 (±17.0)	160 (54.2)	131.6 (±21.0)	587 (88.4)	130.7 (±18.3)
Elevated SBP (>140 mmHg)	33 (19.9)	153.8 (±14.2)	49 (16.6)	156.6 (±12.9)	157 (23.4)	153.7 (±11.9)
DBP (mmHg)	163 (98.2)	72.4 (±12.4)	160 (54.2)	75.5 (±10.3)	587 (88.4)	75.4 (±11.1)
Low DBP (<70 mmHg)	61 (36.7)	60.2 (±6.6)	35 (11.9)	61.8 (±4.2)	186 (28.0)	62.8 (±5.0)
Total protein (g/24h urine)	148 (89.2)	0.2 [0.1-0.6][a]	67 (22.7)	0.7 [0.2-2.0][a]	211 (31.8)	0.2 [0.1-0.7][a]
Total protein (g/l urine)	150 (90.4)	0.2 [0.1-0.4][a]	190 (64.4)	0.2 [0.1-0.5][a]	501 (75.5)	0.2 [0.1-0.4][a]
Proteinuria (>0.5 g/24h or L urine)	39 (23.5)		63 (21.4)		122 (18.4)	
Phosphate (mmol/l)	160 (96.4)	1.04 (±0.21)	213 (72.2)	1.02 (±0.22)	586 (88.3)	0.98 (±0.19)
Elevated phosphate (>1.49 mmol/l)	5 (3.0)	1.58 (±0.04)	6 (2.0)	1.80 (±0.28)	6 (0.9)	1.57 (±0.08)
Calcium (mmol/l)	160 (96.4)	2.37 (±0.15)	231 (78.3)	2.34 (±0.12)	607 (91.4)	2.37 (±0.13)
Elevated Calcium (>2.54 mmol/l)	10 (6.0)	2.62 (±0.06)	7 (2.4)	2.61 (±0.06)	44 (6.6)	2.63 (±0.10)
Haemoglobin level (mmol/l)	166 (100)	8.1 (±1.0)	284 (96.3)	8.1 (±1.1)	645 (97.1)	8.2 (±1.0)
Low haemoglobin level (<7.5 mmol/l)	37 (22.2)	6.9 (±0.5)	81 (27.5)	6.8 (±0.5)	146 (22.0)	6.8 (±0.5)
**CKD stage 4**	**Clinic A (N=183)**		**Clinic B (N=219)**		**Clinic C (N=460)**	
	**N (%)**	**Mean (±SD)**	**N (%)**	**Mean (±SD)**	**N (%)**	**Mean (±SD)**
Age	183 (100)	65.3 (±14.6)	219 (100)	67.4 (±15.2)	460 (100)	73.4 (±11.8)
< 50 years	24 (13.1)		26 (11.9)		20 (4.4)	
50-80 years	126 (68.9)		149 (68.0)		281 (61.1)	
>= 80 years	33 (18.0)		44 (20.1)		159 (34.6)	
Gender (males)	98 (53.6)		119 (54.3)		271 (58.9)	
Diabetes (yes)	55 (30.1)		58 (26.5)		52 (11.3)	
eGFR (MDRD) ml/min	183 (100)	22.3 (±4.2)	219 (100)	23.0 (±4.3)	460 (100)	23.4 (±4.2)
SBP (mmHg)	176 (96.2)	132.0 (±19.4)	140 (63.9)	136.8 (±21.0)	419 (91.1)	132.6 (±18.7)
Elevated SBP (>140 mmHg)	48 (26.2)	156.8 (±11.4)	50 (22.8)	159.1 (±13.8)	135 (29.3)	153.5 (±11.5)
DBP (mmHg)	176 (96.2)	71.6 (±12.8)	140 (63.9)	75.1 (±10.9)	419 (91.1)	75.7 (±10.3)
Low DBP (<70 mmHg)	66 (36.1)	59.0 (±7.6)	38 (17.4)	61.7 (±4.7)	110 (23.9)	62.6 (±5.1)
Total protein (g/24h urine)	163 (89.1)	0.4 [0.1-1.0][a]	75 (34.2)	0.9 [0.3-2.3][a]	165 (35.9)	0.4 [0.2-1.3][a]
Total protein (g/l urine)	163 (89.1)	0.2 [0.1-0.6][a]	162 (74.0)	0.3 [0.2-0.8][a]	362 (78.7)	0.2 [0.1-0.6][a]
Proteinuria (>0.5 g/24h or L urine)	63 (34.4)		77 (35.2)		128 (27.8)	
Phosphate (mmol/l)	176 (96.2)	1.18 (±0.23)	200 (91.3)	1.14 (±0.32)	439 (95.4)	1.05 (±0.2)
Elevated phosphate (>1.49 mmol/l)	16 (8.7)	1.62 (±0.11)	17 (7.8)	1.84 (±0.47)	8 (1.7)	1.66 (±0.11)
Calcium (mmol/l)	176 (96.2)	2.35 (±0.20)	199 (90.9)	2.34 (±0.16)	447 (97.2)	2.36 (±0.12)
Elevated Calcium (>2.54 mmol/l)	13 (7.1)	2.61 (±0.07)	9 (4.1)	2.65 (±0.07)	24 (5.2)	2.62 (±0.06)

For peer review only

Haemoglobin level (mmol/l)	182 (99.5)	7.6 (±1.0)	217 (99.1)	7.7 (±1.2)	458 (99.6)	7.8 (±1.0)
Low haemoglobin level (<7.5 mmol/l)	82 (44.8)	6.7 (±0.7)	89 (40.6)	6.6 (±0.6)	175 (38.0)	6.7 (±0.6)
**CKD stage 5**	**Clinic A (N=128)**		**Clinic B (N=76)**		**Clinic (N=98)**	
	**N (%)**	**Mean (±SD)**	**N (%)**	**Mean (±SD)**	**N (%)**	**Mean (±SD)**
Age	128 (100)	65.5 (±14.7)	100 (76)	65.5 (±15.9)	100 (98)	76.9 (±10.6)
< 50 years	15 (11.7)		12 (15.8)		2.0 (2.0)	
50-80 years	94 (73.4)		40 (65.8)		52 (53.1)	
>= 80 years	19 (14.8)		14 (18.4)		44 (44.9)	
Gender (males)	81 (63.3)		45 (59.2)		54 (55.1)	
Diabetes (yes)	32 (25.0)		13 (17.1)		13 (13.3)	
eGFR (MDRD) ml/min	128 (100)	10.5 (±2.6)	76 (100)	11.2 (±2.4)	98 (100)	11.9 (±2.6)
SBP (mmHg)	122 (95.3)	137.9 (±18.6)	48 (63.2)	142.1 (±19.8)	95 (96.9)	139.2 (±19.7)
Elevated SBP (>140 mmHg)	44 (34.4)	157.1 (±12.2)	21 (27.6)	159.6 (±14.2)	44 (44.9)	155.9 (±12.2)
DBP (mmHg)	122 (95.3)	71.1 (±11.6)	48 (63.2)	80.6 (±11.4)	95 (96.9)	74.4 (±11.9)
Low DBP (<70 mmHg)	44 (34.4)	58.5 (±6.7)	5 (6.6)	62.2 (±4.1)	29 (29.6)	60.2 (±6.4)
Total protein (g/24h urine)	118 (92.2)	1.3 [0.5-2.7][a]	40 (52.6)	1.9 [0.9-4.1][a]	49 (50.0)	1.0 [0.4-2.6][a]
Total protein (g/l urine)	118 (92.2)	0.7 [0.3-1.4][a]	57 (75.0)	1.4 [0.5-2.4][a]	73 (74.5)	0.6 [0.3-1.1][a]
Proteinuria (>0.5 g/24h or L urine)	84 (65.6)		57 (60.5)		48 (49.0)	
Phosphate (mmol/l)	125 (97.7)	1.55 (±0.39)	71 (93.4)	1.47 (±0.42)	97 (99.0)	1.39 (±0.36)
Elevated phosphate (>1.49 mmol/l)	57 (44.5)	1.86 (±0.36)	24 (31.6)	1.92 (±0.39)	30 (30.6)	1.79 (±0.34)
Calcium (mmol/l)	126 (98.4)	2.31 (±0.16)	74 (97.4)	2.27 (±0.19)	98 (100)	2.32 (±0.14)
Elevated Calcium (>2.54 mmol/l)	13 (10.2)	2.65 (±0.07)	5 (6.6)	2.63 (±0.08)	5 (5.1)	2.66 (±0.11)
Haemoglobin level (mmol/l)	127 (99.2)	7.0 (±0.8)	76 (100)	7.0 (±1.2)	98 (100)	7.0 (±0.9)
Low haemoglobin level (<7.5 mmol/l)	88 (68.8)	6.6 (±0.5)	54 (71.1)	6.4 (±0.8)	68 (69.4)	6.6 (±0.7)

CKD: chronic kidney disease; SD: standard deviation; eGFR: estimated glomerular filtration rate; MDRD: Modification of Diet in Renal Disease; SBP: systolic blood pressure; DBP: diastolic blood pressure.

Clinic A and B: university nephrology outpatient clinics; clinic C: non-university nephrology outpatient clinic.

[a] Median with interquartile range.

For peer review only

S3 Table. Differences between nephrology outpatient clinics per indicator stratified for chronic kidney disease stage.

Indicator 1 - Antihypertensive use

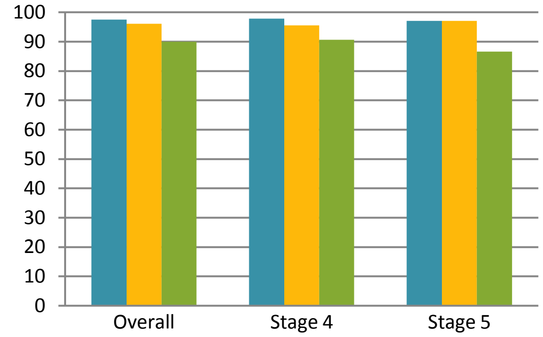

	Overall	Stage 4	Stage 5
Clinic A	97.6	97.9	97.1
Clinic B	96.1	95.6	97.1
Clinic C	90.0	90.6	86.7

Overall	*p-value χ²*
Clinic A vs. Clinic B	0.316
Clinic A vs. Clinic C	**0.003**
Clinic B vs. Clinic C	0.095
CKD stage 4	
Clinic A vs. Clinic B	0.406†
Clinic A vs. Clinic C	**0.011**
Clinic B vs. Clinic C	0.196
CKD stage 5	
Clinic A vs. Clinic B	1.000†
Clinic A vs. Clinic C	0.103†
Clinic B vs. Clinic C	0.306†

Indicator 2 - RAASi with proteinuria

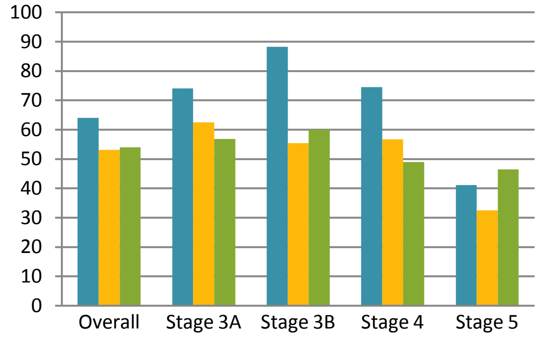

	Overall	Stage 3A	Stage 3B	Stage 4	Stage 5
Clinic A	64.0	74.1	88.2	74.5	41.1
Clinic B	53.1	62.5	55.4	56.7	32.5
Clinic C	54.0	56.9	60.0	49.0	46.4

Overall	*p-value χ²*
Clinic A vs. Clinic B	0.030
Clinic A vs. Clinic C	**0.003**
Clinic B vs. Clinic C	0.503
CKD stage 3a	
Clinic A vs. Clinic B	0.190
Clinic A vs. Clinic C	0.095
Clinic B vs. Clinic C	0.675
CKD stage 3b	
Clinic A vs. Clinic B	**0.002**
Clinic A vs. Clinic C	**0.001**
Clinic B vs. Clinic C	0.819
CKD stage 4	
Clinic A vs. Clinic B	0.089
Clinic A vs. Clinic C	**0.001**
Clinic B vs. Clinic C	0.117
CKD stage 5	
Clinic A vs. Clinic B	0.209
Clinic A vs. Clinic C	0.243
Clinic B vs. Clinic C	0.923

For peer review only

Indicator 4 - RAASi and diuretics

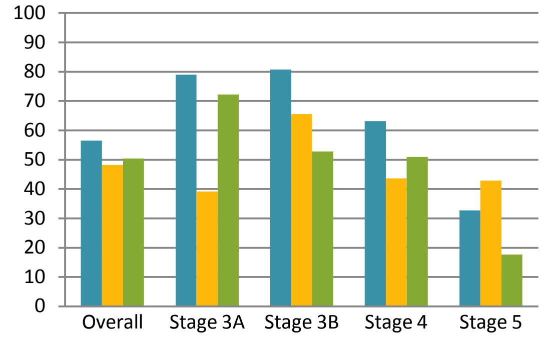

	Overall	Stage 3A	Stage 3B	Stage 4	Stage 5
Clinic A	56.5	78.9	80.8	63.2	32.7
Clinic B	48.2	39.1	65.5	43.6	42.9
Clinic C	50.4	72.2	52.8	50.9	17.6

Overall	*p-value χ²*
Clinic A vs. Clinic B	0.184
Clinic A vs. Clinic C	0.081
Clinic B vs. Clinic C	0.786
CKD stage 3a	
Clinic A vs. Clinic B	**0.014**
Clinic A vs. Clinic C	1.000
Clinic B vs. Clinic C	**0.014**
CKD stage 3b	
Clinic A vs. Clinic B	0.068
Clinic A vs. Clinic C	**0.002**
Clinic B vs. Clinic C	0.235
CKD stage 4	
Clinic A vs. Clinic B	0.245
Clinic A vs. Clinic C	0.337
Clinic B vs. Clinic C	0.766
CKD stage 5	
Clinic A vs. Clinic B	0.586
Clinic A vs. Clinic C	0.033
Clinic B vs. Clinic C	0.021

Indicator 6 - Statin use

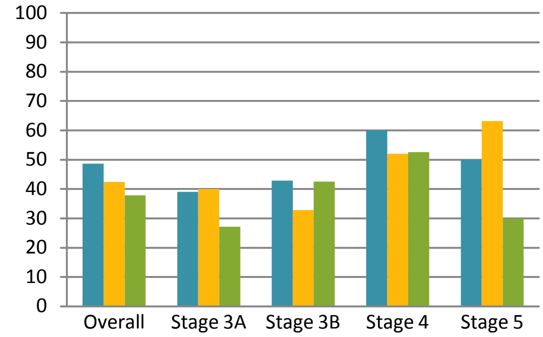

	Overall	Stage 3A	Stage 3B	Stage 4	Stage 5
Clinic A	48.7	39.0	42.9	60.0	50.0
Clinic B	42.4	40.0	32.9	52.0	63.2
Clinic C	37.9	27.2	42.5	52.6	30.0

Overall	*p-value χ²*
Clinic A vs. Clinic B	0.205
Clinic A vs. Clinic C	**0.015**
Clinic B vs. Clinic C	0.277
CKD stage 3a	
Clinic A vs. Clinic B	0.916
Clinic A vs. Clinic C	0.140
Clinic B vs. Clinic C	0.042
CKD stage 3b	
Clinic A vs. Clinic B	0.266
Clinic A vs. Clinic C	0.966
Clinic B vs. Clinic C	0.189
CKD stage 4	
Clinic A vs. Clinic B	0.409
Clinic A vs. Clinic C	0.395
Clinic B vs. Clinic C	0.950
CKD stage 5	
Clinic A vs. Clinic B	0.340
Clinic A vs. Clinic C	0.309[†]
Clinic B vs. Clinic C	0.128[†]

Indicator 7 - Phosphate binder use

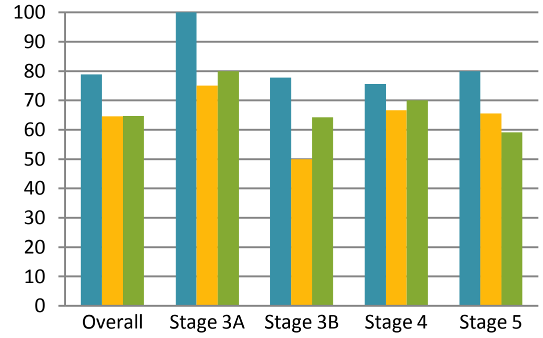

	Overall	Stage 3A	Stage 3B	Stage 4	Stage 5
Clinic A	78.9	100.0	77.8	75.6	80.0
Clinic B	64.6	75.0	50.0	66.7	65.5
Clinic C	64.7	80.0	64.3	70.0	59.1

Overall	*p-value χ²*
Clinic A vs. Clinic B	**0.016**

Indicator 10 - Dual RAAS blockade

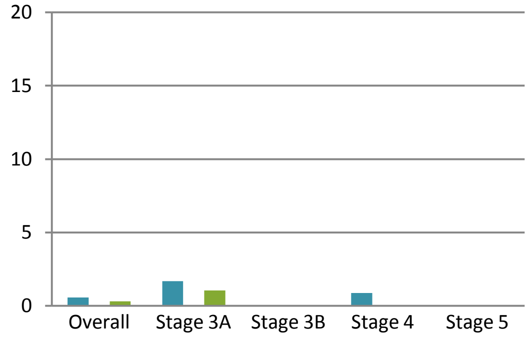

	Overall	Stage 3A	Stage 3B	Stage 4	Stage 5
Clinic A	0.6	1.7	0.0	0.9	0.0
Clinic B	0.0	0.0	0.0	0.0	0.0
Clinic C	0.3	1.0	0.0	0.0	0.0

Overall	*p-value χ²*
Clinic A vs. Clinic B	0.218[†]

Clinic A vs. Clinic C	0.030
Clinic B vs. Clinic C	0.849
CKD stage 3a	
Clinic A vs. Clinic B	1.000†
Clinic A vs. Clinic C	1.000†
Clinic B vs. Clinic C	1.000†
CKD stage 3b	
Clinic A vs. Clinic B	0.650†
Clinic A vs. Clinic C	1.000†
Clinic B vs. Clinic C	0.692†
CKD stage 4	
Clinic A vs. Clinic B	0.191
Clinic A vs. Clinic C	0.548
Clinic B vs. Clinic C	0.620
CKD stage 5	
Clinic A vs. Clinic B	0.067
Clinic A vs. Clinic C	**0.013**
Clinic B vs. Clinic C	0.575

Clinic A vs. Clinic C	0.612†
Clinic B vs. Clinic C	0.530†
CKD stage 3a	
Clinic A vs. Clinic B	0.323†
Clinic A vs. Clinic C	0.558†
Clinic B vs. Clinic C	0.521†
CKD stage 3b	
Clinic A vs. Clinic B	No prescriptions
Clinic A vs. Clinic C	No prescriptions
Clinic B vs. Clinic C	No prescriptions
CKD stage 4	
Clinic A vs. Clinic B	1.000†
Clinic A vs. Clinic C	0.391†
Clinic B vs. Clinic C	No prescriptions
CKD stage 5	
Clinic A vs. Clinic B	No prescriptions
Clinic A vs. Clinic C	No prescriptions
Clinic B vs. Clinic C	No prescriptions

Indicator 11 - Vitamin D use

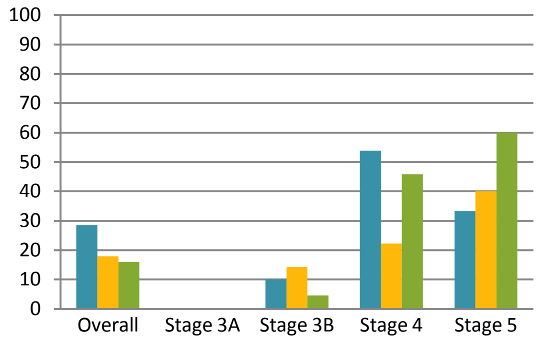

	Overall	Stage 3A	Stage 3B	Stage 4	Stage 5
Clinic A	28.6	0.0	10.0	53.8	33.3
Clinic B	17.9	0.0	14.3	22.2	40.0
Clinic C	16.0	0.0	4.5	45.8	60.0

Overall	*p-value* χ^2
Clinic A vs. Clinic B	0.321
Clinic A vs. Clinic C	0.105
Clinic B vs. Clinic C	0.779†
CKD stage 3a	
Clinic A vs. Clinic B	No prescriptions
Clinic A vs. Clinic C	No prescriptions
Clinic B vs. Clinic C	No prescriptions
CKD stage 3b	
Clinic A vs. Clinic B	1.000†
Clinic A vs. Clinic C	0.466†
Clinic B vs. Clinic C	0.364†
CKD stage 4	
Clinic A vs. Clinic B	0.203†
Clinic A vs. Clinic C	0.642
Clinic B vs. Clinic C	0.263†
CKD stage 5	
Clinic A vs. Clinic B	1.000†
Clinic A vs. Clinic C	0.567†

Indicator 12 - ESA use

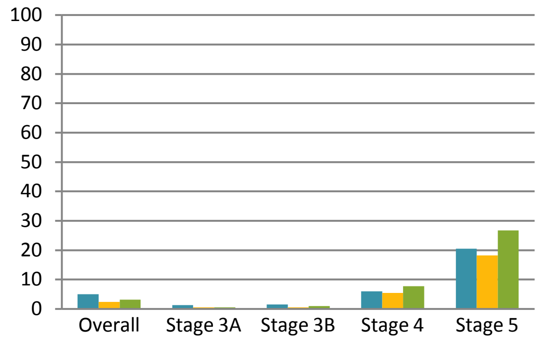

	Overall	Stage 3A	Stage 3B	Stage 4	Stage 5
Clinic A	5.0	1.3	1.6	6.0	20.5
Clinic B	2.3	0.5	0.5	5.5	18.2
Clinic C	3.1	0.5	1.0	7.8	26.7

Overall	*p-value* χ^2
Clinic A vs. Clinic B	0.034
Clinic A vs. Clinic C	0.100
Clinic B vs. Clinic C	0.375
CKD stage 3a	
Clinic A vs. Clinic B	0.469†
Clinic A vs. Clinic C	0.417†
Clinic B vs. Clinic C	1.000†
CKD stage 3b	
Clinic A vs. Clinic B	0.562†
Clinic A vs. Clinic C	0.637†
Clinic B vs. Clinic C	0.678†
CKD stage 4	
Clinic A vs. Clinic B	0.864
Clinic A vs. Clinic C	0.558
Clinic B vs. Clinic C	0.398
CKD stage 5	
Clinic A vs. Clinic B	1.000†
Clinic A vs. Clinic C	0.548

Clinic B vs. Clinic C	1.000†

Clinic B vs. Clinic C	0.473

Indicator 13 - NSAIDs use

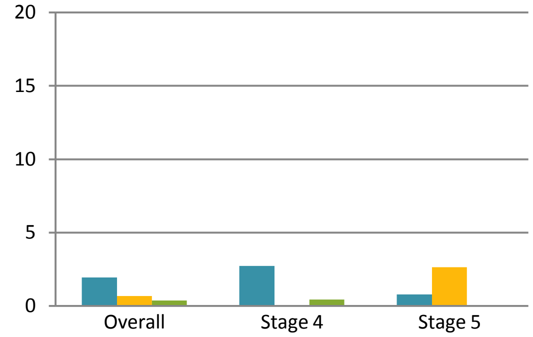

	Overall	Stage 4	Stage 5
Clinic A	1.9	2.7	0.8
Clinic B	0.7	0.0	2.6
Clinic C	0.4	0.4	0.0

Overall	*p-value* χ^2
Clinic A vs. Clinic B	0.287†
Clinic A vs. Clinic C	0.028†
Clinic B vs. Clinic C	0.612†
CKD stage 4	
Clinic A vs. Clinic B	0.019†
Clinic A vs. Clinic C	0.022†
Clinic B vs. Clinic C	1.000†
CKD stage 5	
Clinic A vs. Clinic B	0.557†
Clinic A vs. Clinic C	1.000†
Clinic B vs. Clinic C	0.189†

Indicator 14 - Metformin use

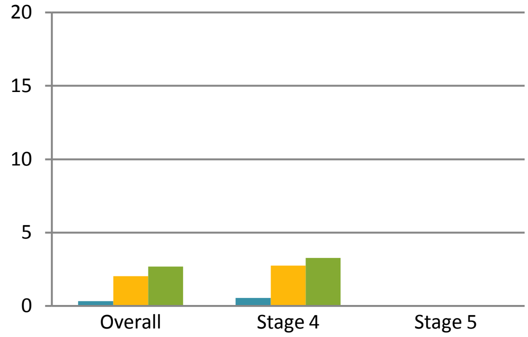

	Overall	Stage 4	Stage 5
Clinic A	0.3	0.5	0.0
Clinic B	2.0	2.7	0.0
Clinic C	2.7	3.3	0.0

Overall	*p-value* χ^2
Clinic A vs. Clinic B	0.062
Clinic A vs. Clinic C	**0.013**
Clinic B vs. Clinic C	0.558
CKD stage 4	
Clinic A vs. Clinic B	0.132†
Clinic A vs. Clinic C	0.050†
Clinic B vs. Clinic C	0.714
CKD stage 5	
Clinic A vs. Clinic B	No prescriptions
Clinic A vs. Clinic C	No prescriptions
Clinic B vs. Clinic C	No prescriptions

Indicator 15 - HD digoxin use

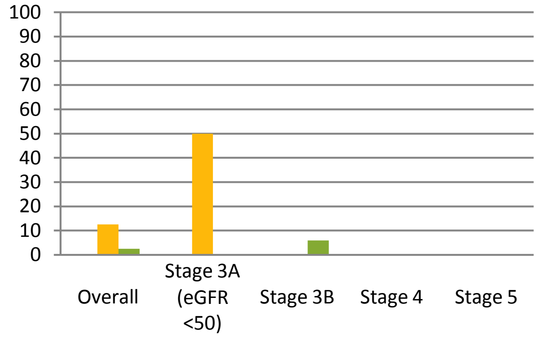

	Overall	Stage 3A (eGFR <50)	Stage 3B	Stage 4	Stage 5
Clinic A	0.0	n.a.	0.0	0.0	0.0
Clinic B	12.5	50.0	0.0	0.0	n.a.
Clinic C	2.4	0.0	5.9	0.0	0.0

Overall	*p-value* χ^2
Clinic A vs. Clinic B	1.000†
Clinic A vs. Clinic C	1.000†
Clinic B vs. Clinic C	0.202†
CKD stage 3a	
Clinic A vs. Clinic B	No prescriptions
Clinic A vs. Clinic C	No prescriptions
Clinic B vs. Clinic C	0.333†
CKD stage 3b	

Indicator 16 - NSAIDs, RAAS and diuretics

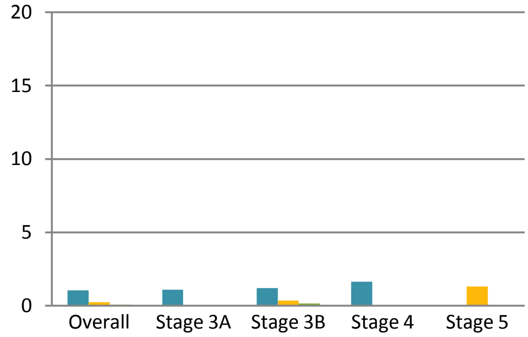

	Overall	Stage 3A	Stage 3B	Stage 4	Stage 5
Clinic A	1.1	1.1	1.2	1.6	0.0
Clinic B	0.2	0.0	0.3	0.0	1.3
Clinic C	0.1	0.0	0.2	0.0	0.0

Overall	*p-value* χ^2
Clinic A vs. Clinic B	0.067†
Clinic A vs. Clinic C	**0.001†**
Clinic B vs. Clinic C	0.254†
CKD stage 3a	
Clinic A vs. Clinic B	0.265†
Clinic A vs. Clinic C	0.156†
Clinic B vs. Clinic C	No prescriptions
CKD stage 3b	

Clinic A vs. Clinic B	No prescriptions	Clinic A vs. Clinic B	0.295†
Clinic A vs. Clinic C	1.000†	Clinic A vs. Clinic C	0.104†
Clinic B vs. Clinic C	1.000†	Clinic B vs. Clinic C	0.521†
CKD stage 4		*CKD stage 4*	
Clinic A vs. Clinic B	No prescriptions	Clinic A vs. Clinic B	0.093†
Clinic A vs. Clinic C	No prescriptions	Clinic A vs. Clinic C	0.023†
Clinic B vs. Clinic C	No prescriptions	Clinic B vs. Clinic C	1.000†
CKD stage 5		*CKD stage 5*	
Clinic A vs. Clinic B	No prescriptions	Clinic A vs. Clinic B	0.373†
Clinic A vs. Clinic C	No prescriptions	Clinic A vs. Clinic C	No prescriptions
Clinic B vs. Clinic C	No prescriptions	Clinic B vs. Clinic C	0.437†

CKD: chronic kidney disease; RAAS: renin-angiotensin-aldosterone system; ESA: erythropoiesis-stimulating agent; NSAID: non-steroidal anti-inflammatory drug.

Clinic A and B: university nephrology outpatient clinics; clinic C: non-university nephrology outpatient clinic.

Indicators 3, 5, 8 and 9 were excluded because of insufficient validity.

† Fisher's exact because of <5 expected number of patients in one or more cells.

For peer review only

STROBE 2007 (v4) Statement—Checklist of items that should be included in reports of *cohort studies*

Section/Topic	Item #	Recommendation	Reported on page #
**Title and abstract**	1	(*a*) Indicate the study's design with a commonly used term in the title or the abstract	1
		(*b*) Provide in the abstract an informative and balanced summary of what was done and what was found	2-3
**Introduction**			
Background/rationale	2	Explain the scientific background and rationale for the investigation being reported	3-4
Objectives	3	State specific objectives, including any prespecified hypotheses	4
**Methods**			
Study design	4	Present key elements of study design early in the paper	4
Setting	5	Describe the setting, locations, and relevant dates, including periods of recruitment, exposure, follow-up, and data collection	5
Participants	6	(*a*) Give the eligibility criteria, and the sources and methods of selection of participants. Describe methods of follow-up	4-5
		(*b*) For matched studies, give matching criteria and number of exposed and unexposed	NA
Variables	7	Clearly define all outcomes, exposures, predictors, potential confounders, and effect modifiers. Give diagnostic criteria, if applicable	5-7
Data sources/ measurement	8*	For each variable of interest, give sources of data and details of methods of assessment (measurement). Describe comparability of assessment methods if there is more than one group	7
Bias	9	Describe any efforts to address potential sources of bias	7
Study size	10	Explain how the study size was arrived at	4-5
Quantitative variables	11	Explain how quantitative variables were handled in the analyses. If applicable, describe which groupings were chosen and why	7
Statistical methods	12	(*a*) Describe all statistical methods, including those used to control for confounding	7
		(*b*) Describe any methods used to examine subgroups and interactions	7
		(*c*) Explain how missing data were addressed	7
		(*d*) If applicable, explain how loss to follow-up was addressed	NA
		(*e*) Describe any sensitivity analyses	NA
**Results**			

For peer review only

Participants	13*	(a) Report numbers of individuals at each stage of study—eg numbers potentially eligible, examined for eligibility, confirmed eligible, included in the study, completing follow-up, and analysed	8
		(b) Give reasons for non-participation at each stage	NA
		(c) Consider use of a flow diagram	NA
Descriptive data	14*	(a) Give characteristics of study participants (eg demographic, clinical, social) and information on exposures and potential confounders	8-9
		(b) Indicate number of participants with missing data for each variable of interest	9
		(c) Summarise follow-up time (eg, average and total amount)	NA
Outcome data	15*	Report numbers of outcome events or summary measures over time	10
Main results	16	(*a*) Give unadjusted estimates and, if applicable, confounder-adjusted estimates and their precision (eg, 95% confidence interval). Make clear which confounders were adjusted for and why they were included	10-11
		(*b*) Report category boundaries when continuous variables were categorized	NA
		(*c*) If relevant, consider translating estimates of relative risk into absolute risk for a meaningful time period	NA
Other analyses	17	Report other analyses done—eg analyses of subgroups and interactions, and sensitivity analyses	10-11
**Discussion**			
Key results	18	Summarise key results with reference to study objectives	11-12
**Limitations**			
Interpretation	20	Give a cautious overall interpretation of results considering objectives, limitations, multiplicity of analyses, results from similar studies, and other relevant evidence	13-14
Generalisability	21	Discuss the generalisability (external validity) of the study results	14
**Other information**			
Funding	22	Give the source of funding and the role of the funders for the present study and, if applicable, for the original study on which the present article is based	15

*Give information separately for cases and controls in case-control studies and, if applicable, for exposed and unexposed groups in cohort and cross-sectional studies.

Note: An Explanation and Elaboration article discusses each checklist item and gives methodological background and published examples of transparent reporting. The STROBE checklist is best used in conjunction with this article (freely available on the Web sites of PLoS Medicine at http://www.plosmedicine.org/, Annals of Internal Medicine at http://www.annals.org/, and Epidemiology at http://www.epidem.com/). Information on the STROBE Initiative is available at www.strobe-statement.org.

For peer review only
